# Ultra‐Narrowband Blue Multi‐Resonance Thermally Activated Delayed Fluorescence Materials

**DOI:** 10.1002/advs.202205070

**Published:** 2022-11-17

**Authors:** Susumu Oda, Bungo Kawakami, Masaru Horiuchi, Yuki Yamasaki, Ryosuke Kawasumi, Takuji Hatakeyama

**Affiliations:** ^1^ Department of Chemistry Graduate School of Science and Technology Kwansei Gakuin University 2‐1 Gakuen, Sanda Hyogo 669‐1337 Japan; ^2^ Department of Chemistry Graduate School of Science Kyoto University Sakyo‐ku Kyoto 606‐8502 Japan; ^3^ SK JNC Japan Co., Ltd. 25‐1 Goi Kaigan Ichihara Chiba 290‐8551 Japan

**Keywords:** fluorine, multi‐resonance effect, narrowband emission, organic light‐emitting diodes, thermally activated delayed fluorescence

## Abstract

Ultra‐narrowband blue multi‐resonance‐induced thermally activated delayed fluorescence (MR‐TADF) materials (**V‐DABNA** and **V‐DABNA‐F**), consisting of three DABNA subunits possessing phenyl or 2,6‐difluorophenyl substituents on the peripheral nitrogen atoms are synthesized by one‐shot triple borylation. Benefiting from the inductive effect of fluorine atoms, the emission maximum of **V‐DABNA‐F** (464 nm) is blueshifted from that of the parent **V‐DABNA** (481 nm), while maintaining a small full width at half maximum (FWHM, 16 nm) and a high rate constant for reverse intersystem crossing (6.5 × 10^5^ s^−1^). The organic light‐emitting diodes (OLEDs) using **V‐DABNA** and **V‐DABNA‐F** as emitters are fabricated by vapor deposition and exhibit blue emission at 483 and 468 nm with small FWHMs of 17 and 15 nm, corresponding to Commission Internationale d’Éclairage coordinates of (0.09, 0.27) and (0.12, 0.10), respectively. Both devices achieve high external quantum efficiencies of 26.2% and 26.6% at the maximum with minimum efficiency roll‐offs of 0.9% and 3.2%, respectively, even at 1000 cd m^−2^, which are record‐setting values for blue MR‐TADF OLEDs.

## Graphical abstract

Ultra‐narrowband blue multi‐resonance‐induced thermally activated delayed fluorescence (MR‐TADF) materials (**V‐DABNA** and **V‐DABNA‐F**) are synthesized by one‐shot borylation. The organic light‐emitting diodes (OLEDs) achieve high external quantum efficiencies of 26.2%–26.6% at the maximum with minimum efficiency roll‐offs of 0.9%–3.2% even at 1000 cd m^−2^, which are record‐setting values for blue MR‐TADF OLEDs.

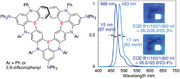

## 1 Introduction

Organic light‐emitting diodes (OLEDs) using thermally activated delayed fluorescence (TADF) emitters have offered high electroluminance efficiency without the use of precious metals by harvesting both singlet and triplet excitons through reverse intersystem crossing (RISC).^[^ ^1^, ^2^ ^]^ Conventional TADF materials include donor and acceptor groups, which separate the highest occupied molecular orbital (HOMO) and lowest unoccupied molecular orbitals (LUMO), reduce the singlet–triplet energy gap (Δ*E*_ST_) and promote the RISC process. However, the D‐A type TADF molecules exhibit broad emission due to the structural relaxation in the excited states and suffer from low color purity, which hinders their widespread practical application.

To overcome this drawback, we have developed TADF materials with a new molecular design based on the multi‐resonance (MR) effects of boron and nitrogen atoms, which enable the alternate localization of HOMOs and LUMOs at different carbon atoms on the same benzene ring.^[^ ^3^ ^]^ This unique HOMO–LUMO separation successfully suppressed not only the structural relaxation, but also the vibronic coupling between the S_1_–S_0_ transition and stretching/scissoring vibration to achieve both high photoluminescence quantum yield (PLQY) and high color purity. Inspired by our first report on the MR‐TADF material (**DAB** **NA**),^[^ ^3b^ ^]^ intensive studies have been carried out on the structural modification,^[^ ^3^, ^4^, ^5^, ^6^, ^7^ ^]^ revealing that *π*‐extension is an effective way to improve color purity and enhance the RISC rate constant (*k*_RISC_).^[^ ^4^ ^]^ For example, we have reported an ultrapure deep‐blue MR‐TADF material (***ν*** **‐** **DA** **BN** **A**), exhibiting narrowband emission at 467 nm with a full width at half maximum (FWHM) of 18 nm and *k*_RISC_ of 2.0 × 10^5^ s^−1^.^[^ ^3d^ ^]^ Subsequently, we have successfully synthesized BN‐embedded expanded helicene (**V‐DABNA‐Mes**) by one‐shot triple borylation.^[^ ^3i^ ^]^ Benefiting from the large *π*‐helical structure, **V‐DABNA‐Mes** exhibited ultra‐narrowband blue MR‐TADF with an extremely small FWHM of 16 nm and a high *k*_RISC_ of 4.4 × 10^5^ s^−1^. However, the large molecular weight of **V‐DABNA‐Mes**, owing to the mesityl groups, hampered the fabrication of an OLED device by vapor deposition. Herein, we report a synthesis of V‐DABNA derivative (**V‐DABNA**), which enabled the fabrication of a vacuum‐deposited OLED device with improved OLED performance including external quantum efficiency (EQE) and operational lifetime. Furthermore, we synthesized a fluorine‐substituted derivative (V‐DABNA‐F) to realize a hypsochromic shift of V‐DABNA‐derived sky‐blue emission without losing color purity using the inductive effect of fluorine atoms^[^ ^5^ ^]^ and developed a highly efficient ultrapure deep‐blue OLED device using it as an emitter.

## 2 Results and Discussion

**Figure 1:**
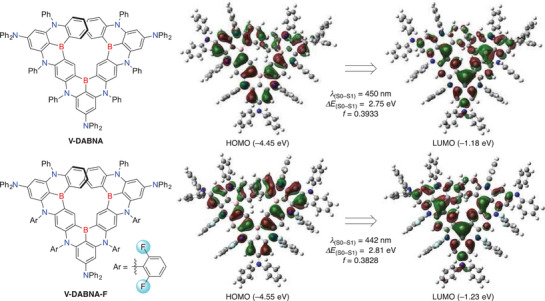
Chemical structures and Kohn–Sham frontier orbitals of **V‐DABNA** and **V‐DABNA‐F** with the oscillator strength (*f*) and S_0_–S_1_ transition energies (Δ*E*, *λ*) at the B3LYP/6‐31G(d) level of theory.

According to the density functional theory (DFT) calculations at the B3LYP/6‐31G(d) level of theory (**Figure** **1**), both HOMO and LUMO energy levels of **V‐DABNA‐F** (−4.55 and −1.23 eV, respectively) are lower than those of **V‐DABNA** (−4.45 and −1.18 eV, respectively). This is attributed to the inductive effect of fluorine atoms, which reduces the electron density of nitrogen atoms, resulting in a larger HOMO–LUMO energy gap. Thus, the S_0_–S_1_ transition energy of **V‐DABNA‐F** (2.81 eV) is larger than that of **V‐DABNA** (2.75 eV), suggesting blueshifted emission.^[^ ^6^ ^]^ Note that the transition energy of the 3,5‐substituted derivative is 2.78 eV due to the weaker inductive effect (Figure S5, Supporting Information). Furthermore, time‐dependent DFT calculations on **V‐DABNA‐core** and **V‐DABNA‐F‐core** revealed a small Δ*E*_ST_ (39 and −9.5 meV) and large spin–orbit coupling matrix elements (〈S_1_|Ĥ_SOC_|T_1_〉: 0.009 and 0.005 cm^−1^, 〈S_1_|Ĥ_SOC_|T_2_〉: 0.084 and 0.091 cm^−1^), suggesting a high *k*_RISC_ based on the relationship of *k*_RISC_ with 〈S_n_|Ĥ_SOC_|T_n_〉 and Δ*E*_ST_ (*k*_RISC_ ∝ 〈S_n_|Ĥ_SOC_|T_n_〉^2^/Δ*E*_ST_) (Figure S3, Supporting Information).^[^ ^8^ ^]^

**Scheme 1:**
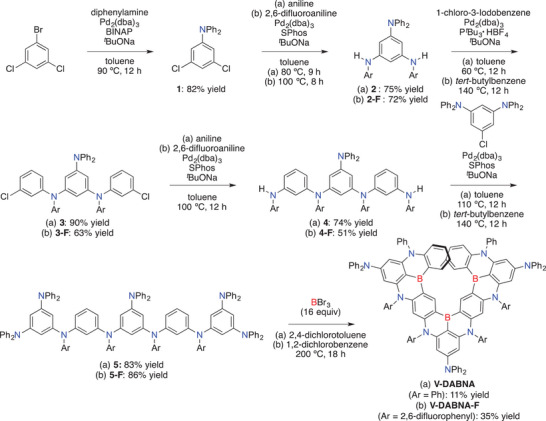
Synthesis of a) **V‐DABNA** and b) **V‐DABNA‐F**.

Syntheses of **V‐DABNA** and **V‐DABNA‐F** are shown in **Scheme** **1**. The precursor **5** was prepared by Buchwald–Hartwig coupling in five steps from commercially available 1‐bromo‐3,5‐dichlorobenzene. In the presence of BBr_3_ (16 equiv), one‐shot triple borylation of **5** occurred at 200 °C to afford **V‐DABNA** in 11% yield. Moreover, this protocol was applied to fluorine‐substituted precursor **5‐F**, affording **V‐DABNA‐F** in 35% yield. The higher yield of **V‐DABNA‐F** is attributed to the 2,6‐difluorophenyl groups, which suppressed the borylation at undesired reaction sites (Figure S4, Supporting Information).

**Figure 2:**
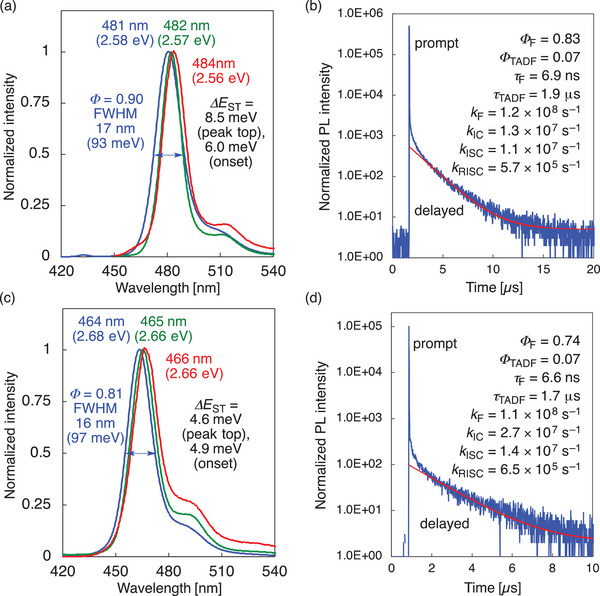
Photophysical properties of **V‐DABNA** and **V‐DABNA‐F** in poly(methyl methacrylate) (PMMA) (1 wt%‐doped films). Fluorescence spectra of a) **V‐DABNA** and c) **V‐DABNA‐F** at 300 K (blue) and at 77 K with (red)/without (green) a delay time of 25 ms. Transient PL decay curves for b) **V‐DABNA** and d) **V‐DABNA‐F**. The red curves represent the single exponential fitting data [background = (b) 5, (d) 2].

The photophysical properties of **V‐DABNA** and **V‐DABNA‐F** were measured in 1 wt%‐doped poly(methyl methacrylate) (PMMA) films (**Figure** **2** and **Table** **1**). Notably, **V‐DABNA** exhibited sky‐blue emission at 481 nm with small FWHM of 17 nm and high PLQY of 90% (Figure 2a). The overlap of terminal phenyl ring may contribute to the larger redshift from ***ν*‐DABNA** (467 nm) to **V‐DABNA** (481 nm) than that from **DABNA‐1** (460 nm) to ***ν*‐DABNA** (467 nm).^[^ ^3b,d^ ^]^ In addition, **V‐DABNA‐F** exhibited deep‐blue emission at 464 nm, which was significantly blueshifted with respect to the parent **V‐DABNA**, as predicted by time‐dependent DFT calculations (Figure 2c). The small shoulder peaks around 510 and 495 nm in **V‐DABNA** and **V‐DABNA‐F**, respectively, are attributed to the vibronic coupling between S_1_–S_0_ transition and stretching/scissoring vibration, which are mostly suppressed by the MR effect. The FWHM and PLQY of **V‐DABNA‐F** are 16 nm and 81%, respectively, which are similar to those of **V‐DABNA**, indicating that the MR effect is maintained despite the incorporation of fluorine atoms. Based on the onset energies of fluorescence and phosphorescence, the Δ*E*_ST_ of 6.0 and 4.9 meV was determined for **V‐DABNA** and **V‐DABNA‐F**, respectively. The transient decay curves of **V‐DABNA** and **V‐DABNA‐F** showed two components with prompt lifetimes of 6.9 and 6.6 ns and delayed lifetimes of 1.9 and 1.7 µs, respectively (Figure 2b,d). Based on the obtained values of quantum yields and lifetimes, the rate constants of fluorescence (*k*_F_), internal conversion (*k*_IC_), intersystem crossing (*k*_ISC_), and *k*_RISC_ were determined by Adachi's method (Figure 2b,d).^[^ ^9^ ^]^ The *k*_RISC_ values of **V‐DABNA** and **V‐DABNA‐F** are 5.7 × 10^5^ and 6.5 × 10^5^ s^−1^, respectively, which are higher than that of **V‐DABNA‐Mes** (4.4 × 10^5^ s^−1^) (Table 1). Although the *k*_F_ values are comparable between **V‐DABNA** and **V‐DABNA‐F** (1.2 × 10^8^ and 1.1 × 10^8^ s^−1^, respectively), the *k*_IC_ value of **V‐DABNA‐F** (2.7 × 10^7^ s^−1^) is larger than that of **V‐DABNA** (1.3 × 10^7^ s^−1^), resulting in the lower PLQY in PMMA. The higher PLQY of 0.91 was observed for **V‐DABNA‐F** in toluene solution (Table S3, Supporting Information) suggests that aggregation in PMMA causes bimolecular quenching^[^ ^10^ ^]^ or that the large dielectric constant of PMMA accelerates the IC process.

**Table 1:** Photophysical properties of **V‐DABNA** and **V‐DABNA‐F** in poly(methyl methacrylate) (PMMA) (1 wt%‐doped films)

Compound	*Φ* ^a)^	*Φ*_F_ ^b)^	*Φ*_TADF_^b]^	*τ*_F_ ^c)^ [ns]	*τ*_TADF_ ^c)^ [µs]	*k*_F_ ^d)^ × 10^8^ [s^−1^]	*k*_IC_ ^d)^ × 10^7^ [s^−1^]	*k*_ISC_ ^d)^ × 10^7^ [s^−1^]	*k*_RISC_ ^d)^ × 10^5^ [s^−1^]
**V‐DABNA**	0.90	0.83	0.07	6.9	1.9	1.2	1.3	1.1	5.7
**V‐DABNA‐F**	0.81	0.74	0.07	6.6	1.7	1.1	2.7	1.4	6.5
**V‐DABNA‐Mes**	0.80	0.76	0.04	7.0	2.4	1.1	2.7	0.73	4.4

^a)^ Absolute photoluminescence quantum yield

^b)^ Fluorescent and TADF components determined from the total *Φ* and contribution of the integrated area of each component in the transient spectra to the total integrated area

^c)^ Lifetimes calculated from fluorescence decay

^d)^ Decay rates of fluorescence (*k*_F_), internal conversion from S_1_ to S_0_ (*k*_IC_), intersystem crossing from S_1_ to T_1_ (*k*_ISC_), and reverse intersystem crossing from T_1_ to S_1_ (*k*_RISC_) were calculated from *Φ*, *Φ*_F_, *Φ*_TADF_, *τ*_F_, and *τ*_TADF_ according to Adachi's method.

**Figure 3:**
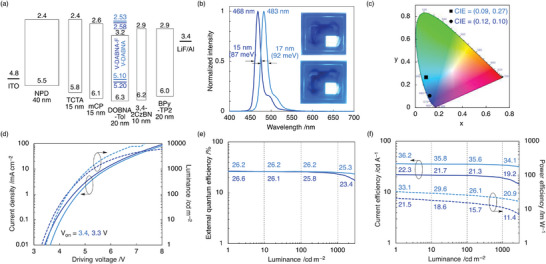
Characteristics of fabricated OLED devices using **V‐DABNA** (sky‐blue) and **V‐DABNA‐F** (blue) as emitters. a) Device structure, ionization potentials (*I*_p_), and electron affinities (*E*_a_) (in eV) for each component. The *I*_p_ and *E*_a_ of **V‐DABNA** and **V‐DABNA‐F** were estimated from those of **V‐DABNA‐Mes** and their HOMO/LUMO energy levels. b) Normalized EL spectra of the devices in operation. Inset: electroluminescence of the device. c) Commission Internationale d’Éclairage (CIE) (*x*,*y*) coordinates. d) Current density (solid) and luminance (dashed) versus driving voltage. e) External quantum efficiency (EQE) versus luminance. f) Current efficiency (solid) and power efficiency (dashed) versus luminance.

To demonstrate the potential of the proposed emitters, devices with the following structure were fabricated: indium tin oxide (ITO, 50 nm); *N,N*′‐di(1‐naphthyl)‐*N,N′*‐diphenyl‐(1,1′‐biphenyl)‐4,4′‐diamine (**NPD**, 40 nm); tris(4‐carbazolyl‐9‐ylphenyl)amine (**TCTA**, 15 nm); 1,3‐bis(*N*‐carbazolyl)benzene (**mCP**, 15 nm); 1 wt% **V‐DABNA** or **V‐DABNA‐F** emitter and 99 wt% **DOBNA‐Tol** ^[^ ^3g^ ^]^ (20 nm); 3,4‐di(9H‐carbazol‐9‐yl)benzonitrile (**3,4‐2CzBN**,^[^ ^11a^ ^]^ 10 nm); 2,7‐bis(2,2′‐bipyridine‐5‐yl)triphenylene (**BPy‐TP2**,^[^ ^11b^ ^]^ 20 nm); LiF (1 nm); and Al (100 nm). The electroluminescence characteristics, ionization potentials, and electron affinities of the fabricated devices are shown in **Figure** **3**. The **V‐DABNA**‐based device exhibited ultra‐narrowband blue emission at 483 nm with an FWHM of 17 nm (92 meV) and corresponding Commission Internationale d’Éclairage (CIE) coordinates of (0.09, 0.27). Notably, the fabricated device achieved excellent EQE of 26.2%, 26.2%, and 25.3% at 1, 100, and 1000 cd m^−2^, respectively, with the minimum efficiency roll‐off of 0.1% and 0.9% at 100 and 1000 cd m^−2^, respectively. These characteristics were significantly improved compared to the previously reported, solution‐processed OLED device employing **V‐DABNA‐Mes** as an emitter and are record‐setting for blue MR‐TADF materials (Table S5, Supporting Information). Thus, the decrease in molecular weight by replacing mesityl groups with phenyl groups enabled the fabrication of vacuum‐deposited OLED device, which improved the carrier balance and suppressed the efficiency‐roll off. The half‐lifetime (LT_50_) of the **V‐DABNA**‐based device was 184 h with an initial luminance of 500 cd m^−2^, which is longer than that of ***ν*‐DABNA** (2 h with an initial luminance at 500 cd m^−2^)^[^ ^12^ ^]^ due to the high *k*_RISC_ (Figure S10, Supporting Information). Furthermore, the **V‐DABNA‐F**‐based device exhibited ultrapure deep‐blue emission at 468 nm with an FWHM of 15 nm (87 meV), which is the smallest value for the reported blue MR‐TADF materials (Table S5, Supporting Information). The corresponding CIE coordinates are (0.12, 0.10), and the EQEs are 26.6%, 25.8%, and 23.4% at 1, 100, and 1000 cd m^−2^, respectively. The LT_50_ of the **V‐DABNA‐F**‐based device was 3.6 h with an initial luminance of 500 cd m^−2^, which is longer than that of ***ν*‐DABNA** but shorter than that of **V‐DABNA**. This is attributed to the electron trap caused by the larger electron affinity of **V‐DABNA‐F** (2.58 eV vs 2.53 eV, Figure 3a), which resulted in the C—N bond cleavage. Thus, the device lifetime can be improved by enhancing the electron affinity of host materials. In order to gain insight into high EQE, the angle dependence of the photoluminescence using codeposition films in **DOBNA‐Tol** were measured (Figure S11, Supporting Information). Notably, the orientational order parameters (*S*) of **V‐DABNA** and **V‐DABNA‐F** were determined to be −0.426 and −0.423, respectively, which are closer to the theoretical value (*S* = −0.5) for a perfectly horizontal orientation than that of ***ν*‐DABNA** (*S* = −0.407), resulting in high light outcoupling efficiency.

## 3 Conclusion

We synthesized ultra‐narrowband blue MR‐TADF materials (**V‐DABNA**, **V‐DABNA‐F**) by one‐shot triple borylation. The inductive effect of fluorine atoms effectively lowered the HOMO energy of **V‐DABNA,** which led to an increase in energy of the HOMO–LUMO gap. As a result, **V‐DABNA‐F** exhibited ultrapure deep‐blue TADF with a small FWHM of 16 nm, which is blueshifted from parent **V‐DABNA**. The OLED fabricated by vapor deposition using **V‐DABNA** as an emitter exhibited ultra‐narrowband blue emission at 483 nm with an FWHM of 17 nm, and achieved an excellent maximum EQE of 26.2%, with minimum efficiency roll‐offs of 0.1% and 0.9% at 100 and 1000 cd m^−2^, respectively, which are record‐setting values for blue MR‐TADF materials. In addition, the OLED fabricated using **V‐DABNA‐F** exhibited ultrapure deep‐blue emission at 468 nm with an FWHM of 15 nm, which is narrowest among the reported blue MR‐TADF materials. Thus, we have established a successful approach for the fabrication of ultra‐narrowband blue MR‐TADF emitters, which will pave the way for the development of highly efficient blue OLEDs.

## Conflict of Interest

The authors declare no conflict of interest.

## Supporting information

Supporting Information

## Data Availability

The data that support the findings of this study are available in the supplementary material of this article.
