# Supplementary material for: Ultra‐Narrowband Blue Multi‐Resonance Thermally Activated Delayed Fluorescence Materials
Source: Adv Sci (Weinh). 2022 Nov 17;10(1):2205070. doi: 10.1002/advs.202205070 (PMC9811429; doi:10.1002/advs.202205070)
Supplement: Supplementary file 1 — Supporting Information [file ADVS-10-2205070-s001.pdf]

Supporting Information

**Ultra-Narrowband Blue Multi-Resonance Thermally Activated Delayed Fluorescence Materials**

*Susumu Oda, Bungo Kawakami, Masaru Horiuchi, Yuki Yamasaki, Ryosuke Kawasumi, and Takuji Hatakeyama**

Dr. S. Oda, B, Kawakami, M. Horiuchi, Y. Yamasaki
Department of Chemistry, Graduate School of Science and Technology, Kwansei Gakuin University, 2-1 Gakuen, Sanda, Hyogo 669-1337, Japan.

R. Kawasumi
SK JNC Japan Co., Ltd. 25-1 Goi Kaigan, Ichihara, Chiba 290-8551, Japan.

Prof. T. Hatakeyama, M. Horiuchi
Department of Chemistry, Graduate School of Science, Kyoto University, Sakyo-ku, Kyoto 606-8502, Japan
E-mail: hatake@kuchem.kyoto-u.ac.jp

## EXPERIMENTAL SECTION

**General Procedure.** All the reactions dealing with air- or moisture-sensitive compounds were carried out in a dry reaction vessel (small scale, a Schlenk flask; large scale, a three-neched round bottomed flask) under a positive pressure of nitrogen. Air- and moisture-sensitive liquids and solutions were transferred *via* a syringe or a Teflon cannula. Analytical thin-layer chromatography (TLC) was performed on glass plates coated with 0.25 mm 230–400 mesh silica gel containing a fluorescent indicator (Merck, #1.05715.0009). TLC plates were visualized by exposure to ultraviolet light (254 nm or 365 nm) and/or by immersion in a basic staining solution of $KMnO_4$ followed by heating on a hot plate. Organic solutions were concentrated by rotary evaporation at *ca.* 10–50 mmHg. Flash column chromatography was performed on Merck silica gel 60 (spherical, neutral, 140–325 mesh) as described by Still et al.[1] Gel permeation chromatography was performed on a JAIGEL-1H and 2H (20 mm i.d.) with an LC-9130 (Japan Analytical Industry Co., Ltd.). Proton nuclear magnetic resonance ($^{1}$H NMR), carbon nuclear magnetic resonance ($^{13}$C NMR), boron nuclear magnetic resonance ($^{11}$B NMR), and fluorine nuclear magnetic resonance ($^{19}$F NMR) spectra were recorded on JEOL ECX400 (400 MHz) NMR spectrometers or JEOL ECX500 (500 MHz) or ECZ500R/S1 (500 MHz) NMR spectrometers. Proton chemical shift values are reported in parts per million (ppm, δ scale) downfield from tetramethylsilane and are referenced to the tetramethylsilane (δ 0), $(CDCl_2)_2$ (δ 6.00), or $CDCl_3$ (δ 7.26). $^{13}$C NMR spectra were recorded at 101 MHz or 126 MHz: carbon chemical shift values are reported in parts per million (ppm, δ scale) downfield from tetramethylsilane, and are referenced to the carbon resonance of tetramethylsilane (δ 0), $(CDCl_2)_2$ (δ 73.8), or $CDCl_3$ (δ 77.0). $^{11}$B NMR spectra were recorded at 128 MHz or 160 MHz: boron chemical shift values are reported in parts per million (ppm, δ scale) and are referenced to the external standard boron signal of $BF_3{\cdot}Et_2O$ (δ 0). $^{19}$F NMR spectra were recorded at 376 MHz or 470 MHz: fluorine chemical shift values are reported in parts per million (ppm, δ scale) and are referenced to the external standard fluorine signal of $CF_3COOH$ (δ –75.1). Data are presented as: chemical shift, multiplicity (s = singlet, d = doublet, t = triplet, q = quartet, quint = quintet, sext = sextet, sept = septet, m = multiplet and/or multiplet resonances, br = broad), coupling constant in hertz (Hz), signal area integration in natural numbers, and assignment (*italic*). IR spectra were recorded on an ATR-FTIR spectrometer (FT/IR-4200, JASCO or IRAffinity-1S, Shimadzu). Characteristic IR absorptions are reported in $cm^{-1}$. Melting points were recorded on a Fisher-Johns 12-144-1Q melting point apparatus (according to the limitations of the apparatus, the compounds which did not melt up to 300 °C are presented as ">300 °C" after confirming that is not decomposed using NMR). High-resolution mass spectra (HRMS) were obtained by the electron impact (EI) method with a JEOL JMS-T100GCv instrument, by the atmospheric pressure chemical

(1) Still, W. C.; Kahn, M.; Mitra, A. *J. Org. Chem*. **1978**, *43*, 2923–2925.

ionization (APCI) method with a BRUKER DALTONICS micrOTOF instrument, and by the electrospray ionization (ESI) method or the direct analysis in real time (DART) method with IonSense SVP100 and JEOL JMS-T100LP instruments. Low-resolution mass spectra (LRMS) were obtained by the electron impact (EI) method with a Shimadzu GCMS-QP2010 Ultra instrument. UV-visible absorption spectra were measured by a Shimazu UV-2600 instrument. Fluorescence spectra were measured by a HORIBA Scientific FluoroMax-4 instrument. Absolute PL quantum yields were recorded on a Hamamatsu Photonics Quantaurus-QY instrument. Fluorescence lifetimes were recorded on a Hamamatsu Photonics Quantaurus-Tau instrument. Purity of isolated compounds was determined by $^{1}$H NMR analyses, GC analysis on a Shimadzu GC-2025 instrument equipped with an FID detector and a capillary column (ZB-1MS, Phenomenex, 10 m × 0.10 mm i.d., 0.10 mm film thickness), or HPLC analysis on a JASCO UV-2070 Plus instrument equipped with a reversed-phase C18 column (InertSustain™ C18, GL Siences Inc., 4.6 mm × 250 mm i.d.).

**Materials.** Materials were purchased from Wako Pure Chemical Industries, Ltd. (Wako), Tokyo Chemical Industry Co., Ltd., Aldrich Inc., and other commercial suppliers, and were used after appropriate purification, unless otherwise noted. Florisil (100–200 mesh) was purchased from Kanto Chemical Co., Inc. (Kanto).

**Solvent.** Anhydrous solvents were purchased from above-described suppliers and/or dried over Molecular Sieves 4A and degassed before use. Water content of the solvent was determined with a Karl Fischer moisture titrator (AQ-2200, Hiranuma Sangyo Co., Ltd.) to be less than 20 ppm.

**Computational Method.** All calculations were performed with performed with Gaussian 09[2] or ADF2021[3] packages unless otherwise noted. The DFT method was employed using the B3LYP hybrid functional.[4] Structures were optimized with the 6-31G(d)[5] or TZP[6] basis set. The time-dependent density functional theory (TD-DFT) calculation[7] was conducted at the B3LYP/6-31G(d) level after the geometry optimization at the B3LYP/6-31G(d) level. Second-order algebraic diagrammatic construction (ADC(2)) calculations were performed using the TURBOMOL package[8] with def2-SVP basis set at the $T_1$ structures optimized at the (TD)B3LYP/TZP level.

---

(2) Gaussian 09, Revision C.01, Frisch, M. J.; Trucks, G. W.; Schlegel, H. B.; Scuseria, G. E.; Robb, M. A.; Cheeseman, J. R.; Scalmani, G.; Barone, V.; Mennucci, B.; Petersson, G. A.; Nakatsuji, H.; Caricato, M.; Li, X.; Hratchian, H. P.; Izmaylov, A. F.; Bloino, J.; Zheng, G.; Sonnenberg, J. L.; Hada, M.; Ehara, M.; Toyota, K.; Fukuda, R.; Hasegawa, J.; Ishida, M.; Nakajima, T.; Honda, Y.; Kitao, O.; Nakai, H.; Vreven, T.; Montgomery, Jr., J. A.; Peralta, J. E.; Ogliaro, F.; Bearpark, M.; Heyd, J. J.; Brothers, E.; Kudin, K. N.; Staroverov, V. N.; Keith, R.; Kobayashi, R.; Normand, J.; Raghavachari, K.; Rendell, A.; Burant, J. C.; Iyengar, S. S.; Tomasi, J.; Cossi, M.; Rega, N.; Millam, N. J.; Klene, M.; Knox, J. E.; Cross, J. B.; Bakken, V.; Adamo, C.; Jaramillo, J.; Gomperts, R.; Stratmann, R. E.; Yazyev, O.; Austin, A. J.; Cammi, R.; Pomelli, C.; Ochterski, J. W.; Martin, R. L.; Morokuma, K.; Zakrzewski, V. G.; Voth, G. A.; Salvador, P.; Dannenberg, J. J.; Dapprich, S.; Daniels, A. D.; Farkas, Ö.; Foresman, J. B.; Ortiz, J. V.; Cioslowski, J.; Fox, D. J. Gaussian, Inc., Wallingford CT, 2010.

(3) ADF2021, SCM, Theoretical Chemistry, Vrije Universiteit, Amsterdam, The Netherlands, http://www.scm.com/

(4) (a) Becke, A. D. *J. Chem. Phys.* **1993**, *98*, 5648–5652. (b) Lee, C.; Yang, W.; Parr, R. G. *Phys. Rev. B* **1988**, *37*, 785.

(5) Hehre, W. J.; Radom, L.; Schleyer, P. v. R.; Pople, J. A. *Ab Initio Molecular Orbital Theory*; John Wiley & Sons: New York, 1986 and references cited therein.

(6) Van Lenthe, E.; Baerends, E. J. *J. Comput. Chem.* **2003**, *24*, 1142–1156.

(7) (a) Casida, M. E.; Jamorski, C.; Casida, K. C.; Salahub, D. R. *J. Chem. Phys.* **1998**, *108*, 4439. (b) Stratmann, R. E.; Scuseria, G. E.; Frisch, M. J. *J. Chem. Phys.* **1998**, *109*, 8218.

(8) TURBOMOLE V7.4 2019, a development of University of Karlsruhe and Forschungszentrum Karlsruhe GmbH, 1989-2007, TURBOMOLE GmbH, since 2007; available from http://www.turbomole.com.

**Table S1.** Summary of TD-DFT Calculation for **V-DABNA**, **V-DABNA-F**, **V-DABNA-Mes** at the B3LYP/6-31G(d) Level of Theory (Gaussian 09).

| compound | HOMO–1 [eV] | HOMO [eV] | LUMO [eV] | LUMO+1 [eV] | $\lambda_{(S0\text{-}S1)}$ [nm] | $E_S$[a] [eV] | $f$[b] | $\lambda_{(S0\text{-}T1)}$ [nm] | $E_T$[c] [eV] | $\Delta E_{ST}$[d] [eV] |
|---|---|---|---|---|---|---|---|---|---|---|
| **V-DABNA** | –4.61 | –4.45 | –1.18 | –0.87 | 450 | 2.75 | 0.3933 | 491 | 2.53 | 0.22 |
| **V-DABNA-F** | –4.69 | –4.55 | –1.23 | –0.92 | 442 | 2.81 | 0.3828 | 480 | 2.58 | 0.23 |
| **V-DABNA-Mes** | –4.64 | –4.47 | –1.23 | –0.91 | 454 | 2.73 | 0.3929 | 496 | 2.50 | 0.23 |

[a]Singlet–singlet excitation energy. [b]Oscillator strength. [c]Singlet–triplet excitation energy. [d]Energy gap between $S_1$ and $T_1$ states.

| compound | $S_1/T_1/T_2$ energy | coefficient of orbital | | |
|---|---|---|---|---|
| **V-DABNA** | $S_1$: 2.75 eV (450 nm) | HOMO–1 → LUMO+1 (0.10328) | HOMO → LUMO (0.68751) | |
| | $T_1$: 2.53 eV (491 nm) | HOMO–1 → LUMO+1 (0.17533) | HOMO → LUMO (0.65379) | |
| | $T_2$: 2.64 eV (469 nm) | HOMO–2 → LUMO+1 (0.18933) | HOMO–1 → LUMO (0.54720) | HOMO → LUMO+1 (0.33139) |
| **V-DABNA-F** | $S_1$: 2.81 eV (442 nm) | HOMO–1 → LUMO+1 (0.10894) | HOMO → LUMO (0.68301) | |
| | $T_1$: 2.58 eV (480 nm) | HOMO–4 → LUMO+1 (–0.10070) | HOMO–1 → LUMO+1 (0.19581) | HOMO → LUMO (0.64119) |
| | $T_2$: 2.68 eV (463 nm) | HOMO–2 → LUMO+1 (0.14634) | HOMO–1 → LUMO (0.54139) | HOMO → LUMO+1 (0.35178) |
| **V-DABNA-Mes** | $S_1$: 2.73 eV (454 nm) | HOMO → LUMO (0.68908) | | |
| | $T_1$: 2.50 eV (496 nm) | HOMO–1 → LUMO+1 (–0.16795) | HOMO → LUMO (0.65785) | |
| | $T_2$: 2.63 eV (471 nm) | HOMO–2 → LUMO+1 (–0.17899) | HOMO–1 → LUMO (0.54432) | HOMO → LUMO+1 (–0.34396) |

**Table S2.** Summary of Photophysical Data of **V-DABNA**, **V-DABNA-F**, **V-DABNA-Mes** in PMMA Films (1 wt%, excited at 340 nm).

| compound | $\lambda_{ab}$[a] [nm] | $\lambda_{em}$[b] [nm] | FWHM[c] [nm] | $\Phi$[d] | $\Phi_F$[e] | $\Phi_{TADF}$[f] | $\tau_F$[g] [ns] | $\tau_{TADF}$[h] [μs] | $k_F$[i] [$10^8$ s$^{-1}$] | $k_{IC}$[i] [$10^7$ s$^{-1}$] | $k_{ISC}$[i] [$10^7$ s$^{-1}$] | $k_{RISC}$[i] [$10^5$ s$^{-1}$] |
|---|---|---|---|---|---|---|---|---|---|---|---|---|
| **V-DABNA** | 473 | 481 | 17 | 0.90 | 0.83 | 0.07 | 6.9 | 1.9 | 1.2 | 1.3 | 1.1 | 5.7 |
| **V-DABNA-F** | 458 | 464 | 16 | 0.81 | 0.74 | 0.07 | 6.6 | 1.7 | 1.1 | 2.7 | 1.4 | 6.5 |
| **V-DABNA-Mes** | 477 | 484 | 16 | 0.80 | 0.76 | 0.04 | 7.0 | 2.4 | 1.1 | 2.7 | 0.73 | 4.4 |

[a]Maximum wavelength of absorption. [b]Maximum wavelength of fluorescence. [c]Full width at half maximum. [d]Absolute photoluminescence quantum yield. [e]Quantum yield of fluorescent (prompt) component. [f]Quantum yield of TADF (delay) component. [g]Lifetime of fluorescent (prompt) component. [h]Lifetime of TADF (delay) component. [i]$k_F$, $k_{IC}$, $k_{ISC}$, $k_{RISC}$ are rate constants for fluorescence, internal conversion, intersystem crossing and reverse intersystem crossing, respectively.

**Table S3.** Summary of Photophysical Data of **V-DABNA**, **V-DABNA-F**, **V-DABNA-Mes** in Toluene ($1.0 \times 10^{-5}$ M, excited at 365 nm).

| compound | $\lambda_{ab}$[a] [nm] | $\varepsilon$[b] | $\lambda_{em}$[c] [nm] | FWHM[d] [nm] | $\Phi$[e] | $\Phi_F$[f] | $\Phi_{TADF}$[g] | $\tau_F$[h] [ns] | $\tau_{TADF}$[i] [μs] | $k_F$[j] [$10^8$ s$^{-1}$] | $k_{IC}$[j] [$10^7$ s$^{-1}$] | $k_{ISC}$[j] [$10^7$ s$^{-1}$] | $k_{RISC}$[j] [$10^5$ s$^{-1}$] |
|---|---|---|---|---|---|---|---|---|---|---|---|---|---|
| **V-DABNA** | 473 | 4.8 | 483 | 14 | 0.85 | 0.80 | 0.05 | 6.3 | 1.5 | 1.3 | 2.2 | 0.96 | 7.1 |
| **V-DABNA-F** | 459 | 4.6 | 467 | 13 | 0.91 | 0.85 | 0.06 | 6.3 | 2.1 | 1.3 | 1.4 | 1.0 | 5.1 |
| **V-DABNA-Mes** | 476 | 3.7 | 486 | 13 | 0.79 | 0.76 | 0.03 | 6.7 | 2.5 | 1.1 | 3.0 | 0.63 | 4.2 |

[a]Maximum wavelength of absorption. [b]Molar absorbance coefficient [$10^4$ L mol$^{-1}$ cm$^{-1}$]. [c]Maximum wavelength of fluorescence. [d]Full width at half maximum. [e]Absolute photoluminescence quantum yield. [f]Quantum yield of fluorescent (prompt) component. [g]Quantum yield of TADF (delay) component. [h]Lifetime of fluorescent (prompt) component. [i]Lifetime of TADF (delay) component. [j]$k_F$, $k_{IC}$, $k_{ISC}$, $k_{RISC}$ are rate constants for fluorescence, internal conversion, intersystem crossing and reverse intersystem crossing, respectively.

**Synthesis of *N*[1],*N*[1],*N*[3],*N*[5]-tetraphenylbenzene-1,3,5-triamine (2)**

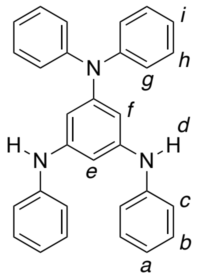


Aniline (2.71 mL, 30 mmol) was added to a mixture of 3,5-dichloro-*N,N*-diphenylaniline (3.14 g, 10 mmol), sodium *tert*-butoxide (4.81 g, 50 mmol), SPhos (0.246 g, 0.60 mmol), tris(dibenzylideneacetone)dipalladium(0) (0.275 g, 0.30 mmol) in toluene (100 mL) under a nitrogen atmosphere. After stirring at 80 ºC for 9 h, the reaction mixture was allowed to cool to room temperature. After addition of aqueous 1 *N* hydrochloric acid (30 mL), the aqueous layer was separated, basified by saturated sodium carbonate aqueous solution (50 mL) and extracted with dichloromethane (40 mL, three times). After the solvent was removed *in vacuo*, the yield of the title compound in the crude product was determined to be 90% yield by $^1$H NMR analysis using 1,1,2,2-tetrachloroethane as an internal standard. The crude product was purified by silica gel column chromatography (eluent: hexane/ethyl acetate = 5/1) to obtain the title compound (3.21 g, 75% yield, 99% pure on NMR analysis) as a white solid. IR (neat): cm$^{-1}$ 3391 (N–H), 3036 (Ar–H), 1609, 1580, 1516, 1493, 1464, 1396, 1294, 1231, 1171, 1153, 1074, 1032, 849, 754, 733, 691, 656; mp: 137.0–137.5 ºC, $^1$H NMR ($CDCl_3$, 400 MHz) δ 5.47 (s, 2H, *d*), 6.27 (d, $J$ = 1.6 Hz, 2H, *f*), 6.41 (t, $J$ = 1.6 Hz, 1H, *e*), 6.84 (t, $J$ = 7.4 Hz, 2H, *a*), 6.93–7.00 (m, 6H, *c*, *i*), 7.10–7.18 (m, 8H, *b*, *g*), 7.22 (t, $J$ = 7.8 Hz, 4H, *h*); $^{13}$C NMR ($CDCl_3$, 101 MHz) δ 100.4 (1C), 105.9 (2C), 118.0 (4C), 120.9 (2C), 122.8 (2C), 124.6 (4C), 129.1 (4C), 129.2 (4C), 142.7 (2C), 144.6 (2C), 147.6 (2C), 149.8 (1C); HRMS (MALDI-TOF/MS) *m/z* [M]$^+$ calcd for $C_{30}H_{25}N_3$ 427.2043, observed 427.2046.

**Synthesis of *N*[1],*N*[3]-bis(3-chlorophenyl)-*N*[1],*N*[3],*N*[5],*N*[5]-tetraphenylbenzene-1,3,5-triamine (3)**

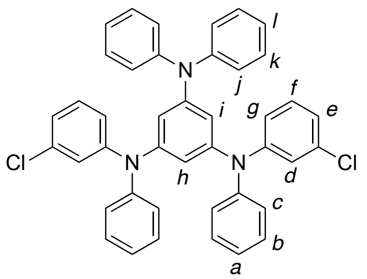


3-Chloroiodobenzene (0.855 mL, 6.9 mmol) was added to a mixture of **2** (1.48 g, 3.5 mmol), sodium *tert*-butoxide (1.00 g, 10 mmol), tri-*tert*-butylphosphonium tetrafluoroborate (41.1 mg, 0.14 mmol), tris(dibenzylideneacetone)dipalladium(0) (64.5 mg, 0.070 mmol) in toluene (14 mL) under a nitrogen atmosphere. After stirring at 60 ºC for 12 h, the reaction mixture was allowed to cool to

room temperature. After addition of aqueous 1 *N* hydrochloric acid (15 mL), the aqueous layer was separated, basified by saturated sodium carbonate aqueous solution (25 mL) and extracted with dichloromethane (20 mL, three times). After the solvent was removed *in vacuo*, the yield of the title compound in the crude product was determined to be 97% yield by $^{1}$H NMR analysis using 1,1,2,2-tetrachloroethane as an internal standard. The crude product was purified by silica gel column chromatography (eluent: hexane/ethyl acetate = 10/1) to obtain the title compound (2.02 g, 90% yield, 99% pure on NMR analysis) as a white solid. IR (neat): cm$^{-1}$ 3069, 3032 (Ar–H), 2367, 2361, 2353, 2344, 2334, 2320, 1574, 1568, 1558, 1489, 1472, 1462, 1287, 1242, 1175, 1150, 1074, 1045, 995, 947, 866, 843, 775, 754, 689, 633; mp: 170.5–171.0 ºC, $^{1}$H NMR ($CDCl_3$, 400 MHz) δ 6.34 (t, *J* = 2.0 Hz, 1H, *h*), 6.41 (d, *J* = 2.0 Hz, 2H, *i*), 6.84–6.87 (m, 4H, *e*, *g*), 6.92–7.07 (m, 16H, *a*, *c*, *d*, *f*, *j*, *l*), 7.16–7.23 (m, 8H, *b*, *k*); $^{13}$C NMR ($CDCl_3$, 101 MHz) δ 114.3 (1C), 114.4 (2C), 120.9 (2C), 122.0 (2C), 122.7 (2C), 122.9 (2C), 123.6 (2C), 124.2 (4C), 124.7 (4C), 129.1 (4C), 129.3 (4C), 129.9 (2C), 134.5 (2C), 146.5 (2C), 147.0 (2C), 148.4 (2C), 148.5 (2C), 149.3 (1C); HRMS (MALDI-TOF/MS) *m/z* $[M]^+$ calcd for $C_{42}H_{31}Cl_2N_3$ 647.1890, observed 647.1894.

**Synthesis of *N*$^{1}$,*N*$^{1}$,*N*$^{3}$,*N*$^{5}$-tetraphenyl-*N*$^{3}$,*N*$^{5}$-bis(3-(phenylamino)phenyl)benzene-1,3,5-triamine (4)**

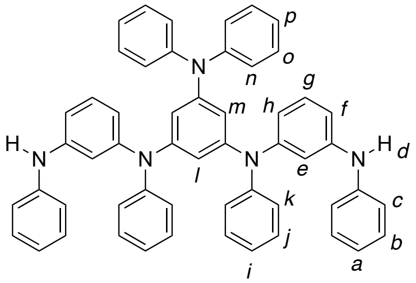


Aniline (0.822 mL, 9.0 mmol) was added to a mixture of **3** (1.95 g, 3.0 mmol), sodium *tert*-butoxide (1.44 g, 15 mmol), SPhos (73.9 mg, 0.18 mmol), tris(dibenzylideneacetone)dipalladium(0) (82.4 mg, 0.090 mmol) in toluene (15 mL) under a nitrogen atmosphere. After stirring at 100 ºC for 12 h, the reaction mixture was allowed to cool to room temperature. After addition of aqueous 1 *N* hydrochloric acid (10 mL), the aqueous layer was separated, basified by saturated sodium carbonate aqueous solution (30 mL) and extracted with dichloromethane (60 mL, three times). After the solvent was removed *in vacuo*, the crude product was purified by silica gel column chromatography (eluent: hexane/ethyl acetate = 5/1) to obtain the title compound (1.69 g, 74% yield, 99% pure on NMR analysis) as a white solid. IR (neat): cm$^{-1}$ 3393 (N–H), 3034 (Ar–H), 1582, 1570, 1487, 1287, 1242, 1200, 1163, 1074, 1028, 995, 868, 748, 689; mp: 217.0–219.5 ºC, $^{1}$H NMR ($CDCl_3$, 400 MHz) δ 5.54 (s, 2H, *d*), 6.46–6.47 (m, 3H, *l*, *m*), 6.57 (d, *J* = 8.0 Hz, 2H, *h*), 6.63 (d, *J* = 8.4 Hz, 2H, *f*), 6.72 (s, 2H, *e*), 6.86–7.06 (m, 20H, *a*, *c*, *g*, *i*, *k*, *n*, *p*), 7.13–7.23 (m, 12H, *b*, *j*, *o*); $^{13}$C NMR ($CDCl_3$, 101 MHz) δ 111.9 (2C), 113.1 (2C), 114.8

(2C), 114.9 (1C), 116.4 (2C), 117.6 (4C), 120.8 (2C), 122.5 (2C), 122.7 (2C), 123.9 (4C), 124.1 (4C), 129.0 (8C), 129.3 (4C), 129.7 (2C), 143.0 (2C), 143.6 (2C), 147.1 (2C), 147.3 (2C), 148.3 (2C), 148.8 (2C), 148.9 (1C); HRMS (MALDI-TOF/MS) *m/z* $[M]^+$ calcd for $C_{54}H_{43}N_5$ 761.3513, observed 761.3514.

**Synthesis of *N*¹,*N*¹'-(((5-diphenylamino)-1,3-phenylene)bis(phenylazanediyl))bis (3,1-phenylene))bis(*N*¹,*N*³,*N*³,*N*⁵,*N*⁵-pentaphenylbenzene-1,3,5-triamine (5)**

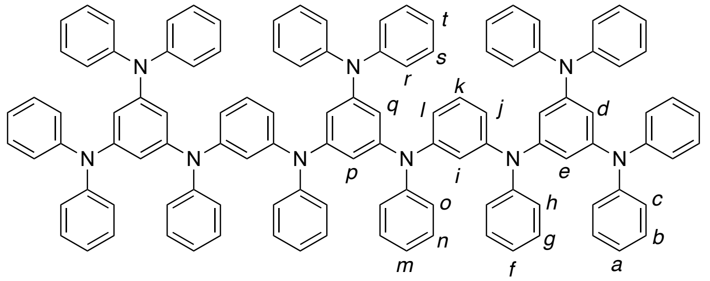


Compound **4** (0.724 g, 0.95 mmol), 5-chloro-*N*¹,*N*¹,*N*³,*N*³-tetraphenylbenzene-1,3-diamine (1.02 g, 2.3 mmol), tris(dibenzylideneacetone)dipalladium(0) (65.3 mg, 0.071 mmol), SPhos (58.5 mg, 0.14 mmol) and sodium *tert*-butoxide (0.274 g, 2.9 mmol) were dissolved in toluene (9.4 mL) under a nitrogen atmosphere. After stirring at 110 ºC for 12 h, the reaction mixture was allowed to cool to room temperature. After addition of aqueous 1 *N* hydrochloric acid (10 mL), the aqueous layer was separated, basified by saturated sodium carbonate aqueous solution (20 mL) and extracted with dichloromethane (30 mL, three times). After the solvent was removed *in vacuo*, the yield of the title compound in the crude product was determined to be 92% yield by $^1$H NMR analysis using 1,1,2,2-tetrachloroethane as an internal standard. The crude product was purified by silica gel column chromatography (eluent: hexane/ethyl acetate = 10/1, 5/1) to obtain the title compound (1.23 g, 83% yield, 97% pure on NMR analysis) as a white solid. IR (neat): cm$^{-1}$ 3032, 2924 (Ar–H), 1717, 1585, 1495, 1292, 1240, 752, 692; mp: >300 ºC, $^1$H NMR ($CDCl_3$, 400 MHz) δ 6.37 (m, 7H, *e*, *p*, *q*), 6.40 (s, 2H, *d*), 6.56 (m, 4H, *j*, *l*), 6.69 (s, 2H, *i*), 6.80–7.00 (m, 44H, *a*, *c*, *f*, *h*, *k*, *m*, *o*, *r*, *t*), 7.05–7.14 (m, 28H, *b*, *g*, *n*, *s*); $^{13}$C NMR ($CDCl_3$, 101 MHz) δ 114.3 (2C), 114.5 (4C), 114.7 (2C), 114.9 (1C), 118.4 (4C), 119.4 (2C), 122.2 (2C), 122.3 (2C), 122.6 (8C), 123.3 (4C), 123.4 (4C), 123.9 (24C), 124.2 (2C), 128.8 (4C), 128.9 (4C), 129.0 (24C), 129.4 (2C), 147.0 (4C), 147.1 (8C), 147.9 (2C), 148.6 (2C), 148.9 (3C); HRMS (MALDI-TOF/MS) *m/z* $[M+H]^+$ calcd for $C_{114}H_{88}N_9$ 1582.7111, observed 1582.7104.

**Synthesis of *N*7,*N*7,*N*13,*N*13,*N*19,*N*19,5,9,11,15,17,21-dodecaphenyl-5,9,11,15,17,21-hexahydro-5,9,11,15,17,21-hexaaza-25b,26b,27b-triboranaphtho[3,2,1-*de*] naphtho[3',2',1':10,11]tetraceno[1,2,3-*jk*]pentacene-7,13,19-triamine (V-DABNA)**

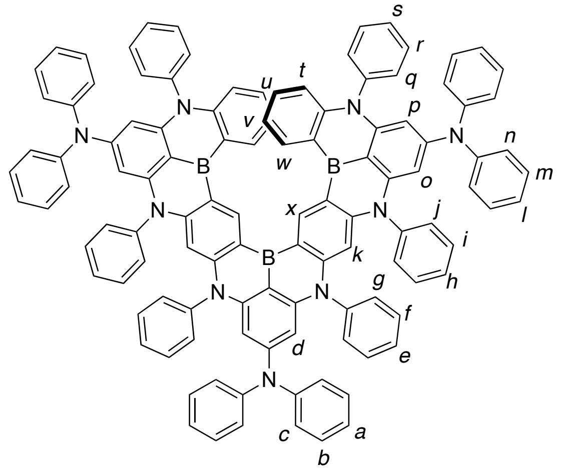

Boron tribromide (0.129 mL, 1.4 mmol) was added to a solution of **1** (0.135 g, 0.085 mmol) in 2,4-dichlorotoluene (1.3 mL) under a nitrogen atmosphere. After stirring at 200 ºC for 18 h, the reaction mixture was allowed to cool to room temperature and hydrogen bromide was removed *in vacuo*. After phosphorus buffer solution (pH = 7, 5.0 mL) was added to the reaction mixture at 0 ºC, the aqueous layer was separated and extracted with dichloromethane (100 mL, three times). After the solvent was removed *in vacuo*, the crude product was purified by silica gel column chromatography (eluent: hexane/dichloromethane = 3/2) to obtain the title compound (15.1 mg, 11% yield, 91% pure on NMR analysis) as a yellow solid. IR (neat): $cm^{-1}$ 3057, 2926 (Ar–H), 1576, 1489, 1427, 1292, 1260, 1163, 1070, 1024, 822, 752, 692; mp: >300 ºC, $^{1}$H NMR ($CDCl_3$, 400 MHz) δ 5.64–5.65 (s, 6H, *d*, *o*, *p*), 5.74 (s, 2H, *k*), 6.35 (t, *J* = 7.4 Hz, 2H, *v*), 6.48 (d, *J* = 8.8 Hz, 2H, *t*), 6.85–6.93 (m, 20H, *a*, *c*, *l*, *n*, *u*), 7.00–7.07 (m, 20H, *b*, *g*, *j*, *m*), 7.10–7.26 (m, 16H, *e*, *f*, *h*, *i*, *q*), 7.33 (t, *J* = 7.6 Hz, 2H, *s*), 7.45 (t, *J* = 7.6 Hz, 4H, *r*), 9.00 (d, *J* = 6.8 Hz, 2H, *w*), 10.6 (s, 2H, *x*); $^{11}$B NMR (160 MHz, $C_6D_4Cl_2$) δ 34.7; HRMS (MALDI-TOF/MS) *m/z* $[M]^+$ calcd for $C_{114}H_{78}B_3N_9$ 1605.6695, observed 1605.6753. $^{13}$C NMR peaks were barely detected because of the low solubility of **V-DABNA**.

**Synthesis of *N*[1],*N*[3]-bis(2,6-difluorophenyl)-*N*[5],*N*[5]-diphenylbenzene-1,3,5-triamine (2-F)**

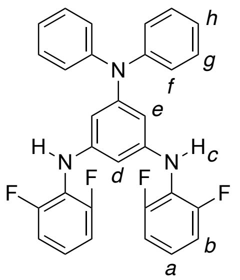


2,6-Difluoroaniline (22.7 mL, 0.23 mol) was added to a mixture of 3,5-dichloro-*N,N*-diphenylaniline (23.6 g, 75 mmol), sodium *tert*-butoxide (36.0 g, 0.38 mol), SPhos (0.616 g, 1.5 mmol), tris(dibenzylideneacetone)dipalladium(0) (0.687 g, 0.75 mmol) in toluene (750 mL) under a nitrogen atmosphere. After stirring at 100 ºC for 8 h, the reaction mixture was allowed to cool to room temperature. After addition of aqueous 1 *N* hydrochloric acid (60 mL), the aqueous layer was separated, basified by saturated sodium carbonate aqueous solution (100 mL) and extracted with toluenee (100 mL, three times). After the solvent was removed *in vacuo*, the crude product was purified by silica gel column chromatography (eluent: hexane/ethyl acetate = 6/1) to obtain the title compound (27.0 g, 72% yield, 97% pure on NMR analysis) as a white solid. IR (neat): $cm^{-1}$ 3443 (N–H), 3051 (Ar–H), 1587, 1493, 1464, 1290, 1273, 1233, 1165, 1030, 989, 773, 748, 696; mp: 142.5–144.0 ºC, $^{1}H$ NMR ($CDCl_3$, 400 MHz) δ 5.30 (s, 2H, *c*), 5.87 (s, 1H, *d*), 6.06 (s, 2H, *e*), 6.81–7.00 (m, 8H, *a*, *b*, *h*), 7.11 (d, $J$ = 8.0 Hz, 4H, *f*), 7.22 (t, $J$ = 8.4 Hz, 4H, *g*); $^{13}C$ NMR ($CDCl_3$, 101 MHz) δ 97.9 (1C), 104.1 (2C), 111.6–111.8 (4C), 119.1 (2C), 122.7 (2C), 123.2 (2C), 124.7 (4C), 129.0 (4C), 145.0 (2C), 147.4 (2C), 149.1 (1C), 155.4–157.9 (4C); $^{19}F$ NMR ($CDCl_3$, 376 MHz) δ –119.9; HRMS (MALDI-TOF/MS) *m/z* $[M]^+$ calcd for $C_{30}H_{21}F_4N_3$ 499.1666, observed 499.1667.

**Synthesis of *N*[1],*N*[3]-bis(3-chlorophenyl)-*N*[1],*N*[3]-bis(2,6-difluorophenyl)-*N*[5],*N*[5]-diphenylbenzene -1,3,5-triamine (3-F)**

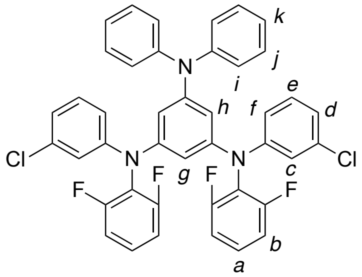


3-Chloroiodobenzene (7.41 mL, 60 mmol) was added to a mixture of **2-F** (15.0 g, 30 mmol), sodium *tert*-butoxide (8.65 g, 90 mmol), tri-*tert*-butylphosphonium tetrafluoroborate (0.348 g, 1.2 mmol), tris(dibenzylideneacetone)dipalladium(0) (0.559 g, 0.61 mmol) in *tert*-butylbenzene (200 mL) under a nitrogen atmosphere. After stirring at 140 ºC for 12 h, the reaction mixture was

allowed to cool to room temperature. After addition of aqueous 1 *N* hydrochloric acid (40 mL), the aqueous layer was separated, basified by saturated sodium carbonate aqueous solution (80 mL) and extracted with toluene (50 mL, three times). After the solvent was removed *in vacuo*, the crude product was purified by silica gel column chromatography (eluent: hexane/dichloromethane = 2/1) to obtain the title compound (13.6 g, 63% yield, 97% pure on NMR analysis) as a yellow solid. IR (neat): $cm^{-1}$ 3073 (Ar–H), 1584, 1493, 1464, 1292, 1238, 1148, 1001, 779, 752, 692; mp: 183.0–185.0 °C, $^{1}$H NMR ($CDCl_3$, 400 MHz) δ 6.28 (s, 1H, *g*), 6.37 (s, 2H, *h*), 6.77 (d, *J* = 8.4 Hz, 2H, *f*), 6.84–6.96 (m, 10H, *a*, *b*, *c*, *d*), 7.03–7.07 (m, 6H, *i*, *k*), 7.12–7.20 (m, 6H, *e*, *j*); $^{13}$C NMR ($CDCl_3$, 101 MHz) δ 109.1 (1C), 111.6 (2C), 112.4–112.7 (4C), 118.1 (2C), 120.0 (2C), 121.7 (2C), 122.0 (2C), 123.0 (2C), 124.3 (4C), 127.4 (2C), 129.1 (4C), 129.9 (2C), 134.6 (2C), 146.8 (2C), 147.0 (2C), 147.1 (2C), 149.3 (1C), 158.7–161.3 (4C); $^{19}$F NMR ($CDCl_3$, 376 MHz) δ –116.7; HRMS (MALDI-TOF/MS) *m/z* $[M]^+$ calcd for $C_{42}H_{27}Cl_2F_4N_3$ 719.1513, observed 719.1508.

**Synthesis of *N*¹,*N*³-bis(2,6-difluorophenyl)-*N*¹,*N*³-bis(3-((2,6-difluorophenyl)amino)phenyl) -*N*⁵,*N*⁵-diphenylbenzene-1,3,5-triamine (4-F)**

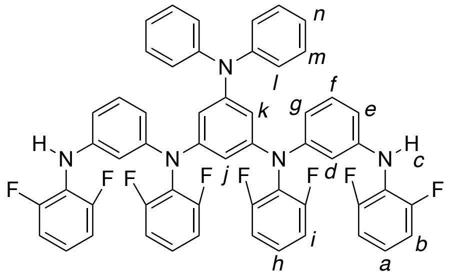


2,6-Difluoroaniline (4.54 mL, 45 mmol) was added to a mixture of **3-F** (10.8 g, 15 mmol), sodium *tert*-butoxide (7.21 g, 75 mmol), SPhos (0.246 g, 0.60 mmol), tris(dibenzylideneacetone)dipalladium(0) (0.275 g, 0.30 mmol) in toluene (150 mL) under a nitrogen atmosphere. After stirring at 100 °C for 12 h, the reaction mixture was allowed to cool to room temperature. After addition of aqueous 1 *N* hydrochloric acid (20 mL), the aqueous layer was separated, basified by saturated sodium carbonate aqueous solution (40 mL) and extracted with dichloromethane (80 mL, three times). After the solvent was removed *in vacuo*, the crude product was purified by silica gel column chromatography (eluent: hexane/dichloromethane = 1/1) to obtain the title compound (7.16 g, 51% yield, 97% pure on NMR analysis) as a white solid. IR (neat): $cm^{-1}$ 3412 (N–H), 3067 (Ar–H), 1584, 1495, 1470, 1292, 1238, 1003, 779, 754, 689; mp: 195.5–200.0 °C, $^{1}$H NMR ($CDCl_3$, 400 MHz) δ 5.31 (s, 2H, *c*), 6.30 (d, *J* = 2.0 Hz, 2H, *k*), 6.34 (t, *J* = 2.0 Hz, 1H, *j*), 6.40–6.45 (m, 6H, *d*, *e*, *g*), 6.81–7.16 (m, 24H, *a*, *b*, *f*, *h*, *i*, *l*, *m*, *n*); $^{13}$C NMR ($CDCl_3$, 101 MHz) δ 108.4 (2C), 109.2 (1C), 109.6 (2C), 111.7–111.9 (6C), 112.2–112.4 (4C), 113.0 (2C), 119.2 (2C), 122.4–122.5 (4C), 122.9 (2C), 123.9 (4C), 126.8 (2C), 128.9 (4C), 129.3 (2C), 144.0 (2C), 146.6 (2C), 147.2 (2C), 147.3 (2C), 148.8 (1C), 155.1–157.7 (4C), 158.9–161.4 (4C); $^{19}$F NMR ($CDCl_3$,

376 MHz) δ –116.5, –119.7; HRMS (MALDI-TOF/MS) *m/z* $[M]^+$ calcd for $C_{54}H_{35}F_8N_5$ 905.2759, observed 905.2740.

**Synthesis of *N*[1],*N*[1']-(((5-(diphenylamino)-1,3-phenylene)bis((2,6-difluorophenyl)azanediyl)) bis(3,1-phenylene))bis(*N*[1]-(2,6-difluorophenyl)-*N*[3],*N*[3],*N*[5],*N*[5]-tetraphenylbenzene-1,3,5-triamine ) (5-F)**

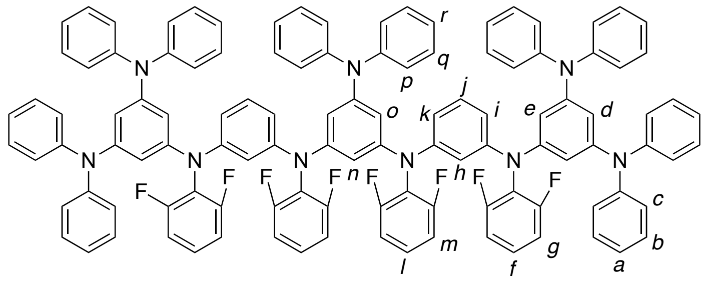


Compound **4-F** (1.63 g, 1.8 mmol), 5-chloro-*N*[1],*N*[1],*N*[3],*N*[3]-tetraphenylbenzene-1,3-diamine (2.01 g, 4.5 mmol), tris(dibenzylideneacetone)dipalladium(0) (82.4 mg, 0.090 mmol), SPhos (73.9 mg, 0.18 mmol) and sodium *tert*-butoxide (0.519 g, 5.4 mmol) were dissolved in *tert*-butylbenzene (20 mL) under a nitrogen atmosphere. After stirring at 140 ºC for 18 h, the reaction mixture was allowed to cool to room temperature. After addition of aqueous 1 *N* hydrochloric acid (5 mL), the aqueous layer was separated, basified by saturated sodium carbonate aqueous solution (10 mL) and extracted with dichloromethane (100 mL, three times). After the solvent was removed *in vacuo*, the crude product was purified by silica gel column chromatography (eluent: hexane/dichloromethane = 2/1) to obtain the title compound (2.67 g, 86% yield, 97% pure on NMR analysis) as a white solid. IR (neat): $cm^{-1}$ 3036 (Ar–H), 1584, 1574, 1495, 1474, 1288, 1242, 1007, 783, 752, 692; mp: 272.0–274.0 ºC $^1H$ NMR ($CDCl_3$, 400 MHz) δ 6.14 (s, 1H, *n*), 6.26–6.28 (m, 6H, *e*, *o*), 6.37–6.39 (m, 4H, *d*, *h*), 6.48–6.50 (m, 4H, *i*, *k*), 6.70–6.75 (m, 8H, *g*, *m*), 6.83–6.89 (m, 12H, *a*, *j*, *r*), 6.93–6.99 (m, 24H, *c*, *f*, *l*, *p*), 7.08–7.13 (m, 20H, *b*, *q*); $^{13}C$ NMR ($CDCl_3$, 101 MHz) δ 108.7 (1C), 111.2 (2C), 111.5 (4C), 112.1–112.3 (8C), 113.0 (2C), 114.1 (2C), 114.5 (2C), 114.6 (2C), 122.2–122.5 (14C), 123.9 (20C), 126.4 (4C), 128.9 (22C), 146.4 (6C), 147.1 (2C), 147.2 (10C), 148.7 (1C), 148.8 (4C), 158.5–161.1 (8C); $^{19}F$ NMR ($CDCl_3$, 376 MHz) δ –116.2, –116.4; HRMS (MALDI-TOF/MS) *m/z* $[M+H]^+$ calcd for $C_{114}H_{80}F_8N_9$ 1727.6436, observed 1727.6458.

**Synthesis of 9,11,15,17-tetrakis(2,6-difluorophenyl)-*N*[7],*N*[7],*N*[13],*N*[13],*N*[19],*N*[19],5,21-octaphenyl-5,9,11,15,17,21-hexahydro-5,9,11,15,17,21-hexaaza-25b,26b,27b-triboranaphtho[3,2,1-*de*] naphtho[3',2',1':10,11]tetraceno[1,2,3-*jk*]pentacene-7,13,19-triamine (V-DABNA-F)**

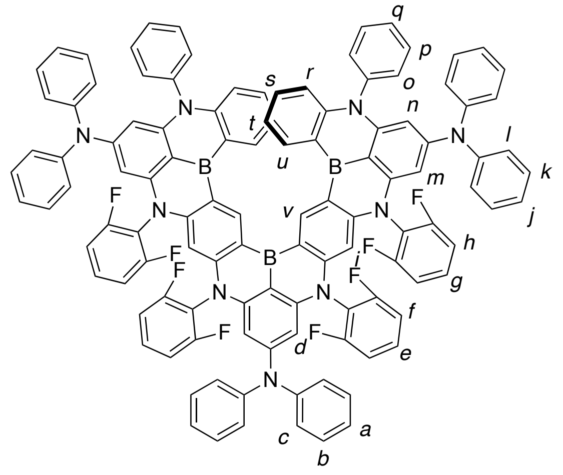


Boron tribromide (76.0 μL, 0.80 mmol) was added to a solution of **5-F** (86.4 mg, 0.050 mmol) in 1,2-dichlorobenzene (1.0 mL) under a nitrogen atmosphere. After stirring at 200 ºC for 18 h, the reaction mixture was allowed to cool to room temperature and hydrogen bromide was removed *in vacuo*. After phosphorus buffer solution (pH = 7, 5.0 mL) was added to the reaction mixture at 0 ºC, the aqueous layer was separated and extracted with dichloromethane (20 mL, three times). After the solvent was removed *in vacuo*, the crude product was purified by silica gel column chromatography (eluent: hexane/dichloromethane = 1/1) to obtain the title compound (30.2 mg, 35% yield, 97% pure on NMR analysis) as a yellow solid. IR (neat): $cm^{-1}$ 3036 (Ar–H), 1576, 1474, 1427, 1296, 1240, 1157, 1013, 789, 750, 696; mp: >300 ºC, $^{1}H$ NMR ($CDCl_3$, 400 MHz) δ 5.71 (s, 2H, *m* or *n*), 5.75 (s, 2H, *d* or *i*), 5.79 (s, 2H, *m* or *n*), 5.85 (s, 2H, *d* or *i*), 6.36 (t, *J* = 7.4 Hz, 2H, *t*), 6.51 (d, *J* = 8.4 Hz, 2H, *r*), 6.76–6.80 (m, 8H, *f, h*), 6.88–6.91 (m, 8H, *a, j, s*), 6.96–7.17 (m, 28H, *b, c, e, g, k, l*), 7.24 (d, *J* = 9.2 Hz, 4H, *o*), 7.33 (d, *J* = 7.6 Hz, 2H, *q*), 7.45 (t, *J* = 7.6 Hz, 4H, *p*), 9.00 (d, *J* = 7.6 Hz, 2H, *u*), 10.6 (s, 2H, *v*); $^{19}F$ NMR ($(CDCl_2)_2$, 376 MHz) δ –117.4; $^{11}B$ NMR (128 MHz, $(CDCl_2)_2$) δ 30.3; HRMS (MALDI-TOF/MS) *m/z* $[M]^+$ calcd for $C_{114}H_{70}B_3F_8N_9$ 1749.5941, observed 1749.5962. $^{13}C$ NMR peaks were barely detected because of the low solubility of **V-DABNA-F**.

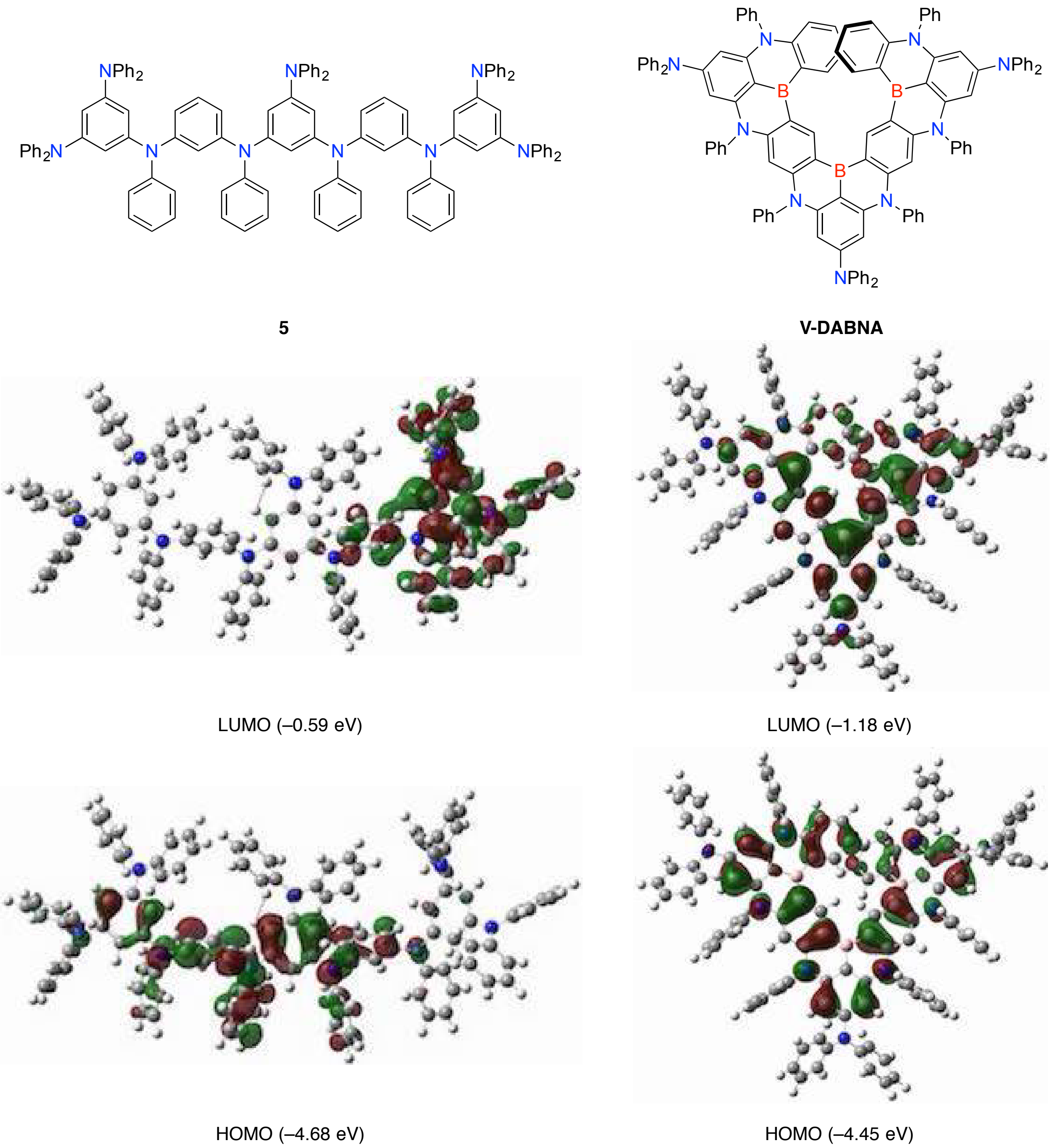


**Figure S1.** Kohn–Sham frontier orbitals of **5** and **V-DABNA** calculated at the B3LYP/6-31G(d) level of theory (Gaussian 09).

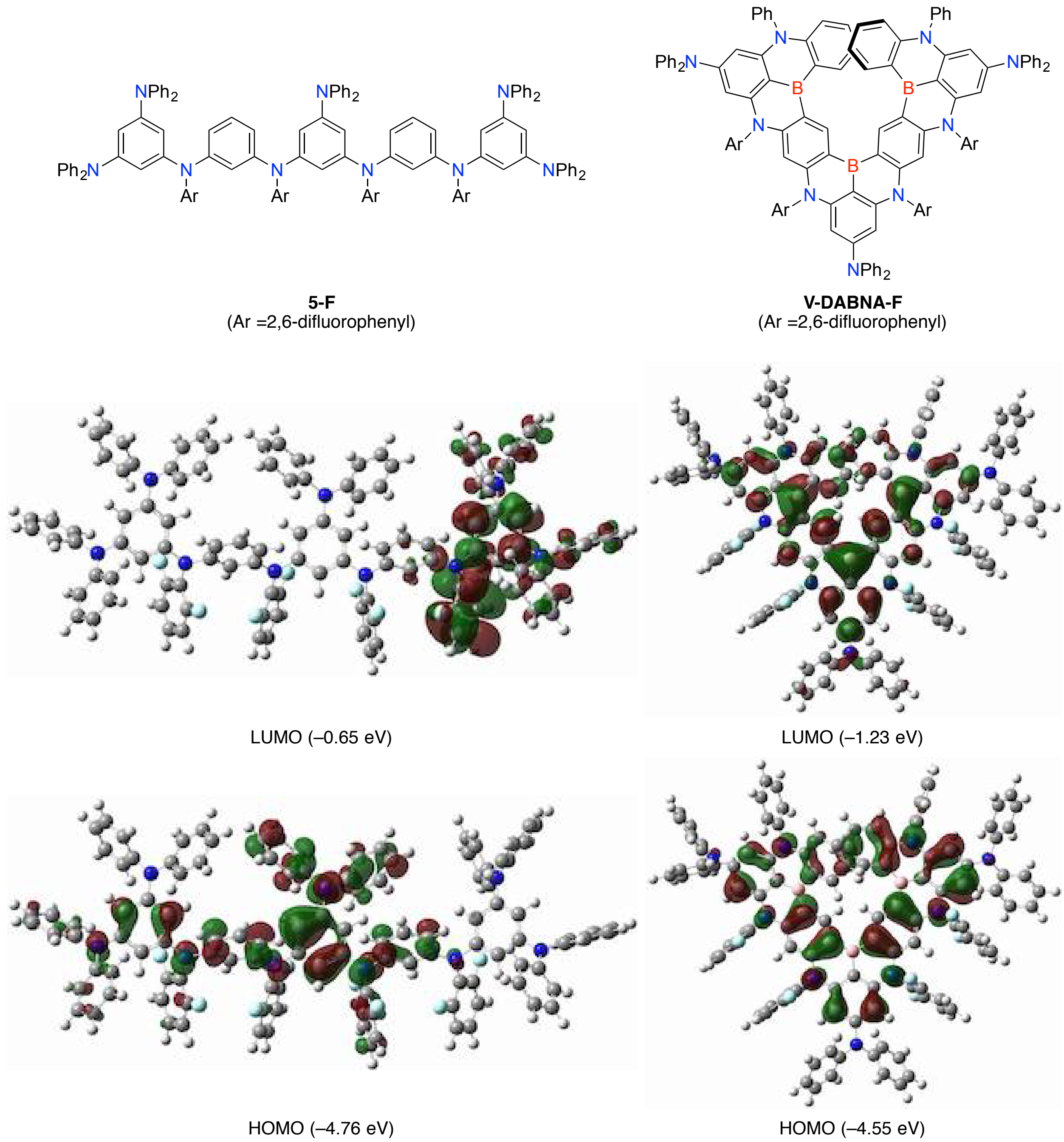


**Figure S2.** Kohn–Sham frontier orbitals of **5-F** and **V-DABNA-F** calculated at the B3LYP/6-31G(d) level of theory (Gaussian 09).

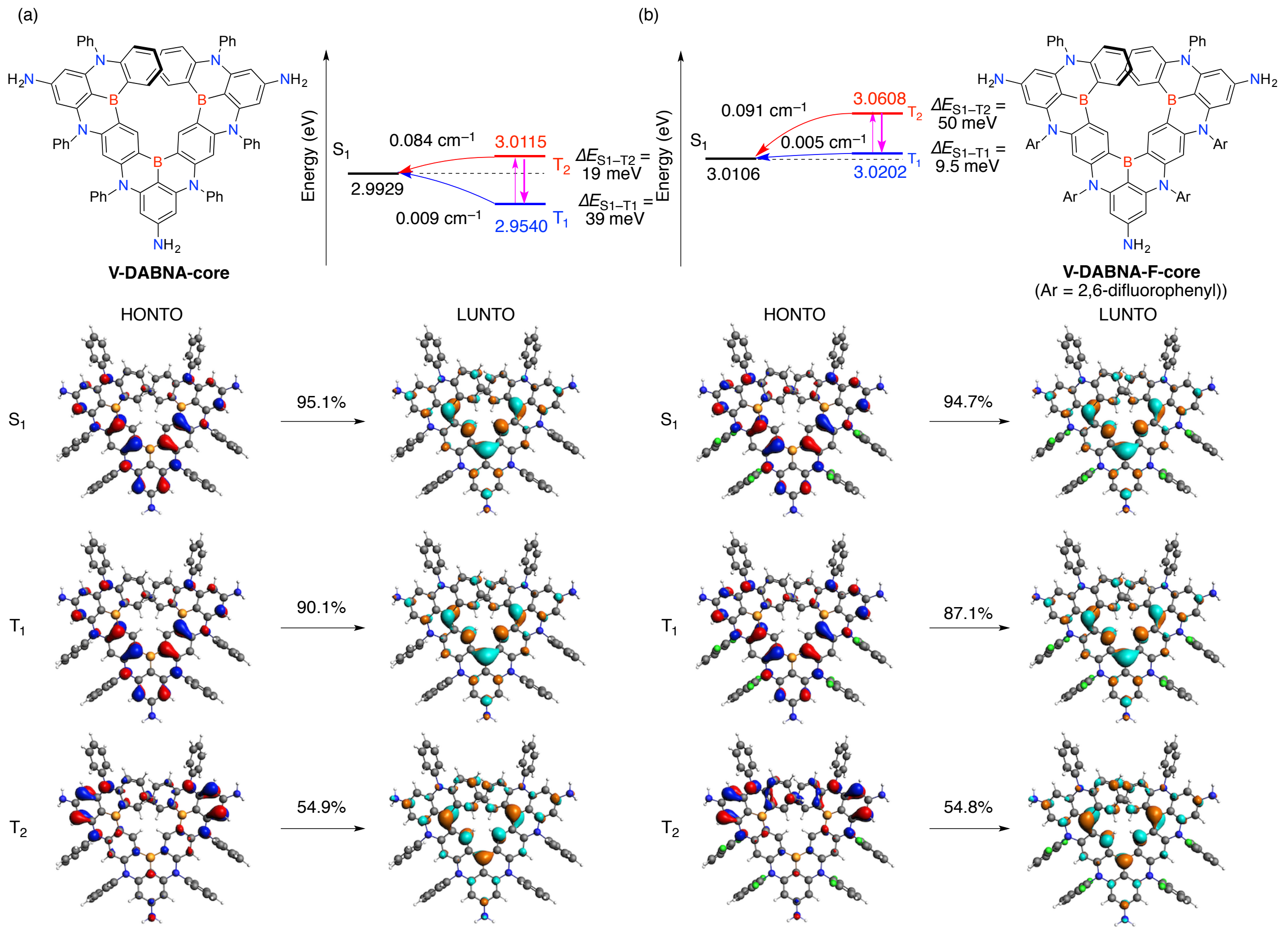


**Figure S3.** Molecular structures, energy-level diagrams, and associated natural transition orbitals (NTOs) for the singlet and triplet excited states of (a) **V-DABNA-core** and (b) **V-DABNA-F-core**. Transition energies for $S_1$, $T_1$, and $T_2$ were calculated at the ADC(2)-COSMO/def2SVP//B3LYP/TZP level of theory. NTOs and spin−orbit coupling (SOC) matrix elements between the $T_n$ and $S_n$ states were calculated at the B3LYP/TZP//B3LYP/TZP level of theory (ADF2021).

## Spin-Orbit Coupling Calculations

Using the optimized geometries in the $T_1$ state, the spin-orbit coupling (SOC) matrix elements $\langle S_1|\hat{H}_{SOC}|T_n\rangle$ between $S_1$ and $T_n$ (n = 1, 2) were obtained performing TD-DFT calculations with the B3LYP functional and the TZP basis set as implemented in the ADF2021 program. Taking into account the contributions from the three degenerate triplet states ($T_{n,x}$, $T_{n,y}$, and $T_{n,z}$), $\langle S_1|\hat{H}_{SOC}|T_n\rangle$ were calculated by the root sum square of the real and imaginary parts (*Re* and *Im*) of the matrix elements (eq. S1).

$$\langle S_1|\hat{H}_{SOC}|T_n\rangle = \sum_{\alpha=x,y,z}\left[\left(Re\langle S_1|\hat{H}_{SOC}|T_{n,\alpha}\rangle\right)^2 + \left(Im\langle S_1|\hat{H}_{SOC}|T_{n,\alpha}\rangle\right)^2\right]^{\frac{1}{2}} \quad \text{(S1)}$$

The resonance effect of nitrogen atoms localizes HOMO on the carbons at their *ortho* and *para* positions, which potentially undergo electrophilic borylation. The introduction of fluorine atoms fully suppressed the undesired borylation at the *ortho* carbons on the side phenyl groups (indicated by red circle). Moreover, their inductive effect reduces electron density at the *para* carbons on the side phenyl groups (indicated by blue circle) and the *ortho* carbons on the main framework (indicated by green circle), which can suppress the overborylation.

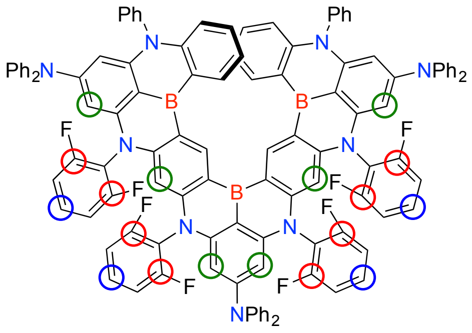


**V-DABNA-F**

**Figure S4.** Molecular structure of **V-DABNA-F**.

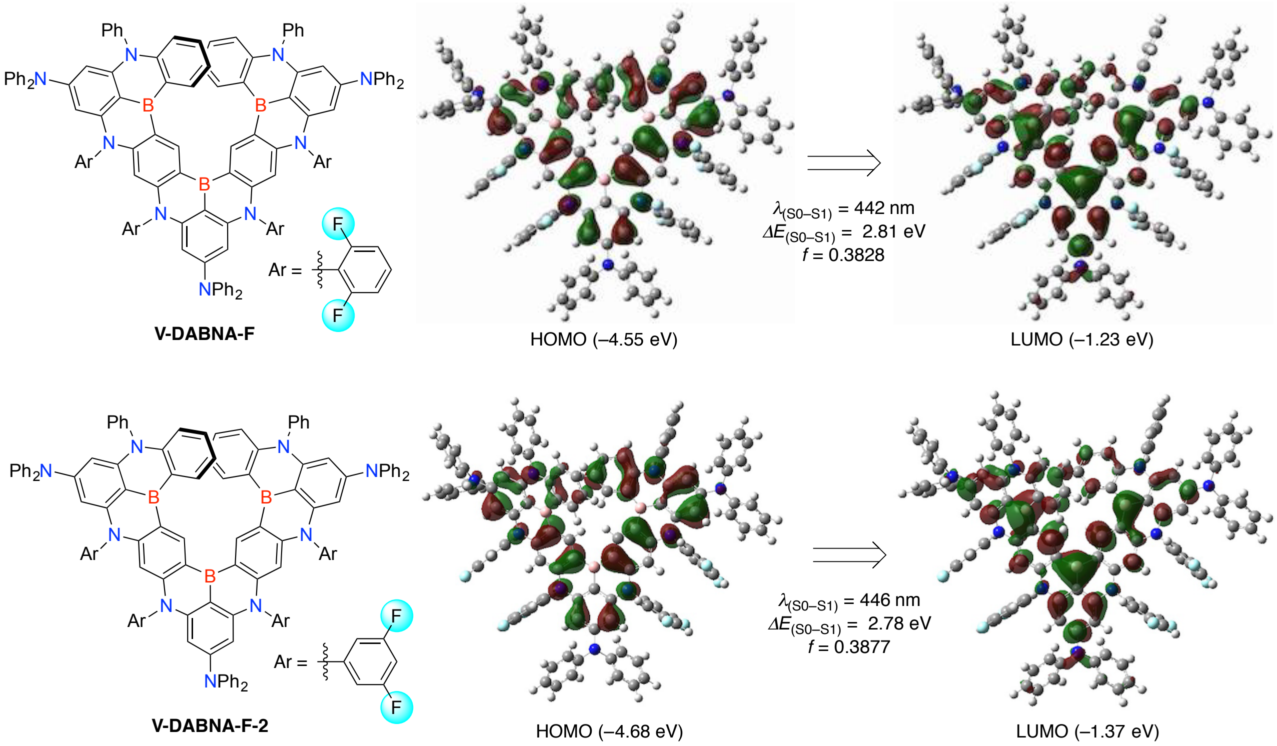


**Figure S5.** Chemical structures and Kohn–Sham frontier orbitals of **V-DABNA-F** and **V-DABNA-F-2** with the oscillator strength ($f$) and $S_0$–$S_1$ transition energies ($\Delta E$, $\lambda$) at the B3LYP/6-31G(d) level of theory.

### Measurement of absorption and emission characteristics

UV-visible absorption spectra were measured using a UV-2600 (Shimadzu) and a V-560 UV-visible spectrometer (JASCO) at 298 K. Photoluminescence (PL) spectra were measured using a FluoroMax-4P (HORIBA) and a F-7000 (Hitachi High-Tech) at 77 and 298 K. Furthermore, the absolute PL quantum yields were measured using C11347 and C9920-02G spectrometers (Hamamatsu Photonics), whereas PL decays were measured using a C11367 spectrometer (298 K, Hamamatsu Photonics) and then fitted using a single exponential function to determine the lifetimes of prompt and delayed fluorescence.

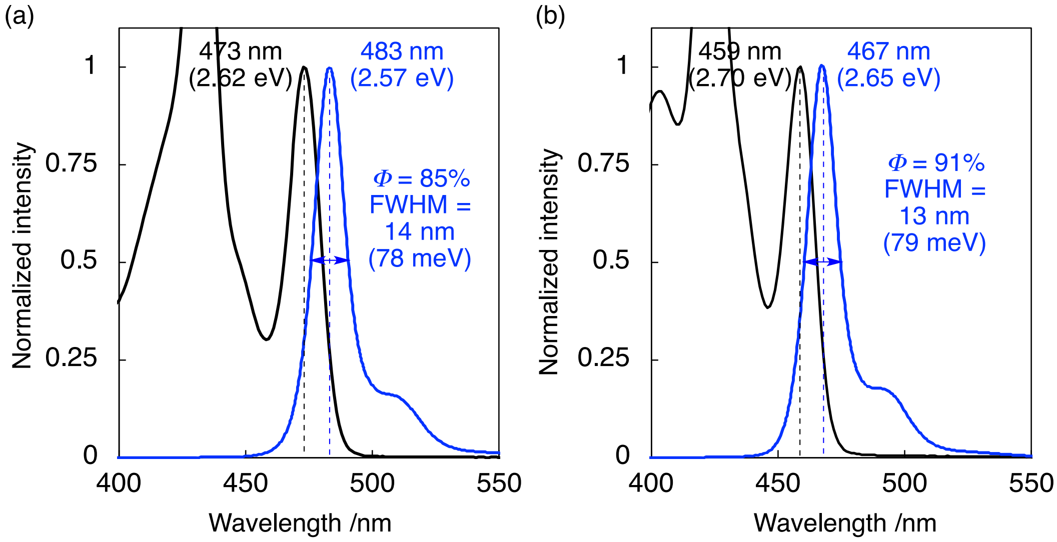


**Figure S6.** Absorption (black) and fluorescence (blue) spectra of (a) **V-DABNA** and (b) **V-DABNA-F** at 300 K in toluene (1.0 × $10^{-5}$ M) with absorption/emission maxima (nm, eV), absolute fluorescence quantum yield (*Φ*), and full width at half maximum (nm).

**Estimation of rate constant for reverse intersystem crossing.** Rate constants ($k_F$, $k_{IC}$, $k_{ISC}$, ,$k_{TADF}$, and $k_{RISC}$) were determined from the measurements of quantum yields ($\Phi_F$ and $\Phi_{TADF}$) and lifetimes ($\tau_F$, $\tau_{TADF}$) and of the prompt (fluorescence) and delayed (TADF) components according to Adachi's method (equations 1–2[9], 3–5[10] and 6[11]).

$\Phi = 0.852$

$\Phi_F = 0.800$

$\Phi_{TADF} = 0.052$

$\tau_F = 6.28$ ns

$\tau_{TADF} = 1.50$ µs

| | | |
|---|---|---|
| $k_p = 1.59 \times 10^8$ s$^{-1}$ | $k_p = 1/\tau_F$ | (1) |
| $k_d = 6.67 \times 10^5$ s$^{-1}$ | $k_d = 1/\tau_{TADF}$ | (2) |
| $k_F = 1.27\times 10^8$ s$^{-1}$ | $k_F = \Phi_F/\tau_F$ | (3) |
| $k_{IC} = 2.21 \times 10^7$ s$^{-1}$ | $\Phi = k_F/(k_F + k_{IC})$ | (4) |
| $k_{ISC} = 0.96 \times 10^7$ s$^{-1}$ | $\Phi_F = k_F/(k_F + k_{IC} + k_{ISC})$ | (5) |
| $k_{RISC} = 7.14 \times 10^5$ s$^{-1}$ | $k_{RISC} = k_p k_d/(k_p - k_{ISC})$ | (6) |

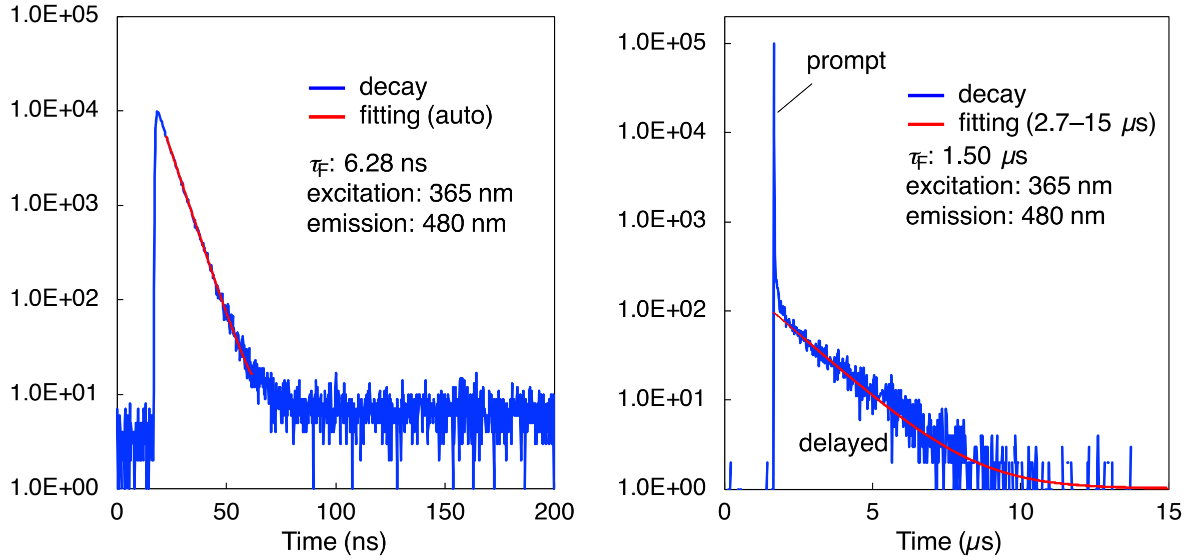


**Figure S7.** Transient decay spectra of **V-DABNA** in toluene ($1.0 \times 10^{-5}$ M). A red curve is single exponential fitting data.

(9) Masui, K.; Nakanotani, H.; Adachi, C. *Org. Electron.* **2013**, *14*, 2721.
(10) Zhang, Q.; Kuwabara, H.; Potscavage, W. J.; Huang, S.; Hatae, Y.; Shibata, T.; Adachi, C. *J. Am. Chem. Soc.* **2014**, *136*, 18070–18081.
(11) Kaji, H.; Suzuki, H.; Fukushima, T.; Shizu, K.; Katsuaki, K.; Kubo, S.; Komino, T.; Oiwa, H.; Suzuki, F.; Wakamiya, A.; Murata, Y.; Adachi, C. *Nat. Commun.* **2015**, *6*, 8476.

$\Phi = 0.908$

$\Phi_F = 0.850$

$\Phi_{TADF} = 0.058$

$\tau_F = 6.32$ ns

$\tau_{TADF} = 2.12$ μs

| | | |
|---|---|---|
| $k_p = 1.58 \times 10^8$ s$^{-1}$ | $k_p = 1/\tau_F$ | (1) |
| $k_d = 4.72 \times 10^5$ s$^{-1}$ | $k_d = 1/\tau_{TADF}$ | (2) |
| $k_F = 1.34 \times 10^8$ s$^{-1}$ | $k_F = \Phi_F/\tau_F$ | (3) |
| $k_{IC} = 1.36 \times 10^7$ s$^{-1}$ | $\Phi = k_F/(k_F + k_{IC})$ | (4) |
| $k_{ISC} = 1.01\times 10^7$ s$^{-1}$ | $\Phi_F = k_F/(k_F + k_{IC} + k_{ISC})$ | (5) |
| $k_{RISC} = 5.06 \times 10^5$ s$^{-1}$ | $k_{RISC} = k_p k_d/(k_p - k_{ISC})$ | (6) |

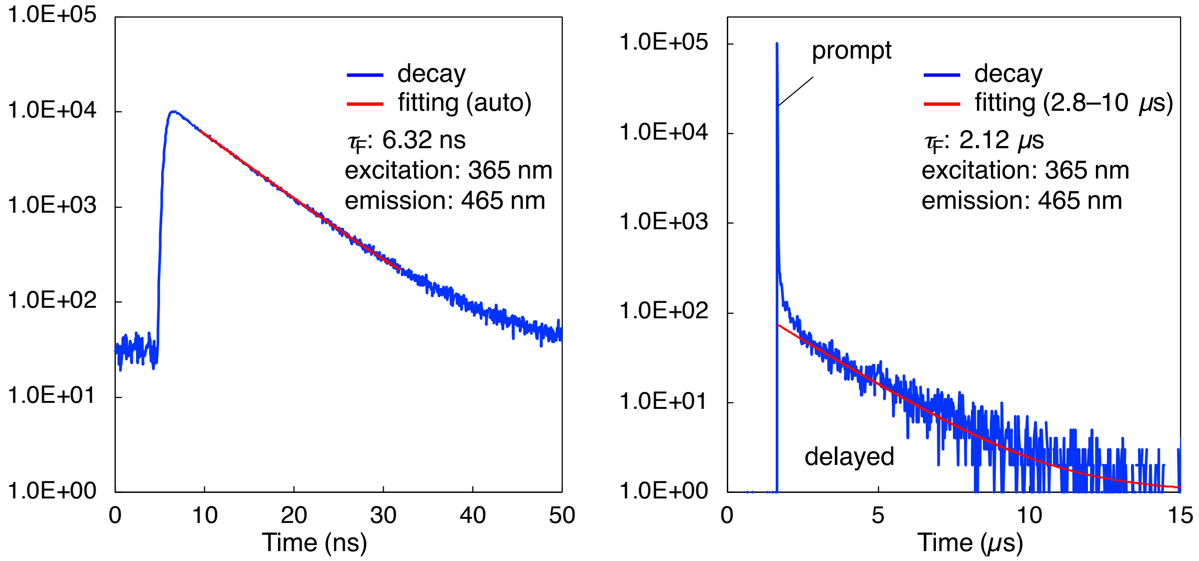


**Figure S8.** Transient decay spectra of **V-DABNA-F** in toluene ($1.0 \times 10^{-5}$ M). A red curve is single exponential fitting data.

**Device fabrication and measurement of electroluminescence characteristics.** OLEDs were fabricated on glass substrates coated with a patterned transparent ITO conductive layer. The substrates were cleaned in a detergent solution at 60 °C for 5 min and then in distilled water at room temperature (298 K) for 10 min in an ultrasonic bath. Subsequently, they were pre-dried using a jet spin washer, and then dried for 5 min in an oven at 423 K, before being finally treated with UV/ozone plasma. OLED employing **V-DABNA** or **V-DABNA-F** as an emitter with the following structure was fabricated: indium tin oxide (ITO, 50 nm); *N,N'*-di(1-naphthyl)-*N,N'*-diphenyl-(1,1'-biphenyl)-4,4'-diamine (NPD, 40 nm); tris(4-carbazolyl-9-ylphenyl)amine (TCTA, 15 nm); 1,3-bis(*N*- carbazolyl)benzene (mCP, 15 nm); 0.5 wt% emitter and 99.5 wt% of **DOBNA-Tol** (20 nm); 3,4-di(9H- carbazol-9-yl)benzonitrile (3,4-2CzBN, 10 nm); 2,7-di([2,2'-bipyridin]-5-yl)triphenylene (BPy-TP2, 20 nm); LiF (1 nm); Al (100 nm). The pressure during the vacuum evaporation was 5.0 × $10^{-4}$ Pa, and the film thickness was controlled using a calibrated quartz crystal microbalance during deposition. After the deposition of all layers, the OLED test modules were encapsulated with a capping glass in an evaporation chamber filled with nitrogen. The OLED characteristics of all fabricated devices were evaluated at room temperature (298 K) in an air atmosphere using a voltage–current–luminance measuring system, comprising a source meter (Keithley 2400) and spectral radiance meter (Topcon SR-3AR). The EQE was calculated using the EL spectrum, assuming the light-emitting surface to be a perfect diffusion surface and adding up all radiance elements from every angle and inputting them into the formula for obtaining EQE.

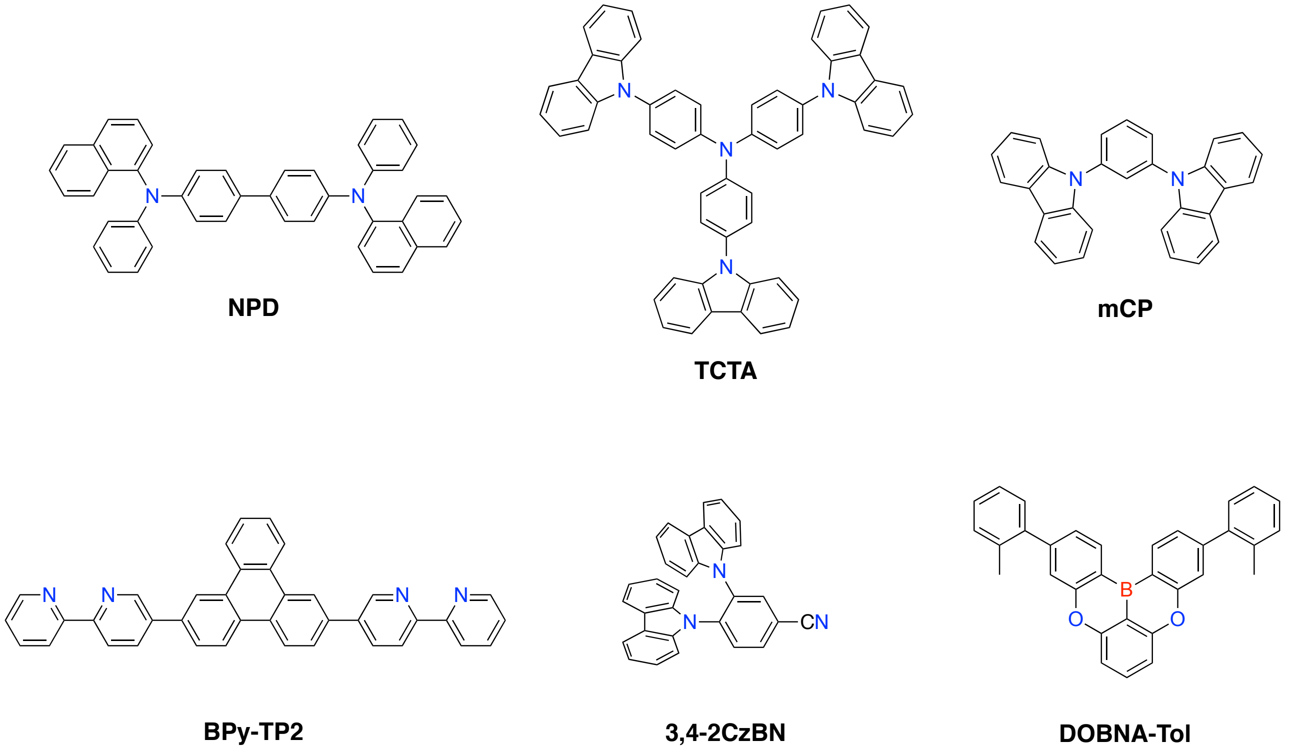


**Figure S9.** Molecular structures of OLED materials employed in this study.

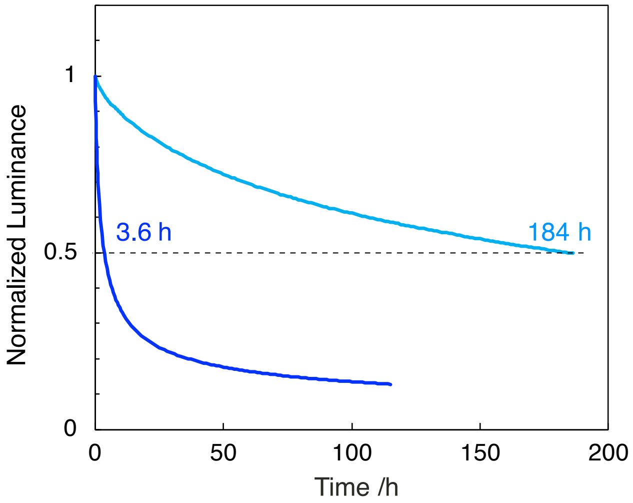


**Figure S10.** Normalized luminance versus time characteristics of OLED device employing **V-DABNA** (sky-blue) and **V-DABNA-F** (blue) with initial luminance at 500 cd $m^{-2}$.

**Table S4.** Summary of the Electroluminescent Data of the **V-DABNA**- and **V-DABNA-F**-based Devices.

| compound | $\lambda_{em}$ [a] [nm] | FWHM [b] [nm] | CIE (x,y) [c] | $V_{on}$ [d] [V] | EQE [e] [%] | $\eta$ [f] [cd $A^{-1}$] | $\eta$ [g] [lm $W^{-1}$] |
|---|---|---|---|---|---|---|---|
| **V-DABNA** | 483 | 17 | (0.09, 0.27) | 3.4 | 26.2/26.2/26.2/25.3 | 36.2/35.8/35.6/34.1 | 33.1/29.6/26.1/20.9 |
| **V-DABNA-F** | 468 | 15 | (0.12, 0.10) | 3.2 | 26.6/26.1/25.8/23.4 | 22.3/21.7/21.3/19.2 | 21.5/18.6/15.7/11.4 |

[a]Maximum wavelength of electroluminescence. [b]Full-width at half-maximum. [c]CIE coordinate. [d]Turn-on voltage at 1 cd $m^{-2}$. [e]External quantum efficiency maximum/at 10/100/1000/10000 cd $m^{-2}$. [f]Current efficiency maximum/at 10/100/1000/10000 cd $m^{-2}$. [g]Power efficiency maximum/ at 10/100/1000/10000 cd $m^{-2}$.

**Measurement of Angular dependent photoluminescence spectroscopy.**

The optical constants of the host material (**DOBNA-Tol**) were investigated by variable angle spectroscopic ellipsometry with a FE-5000 ellipsometer (Otsuka Electronics). The thickness of the deposited film was 60 nm. The thin films of 1 wt% emitter (**V-DABNA**, **V-DABNA-F**) and 99 wt% **DOBNA-Tol** were co-evaporated in high vacuum on a pre-cleaned quartz glass substrate. This substrate was then encapsulated with a capping glass under a nitrogen atmosphere. The thickness of the deposited film was 20 nm, respectively. The angular dependent photoluminescence spectroscopy of the deposited film was measured from –90° to +90° using C14234-01 (excited at 365 nm, Hamamatsu Photonics).

To evaluate dipole orientation, far-field emission intensity as a function of emission angle was simulated using a commercial software package (setfos 3.4, Fluxim Co.) with an optical model including refractive index (n), extinction coefficient (k), thickness, and dipole orientation pz/px, where substituted z and x indicate the direction of vertical and horizontal to a substrate. Herein, n and k were estimated by variable-angle spectroscopic ellipsometry (VASE) analysis.

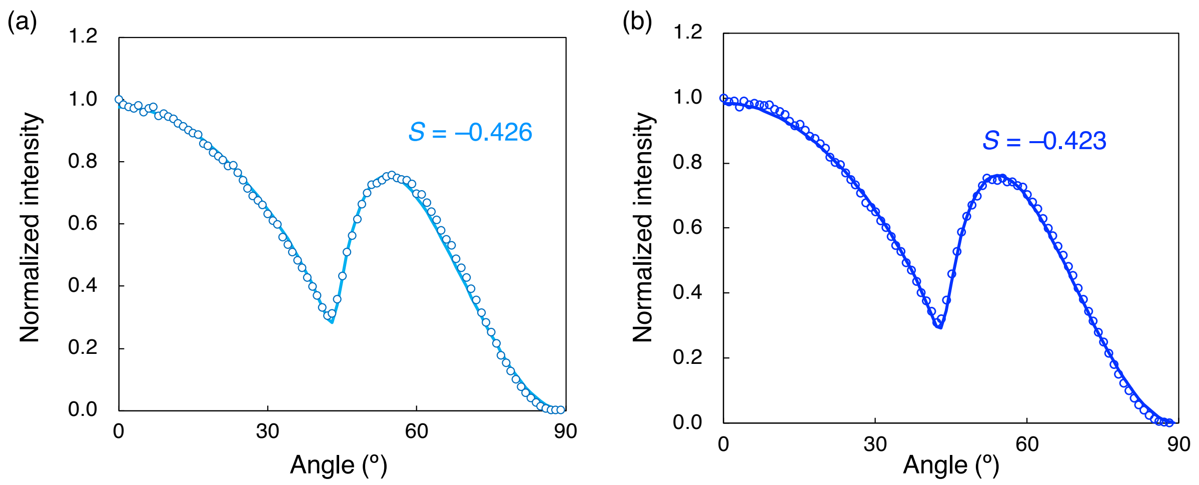


**Figure S11.** Angular dependence of the p-polarized photoluminescence of (a) **V-DABNA** and (b) **V-DABNA-F** in **DOBNA-Tol** (1 wt%-doped film, respectively) with orientational order parameters (*S*). The dots represent the experimental measurements, the full lines the simulated results.

**Table S5.** Summary of OLED Performance Employing blue MR-TADF emitter.

| Emitter | $\lambda_{EL}$[a] [nm] | FWHM[b] [nm] | CIE[c] (x, y) | $V_{on}$[d] [nm] | EQE[e] [%] | roll-off[f] [%] | $CE_{max}$[g] [cd $A^{-1}$] | $PE_{max}$[h] [lm $W^{-1}$] | ref |
|---|---|---|---|---|---|---|---|---|---|
| **V-DABNA** | 483 | 17 | (0.09, 0.27) | 3.4 | 26.2/26.2/26.2/25.3 | 0.9 | 36.2 | 33.1 | this work |
| **V-DABNA-F8** | 468 | 15 | (0.12, 0.10) | 3.2 | 26.6/26.1/25.8/23.4 | 3.2 | 22.3 | 21.5 | this work |
| **DABNA-1** | 459 | 28 | (0.13, 0.09) | - | 13.5/(12)/(6.0)/- | >6.5 | 10.6 | 8.3 | 12 |
| **DABNA-2** | 467 | 28 | (0.12, 0.13) | - | 20.2/(19)/13.4/- | >6.8 | 21.1 | 15.1 | 12 |
| **B2** | 460 | 37 | (0.13, 0.11) | 3.8 | 18.3/(16)/12.6/- | >5.7 | 16.7 | 13.8 | 13 |
| **ν-DABNA** | 469 | 18 | (0.12, 0.11) | 3.4 | 34.4/(34)/32.8/26.0 | 8.4 | 31 | 25.6 | 14 |
| **DABNA-NP-TB** | 457 | 33 | (0.14, 0.11) | 3.6 | 19.5/(18)/17.5/12.0 | 7.5 | 18.5 | 16.1 | 15 |
| **ν-DABNA-O-Me** | 465 | 23 | (0.13, 0.10) | 3.4 | 29.5/29.1/28.8/26.9 | 2.6 | 24.6 | 22.7 | 16 |
| **V-DABNA-Mes** | 480 | 17 | (0.09, 0.21) | 3.2 | 22.9/22.9/20.3/10.9 | 12.0 | 26.7 | 20 | 17 |
| **BBCz-DB** | 469 | 27 | (0.12, 0.18) | - | 29.3/(8.0)/-/- | >21.3 | - | - | 18 |
| **QA-1** | 455 | 39 | (0.14, 0.12) | 4.4 | 17.1/-/1.2/- | >15.9 | 18.1 | 12.9 | 19 |
| **γ-Cb-B** | 461 | 28 | (0.13, 0.13) | - | 19.0/(17)/16.2/7.7 | 11.3 | 17.8 | 19 | 20 |
| **BOBO-Z** | 445 | 18 | (0.15, 0.04) | 4.5 | 13.6/(12)/9.8/3.3 | 10.3 | 7.2 | 5 | 21 |
| **BOBS-Z** | 456 | 23 | (0.14, 0.06) | 4.0 | 26.9/(26)/24.0/15.0 | 11.9 | 16.7 | 12.9 | 21 |
| **BSBS-Z** | 463 | 22 | (0.13, 0.08) | 4.0 | 26.8/(26)/24.0/15.9 | 10.9 | 23.2 | 15 | 21 |
| **CzBO** | 448 | 30 | (0.15, 0.05) | 4.1 | 13.4/(12)/8.4/3.5 | 9.9 | 7.4 | 5.7 | 22 |
| **CzBS** | 473 | 31 | (0.11, 0.16) | 4.0 | 23.1/(22)/21.3/15.0 | 8.1 | 25.8 | 19.4 | 22 |
| **CzBSe** | 481 | 33 | (0.10, 0.24) | 3.9 | 23.9/(23)/23.4/20.0 | 3.9 | 34.8 | 25.8 | 22 |
| **CzBNO** | 454 | 36 | (0.14, 0.08) | 4.0 | 13.6/-/11.0/5.0 | 8.6 | 14.7 | - | 23 |
| **DMAcBNO** | 472 | 41 | (0.13, 0.19) | 4.0 | 20.4/-/18.1/8.6 | 11.8 | 31.2 | - | 23 |
| **DPAcBNO** | 468 | 37 | (0.13, 0.14) | 4.0 | 23.0/-/22.3/9.1 | 13.9 | 28 | - | 23 |
| **BN1** | 457 | 28 | (0.14,0.08) | 4.3 | 31.2/(30)/18.3/9.3 | 21.9 | 20.9 | 14.9 | 24 |
| **BN2** | 467 | 23 | (0.13, 0.11) | 4.3 | 33.2/-/25.5/15.5 | 17.7 | 29.7 | 20.3 | 24 |
| **BN3** | 458 | 23 | (0.14, 0.08) | 4.1 | 37.6/34.0/26.2 | 11.4 | 27.5 | 19 | 24 |
| **B-O-dpa** | 443 | 32 | (0.15, 0.05) | 3.8 | 16.3/6.5/2.2/- | >14.1 | 8.3 | - | 25 |
| **t-DAB-DPA** | 459 | 26 | (0.14, 0.08) | - | 27.6/(26)/21.8/9.2 | 18.4 | - | - | 26 |
| **tPBisICz** | 452 | 21 | (0.15, 0.05) | - | 23.1/(9.0)/(5.0)/- | >18.1 | 13.3 | 13.5 | 27 |
| **4F-ν-DABNA** | 464 | 18 | (0.13, 0.08) | 3.7 | 35.8/(35)/(26)/- | >9.8 | 26.8 | - | 28 |
| **4F-m-ν-DABNA** | 461 | 18 | (0.13, 0.06) | 3.7 | 33.7/(30)/(25)/- | >8.7 | 24.9 | - | 28 |
| **tCBNDADPO** | 468 | 24 | (0.12, 0.16) | 3.6 | 13.8/(12)/8.9/3.2 | 10.6 | 19.6 | 17.1 | 29 |
| **mBP-DABNA-Me** | 468 | 28 | (0.12, 0.14) | - | 24.3/-/19.5/- | >4.8 | 24.4 | 21.3 | 30 |
| **R-DOBN** | 464 | 35 | (0.13, 0.12) | 4.2 | 25.6/(20)/13.9/8.6 | 17.0 | 24.1 | 25.6 | 31 |

[a]Maximum wavelength of EL spectrum. [b]Full width at half maximum. [c]CIE (x, y) coordinates. [d]Turn-on voltage at the luminescence of 1 cd $m^{-2}$. [e]External quantum efficiency of maximum/at 10/100/1000 cd $m^{-2}$. The EQE values in parentheses were estimated from EQE-luminance plot. [f]Efficiency roll-off at 1000 cd $m^{-2}$ from $EQE_{max}$. [g]Maximum current efficiency. [h]Maximum power efficiency.


(12) Hatakeyama, T.; Shiren. K.; Nakajima, K.; Nomura, S.; Nakatsuka, S.; Kinoshita, K.; Ni, J.; Ono, Y.; Ikuta, T. *Adv. Mater.* **2016**, *28*, 2777.

(13) Matsui, K.; Oda, S.; Yoshiura, K.; Nakajima, K.; Yasuda, N.; Hatakeyama, T. *J. Am. Chem. Soc.*, **2018**, *140*, 1195.

(14) Kondo, Y.; Yoshiura, K.; Kitera, S.; Nishi, H.; Oda, S.; Gotoh, H.; Sasada, Y.; Yanai, M.; Hatakeyama, T. *Nat. Photonics* **2019**, *13*, 678.

(15) Oda, S.; Kumano, W.; Hama, T.; Kawasumi, R.; Yoshiura, K.; Hatakeyama, T. *Angew. Chem., Int. Ed.* **2021**, *60*, 2882.

(16) Tanaka, H.; Oda, S.; Ricci, G.; Gotoh, H.; Tabata, K.; Kawasumi, R.; Beljonne, D.; Olivier, Y.; Hatakeyama, T. *Angew. Chem., Int. Ed.* **2021**, *60*, 17910.

(17) Oda, S.; Kawakami, B.; Yamasaki, Y.; Matsumoto, R.; Yoshioka, M.; Fukushima, D.; Nakatsuka, S.; Hatakeyama, T. *J. Am. Chem. Soc.* **2022**, *144*, 106.

(18) Yang, M.; Park, I. S.; Yasuda, T. *J. Am. Chem. Soc.* **2020**, *142*, 19468.

(19) Min, H.; Park, I. S.; Yasuda, T. *Angew. Chem. Int. Ed.* **2021**, *60*, 7643.
(20) Yang, M.; Shikita, S.; Min, H.; Park, I. S.; Shibata, H.; Amanokura, N.; Yasuda, T. *Angew. Chem. Int. Ed.* **2021**, *60*, 23142.
(21) Park, I. S.; Yang, M.; Shibata, H.; Amanokura, N.; Yasuda, T. *Adv. Mater.* **2022**, *34*, 2107951.
(22) Park, I. S.; Min, H.; Yasuda, T. *Angew. Chem. Int. Ed.* **2022**, *61*, e202205684.
(23) Han, J.; Huang, Z.; Lv, X.; Miao, J.; Qiu, Y.; Cao, X.; Yang, C. *Adv. Optical Mater.* **2022**, *10*, 2102092.
(24) Lv, X.; Miao, J.; Liu, M.; Peng, Q.; Zhong, C.; Hu, Y.; Cao, X.; Wu, H.; Yang, Y.; Zhou, C.; Ma, J.; Zou, Y.; Yang, C. *Angew. Chem. Int. Ed.* **2022**, e202201588.
(25) Park, J.; Lim, J.; Lee, J. H.; Jang, B.; Han, J. H.; Yoon, S. S.; Lee, J. Y. *ACS Appl. Mater. Interfaces* **2021**, *13*, 45798.
(26) Kim, J. H.; Chung, W. J.; Kim, J.; Lee, J. Y. *Materials Today Energy* **2021**, *21*, 100792.
(27) Patil, V. V.; Lee, H. L.; Kim, I.; Lee, K. H.; Chung, W. J.; Kim, J.; Park, S.; Choi, H.; Son, W.; Jeon, S. O.; Lee, J. Y. *Adv. Sci.* **2021**, *8*, 2101137.
(28) Rayappa Naveen, K.; Lee, H.; Braveenth, R.; Joon Yang, K.; Jae Hwang, S.; Hyuk Kwon, J. *Chem. Eng. J.* **2022**, *432*, 134381.
(29) Bian, J.; Chen, S.; Qiu, L.; Tian, R.; Man, Y.; Wang, Y.; Chen, S.; Zhang, J.; Duan, C.; Han, C.; Xu, H. *Adv. Mater.* **2022**, *34*, 2110547.
(30) Cheon, H. J.; Shin, Y.-S.; Park, N.-H.; Lee, J.-H.; Kim, Y.-H. *Small* **2022**, *18*, 2107574.
(31) Yan, Z.-P.; Yuan, L.; Zhang, Y.; Mao, M.-X.; Liao, X.-J.; Ni, H.-X.; Wang, Z.-H.; An, Z.; Zheng, Y.-X.; Zuo, J.-L. *Adv. Mater.* **2022**, DOI:10.1002/adma.202204253

# Cartesian coordinates

**5** ($S_0$, $C_1$ symmetry)

E(B3LYP/6-31G(d)) = –4889.279901 hartree

| Center Number | Atomic Number | Atomic Type | Coordinates (Angstroms) X | Y | Z |
|---|---|---|---|---|---|
| 1 | 6 | 0 | 9.546346 | -1.527168 | -0.368207 |
| 2 | 6 | 0 | 9.752945 | 1.237545 | -0.331561 |
| 3 | 1 | 0 | 9.489425 | -2.608320 | -0.374555 |
| 4 | 6 | 0 | 8.519595 | 0.670750 | -0.700319 |
| 5 | 6 | 0 | 8.427216 | -0.745936 | -0.709838 |
| 6 | 6 | 0 | 6.013704 | 0.473589 | -2.227224 |
| 7 | 6 | 0 | 4.807498 | 1.189468 | -2.294824 |
| 8 | 6 | 0 | 6.032497 | -0.630033 | -1.315215 |
| 9 | 1 | 0 | 4.781084 | 2.061552 | -2.935352 |
| 10 | 6 | 0 | 4.857398 | -1.019401 | -0.650011 |
| 11 | 1 | 0 | 4.879433 | -1.884258 | -0.004115 |
| 12 | 6 | 0 | 3.630194 | 0.877561 | -1.603789 |
| 13 | 6 | 0 | 3.658863 | -0.307872 | -0.808228 |
| 14 | 6 | 0 | 1.098674 | 1.219887 | -0.300941 |
| 15 | 6 | 0 | -0.179169 | 1.768633 | -0.559442 |
| 16 | 6 | 0 | 1.229179 | -0.194498 | -0.332652 |
| 17 | 6 | 0 | 0.110923 | -1.021783 | -0.519059 |
| 18 | 1 | 0 | 0.222379 | -2.095011 | -0.503604 |
| 19 | 6 | 0 | -1.153036 | -0.447718 | -0.725665 |
| 20 | 6 | 0 | -1.316128 | 0.961757 | -0.798088 |
| 21 | 6 | 0 | -3.551813 | -0.386996 | -0.106211 |
| 22 | 6 | 0 | -4.573253 | -0.913967 | 0.697729 |
| 23 | 6 | 0 | -3.823800 | 0.706139 | -0.983687 |
| 24 | 1 | 0 | -4.369494 | -1.773545 | 1.318343 |
| 25 | 6 | 0 | -5.099466 | 1.275828 | -0.887954 |
| 26 | 1 | 0 | -5.299757 | 2.132750 | -1.518075 |
| 27 | 6 | 0 | -5.858498 | -0.345765 | 0.713663 |
| 28 | 6 | 0 | -6.136294 | 0.832581 | -0.050903 |
| 29 | 6 | 0 | -8.281269 | -0.705879 | 0.375738 |
| 30 | 6 | 0 | -9.329626 | -1.564490 | -0.002720 |
| 31 | 6 | 0 | -8.553463 | 0.649861 | 0.697563 |
| 32 | 1 | 0 | -9.135011 | -2.601070 | -0.247390 |
| 33 | 6 | 0 | -9.889510 | 1.080957 | 0.610408 |
| 34 | 6 | 0 | -10.645803 | -1.090206 | -0.081083 |
| 35 | 7 | 0 | -6.960838 | -1.175481 | 0.445157 |
| 36 | 7 | 0 | -2.267944 | -0.961145 | -0.043748 |
| 37 | 7 | 0 | 2.497950 | -0.771398 | -0.160778 |
| 38 | 7 | 0 | 7.213068 | -1.351784 | -1.067835 |
| 39 | 6 | 0 | -6.735152 | -2.582340 | 0.191959 |
| 40 | 6 | 0 | -6.495928 | -3.018056 | -1.123979 |
| 41 | 6 | 0 | -6.785662 | -3.484719 | 1.265552 |
| 42 | 6 | 0 | -6.308030 | -4.384900 | -1.342246 |
| 43 | 6 | 0 | -6.590052 | -4.844582 | 0.996747 |
| 44 | 6 | 0 | -6.352327 | -5.314811 | -0.296723 |
| 45 | 1 | 0 | -6.124688 | -4.732190 | -2.357219 |
| 46 | 1 | 0 | -6.629287 | -5.552096 | 1.822267 |
| 47 | 6 | 0 | -2.073938 | -2.119292 | 0.801685 |
| 48 | 6 | 0 | -1.718283 | -1.940631 | 2.150446 |
| 49 | 6 | 0 | -2.246770 | -3.398919 | 0.250993 |
| 50 | 6 | 0 | -1.537141 | -3.078403 | 2.940812 |

| | | | | | |
|---|---|---|---|---|---|
| 51 | 6 | 0 | -2.053744 | -4.507610 | 1.082611 |
| 52 | 6 | 0 | -1.697231 | -4.369533 | 2.426129 |
| 53 | 1 | 0 | -1.263832 | -2.951394 | 3.986470 |
| 54 | 1 | 0 | -2.185899 | -5.503964 | 0.665906 |
| 55 | 6 | 0 | 2.606974 | -1.932496 | 0.695784 |
| 56 | 6 | 0 | 2.879088 | -1.746715 | 2.061138 |
| 57 | 6 | 0 | 2.431393 | -3.215274 | 0.148914 |
| 58 | 6 | 0 | 2.974239 | -2.879556 | 2.875800 |
| 59 | 6 | 0 | 2.535086 | -4.316544 | 1.003671 |
| 60 | 6 | 0 | 2.809178 | -4.170876 | 2.367171 |
| 61 | 1 | 0 | 3.180427 | -2.746466 | 3.935915 |
| 62 | 1 | 0 | 2.396021 | -5.313883 | 0.591368 |
| 63 | 6 | 0 | 7.184951 | -2.796217 | -1.151300 |
| 64 | 6 | 0 | 6.867800 | -3.548928 | -0.007478 |
| 65 | 6 | 0 | 7.500023 | -3.414317 | -2.372531 |
| 66 | 6 | 0 | 6.876540 | -4.943104 | -0.111504 |
| 67 | 6 | 0 | 7.494821 | -4.812007 | -2.427304 |
| 68 | 6 | 0 | 7.193301 | -5.593916 | -1.308477 |
| 69 | 1 | 0 | 6.631642 | -5.534655 | 0.768357 |
| 70 | 1 | 0 | 7.736868 | -5.299591 | -3.369386 |
| 71 | 6 | 0 | 10.754435 | -0.919264 | -0.001481 |
| 72 | 6 | 0 | 10.863881 | 0.475225 | 0.022598 |
| 73 | 1 | 0 | 11.791291 | 0.963891 | 0.301835 |
| 74 | 6 | 0 | -10.934106 | 0.244543 | 0.223213 |
| 75 | 1 | 0 | -11.946953 | 0.629486 | 0.170097 |
| 76 | 6 | 0 | -2.722119 | 3.322148 | -1.164844 |
| 77 | 6 | 0 | -3.706832 | 3.921712 | -1.981297 |
| 78 | 6 | 0 | -1.381829 | 3.758336 | -1.332328 |
| 79 | 6 | 0 | -3.406425 | 4.903071 | -2.914823 |
| 80 | 1 | 0 | -4.730044 | 3.568850 | -1.902546 |
| 81 | 6 | 0 | -2.072948 | 5.293176 | -3.082950 |
| 82 | 1 | 0 | -4.189600 | 5.338639 | -3.529170 |
| 83 | 1 | 0 | -1.806786 | 6.026366 | -3.840060 |
| 84 | 6 | 0 | 1.913775 | 3.547723 | 0.400220 |
| 85 | 6 | 0 | 0.586337 | 3.984690 | 0.151773 |
| 86 | 6 | 0 | 2.740675 | 4.391449 | 1.174904 |
| 87 | 6 | 0 | 2.302043 | 5.606090 | 1.681861 |
| 88 | 1 | 0 | 3.743420 | 4.054144 | 1.416525 |
| 89 | 6 | 0 | 0.979288 | 5.999628 | 1.449371 |
| 90 | 1 | 0 | 2.964995 | 6.227042 | 2.278028 |
| 91 | 1 | 0 | 0.598849 | 6.924611 | 1.875162 |
| 92 | 7 | 0 | -0.324357 | 3.166012 | -0.579954 |
| 93 | 6 | 0 | -1.070457 | 4.721919 | -2.311741 |
| 94 | 1 | 0 | -0.038812 | 5.003973 | -2.485311 |
| 95 | 6 | 0 | 0.127106 | 5.196370 | 0.704651 |
| 96 | 1 | 0 | -0.905737 | 5.495427 | 0.571399 |
| 97 | 1 | 0 | 2.273845 | 2.616255 | 0.016008 |
| 98 | 1 | 0 | 1.938209 | 1.848436 | -0.088794 |
| 99 | 1 | 0 | 2.757531 | 1.493348 | -1.668319 |
| 100 | 1 | 0 | -2.976565 | 2.569065 | -0.448587 |
| 101 | 1 | 0 | -3.093442 | 1.069806 | -1.675947 |
| 102 | 1 | 0 | -2.266398 | 1.397918 | -1.025356 |
| 103 | 1 | 0 | -7.772353 | 1.317939 | 0.994949 |
| 104 | 1 | 0 | -7.074556 | 1.343130 | 0.011577 |
| 105 | 1 | 0 | 6.864994 | 0.736274 | -2.819847 |
| 106 | 1 | 0 | 7.683057 | 1.283776 | -0.963573 |
| 107 | 7 | 0 | 11.909103 | -1.754014 | 0.360226 |
| 108 | 7 | 0 | 9.939582 | 2.695000 | -0.288052 |
| 109 | 7 | 0 | -10.264555 | 2.468436 | 0.918858 |
| 110 | 7 | 0 | -11.725398 | -2.001822 | -0.486460 |

| | | | | | |
|---|---|---|---|---|---|
| 111 | 6 | 0 | 11.777871 | -3.217883 | 0.387915 |
| 112 | 6 | 0 | 12.070416 | -3.966220 | -0.752098 |
| 113 | 6 | 0 | 11.360143 | -3.858849 | 1.554912 |
| 114 | 6 | 0 | 11.946600 | -5.355620 | -0.725483 |
| 115 | 1 | 0 | 12.399977 | -3.461420 | -1.671776 |
| 116 | 6 | 0 | 11.235924 | -5.247881 | 1.581304 |
| 117 | 1 | 0 | 11.129481 | -3.268597 | 2.453630 |
| 118 | 6 | 0 | 11.529545 | -5.996380 | 0.441153 |
| 119 | 1 | 0 | 12.177724 | -5.945472 | -1.624260 |
| 120 | 1 | 0 | 10.906721 | -5.753332 | 2.500880 |
| 121 | 1 | 0 | 11.431734 | -7.091422 | 0.462251 |
| 122 | 6 | 0 | 13.195005 | -1.124896 | 0.694243 |
| 123 | 6 | 0 | 13.487560 | -0.795357 | 2.017510 |
| 124 | 6 | 0 | 14.122978 | -0.856998 | -0.312888 |
| 125 | 6 | 0 | 14.708348 | -0.198998 | 2.334533 |
| 126 | 1 | 0 | 12.756626 | -1.006686 | 2.811435 |
| 127 | 6 | 0 | 15.343258 | -0.260347 | 0.004068 |
| 128 | 1 | 0 | 13.892063 | -1.116864 | -1.356174 |
| 129 | 6 | 0 | 15.636153 | 0.068330 | 1.327924 |
| 130 | 1 | 0 | 14.938991 | 0.060331 | 3.377939 |
| 131 | 1 | 0 | 16.074816 | -0.049078 | -0.789444 |
| 132 | 1 | 0 | 16.598300 | 0.538513 | 1.577586 |
| 133 | 6 | 0 | 8.816644 | 3.589552 | -0.603784 |
| 134 | 6 | 0 | 7.998068 | 4.069201 | 0.418513 |
| 135 | 6 | 0 | 8.569395 | 3.958581 | -1.926622 |
| 136 | 6 | 0 | 6.932807 | 4.918843 | 0.118846 |
| 137 | 1 | 0 | 8.192887 | 3.778858 | 1.461100 |
| 138 | 6 | 0 | 7.504084 | 4.807643 | -2.226195 |
| 139 | 1 | 0 | 9.214913 | 3.580222 | -2.732505 |
| 140 | 6 | 0 | 6.685950 | 5.288139 | -1.203361 |
| 141 | 1 | 0 | 6.287820 | 5.297251 | 0.925035 |
| 142 | 1 | 0 | 7.308958 | 5.098657 | -3.268649 |
| 143 | 1 | 0 | 5.846294 | 5.957639 | -1.439743 |
| 144 | 6 | 0 | 11.249157 | 3.257902 | 0.071189 |
| 145 | 6 | 0 | 12.193822 | 3.515806 | -0.921946 |
| 146 | 6 | 0 | 11.547339 | 3.533897 | 1.406153 |
| 147 | 6 | 0 | 13.436402 | 4.050793 | -0.580985 |
| 148 | 1 | 0 | 11.958991 | 3.298755 | -1.974080 |
| 149 | 6 | 0 | 12.789818 | 4.068313 | 1.747010 |
| 150 | 1 | 0 | 10.802367 | 3.330411 | 2.189037 |
| 151 | 6 | 0 | 13.734294 | 4.327157 | 0.753343 |
| 152 | 1 | 0 | 14.180884 | 4.254470 | -1.364187 |
| 153 | 1 | 0 | 13.025124 | 4.285932 | 2.799030 |
| 154 | 1 | 0 | 14.713659 | 4.748646 | 1.022269 |
| 155 | 6 | 0 | -11.415546 | -3.391668 | -0.851452 |
| 156 | 6 | 0 | -11.438932 | -4.390343 | 0.121854 |
| 157 | 6 | 0 | -11.097573 | -3.711804 | -2.171915 |
| 158 | 6 | 0 | -11.145618 | -5.709534 | -0.224806 |
| 159 | 1 | 0 | -11.689830 | -4.138424 | 1.162444 |
| 160 | 6 | 0 | -10.803877 | -5.030622 | -2.518326 |
| 161 | 1 | 0 | -11.079140 | -2.924206 | -2.939147 |
| 162 | 6 | 0 | -10.828278 | -6.029648 | -1.544795 |
| 163 | 1 | 0 | -11.164546 | -6.496828 | 0.542626 |
| 164 | 1 | 0 | -10.553232 | -5.283245 | -3.558918 |
| 165 | 1 | 0 | -10.596901 | -7.069295 | -1.818157 |
| 166 | 6 | 0 | -13.114846 | -1.523591 | -0.526846 |
| 167 | 6 | 0 | -13.631603 | -0.979885 | -1.702672 |
| 168 | 6 | 0 | -13.916810 | -1.613272 | 0.611586 |
| 169 | 6 | 0 | -14.950568 | -0.526760 | -1.741135 |
| 170 | 1 | 0 | -12.999973 | -0.909420 | -2.600068 |

| | | | | | |
|---|---|---|---|---|---|
| 171 | 6 | 0 | -15.235304 | -1.159799 | 0.573195 |
| 172 | 1 | 0 | -13.509129 | -2.041949 | 1.538586 |
| 173 | 6 | 0 | -15.752369 | -0.616858 | -0.603354 |
| 174 | 1 | 0 | -15.357931 | -0.098536 | -2.668401 |
| 175 | 1 | 0 | -15.867597 | -1.230333 | 1.470249 |
| 176 | 1 | 0 | -16.791887 | -0.259609 | -0.633442 |
| 177 | 6 | 0 | -11.672880 | 2.883086 | 0.844122 |
| 178 | 6 | 0 | -12.492123 | 2.776319 | 1.967806 |
| 179 | 6 | 0 | -12.190288 | 3.383251 | -0.351428 |
| 180 | 6 | 0 | -13.828528 | 3.170628 | 1.896999 |
| 181 | 1 | 0 | -12.084569 | 2.382548 | 2.910188 |
| 182 | 6 | 0 | -13.526506 | 3.777003 | -0.422275 |
| 183 | 1 | 0 | -11.544168 | 3.467285 | -1.237297 |
| 184 | 6 | 0 | -14.345608 | 3.671061 | 0.702117 |
| 185 | 1 | 0 | -14.474146 | 3.086787 | 2.783166 |
| 186 | 1 | 0 | -13.934583 | 4.171224 | -1.364366 |
| 187 | 1 | 0 | -15.398928 | 3.981718 | 0.646086 |
| 188 | 6 | 0 | -9.231274 | 3.441267 | 1.302045 |
| 189 | 6 | 0 | -8.632678 | 4.242511 | 0.329980 |
| 190 | 6 | 0 | -8.848971 | 3.562830 | 2.638565 |
| 191 | 6 | 0 | -7.652580 | 5.166344 | 0.693866 |
| 192 | 1 | 0 | -8.934004 | 4.147196 | -0.723282 |
| 193 | 6 | 0 | -7.868763 | 4.486130 | 3.002223 |
| 194 | 1 | 0 | -9.321042 | 2.930929 | 3.404819 |
| 195 | 6 | 0 | -7.270785 | 5.288236 | 2.029876 |
| 196 | 1 | 0 | -7.181079 | 5.798274 | -0.072616 |
| 197 | 1 | 0 | -7.567241 | 4.582168 | 4.055472 |
| 198 | 1 | 0 | -6.498204 | 6.016183 | 2.316825 |
| 199 | 1 | 0 | -6.973272 | -3.144896 | 2.262659 |
| 200 | 1 | 0 | -6.205851 | -6.357125 | -0.489146 |
| 201 | 1 | 0 | -6.463744 | -2.323592 | -1.937358 |
| 202 | 1 | 0 | -1.589476 | -0.961185 | 2.561534 |
| 203 | 1 | 0 | -1.535424 | -5.227080 | 3.045266 |
| 204 | 1 | 0 | -2.519441 | -3.525237 | -0.775942 |
| 205 | 1 | 0 | 3.006004 | -0.765777 | 2.469247 |
| 206 | 1 | 0 | 2.907494 | -5.027736 | 3.000436 |
| 207 | 1 | 0 | 2.218976 | -3.347222 | -0.891456 |
| 208 | 1 | 0 | 6.629154 | -3.070468 | 0.919358 |
| 209 | 1 | 0 | 7.223794 | -6.662267 | -1.359445 |
| 210 | 1 | 0 | 7.740245 | -2.832711 | -3.237937 |

---------------------------------------------------------------------------------------------------------------------------------

**V-DABNA** ($S_0$, $C_1$ symmetry)

E(B3LYP/6-31G(d)) = –4958.631873 hartree

| Center Number | Atomic Number | Atomic Type | Coordinates (Angstroms) X | Y | Z |
|---|---|---|---|---|---|
| 1 | 7 | 0 | 4.481940 | -4.387940 | 1.213531 |
| 2 | 7 | 0 | -2.546283 | 3.858279 | 0.372769 |
| 3 | 7 | 0 | -0.109810 | 8.186462 | 0.347868 |
| 4 | 7 | 0 | -5.506091 | 0.134891 | -0.095758 |
| 5 | 7 | 0 | -9.010868 | -3.402076 | -0.415775 |
| 6 | 7 | 0 | -4.305720 | -4.660944 | -1.145968 |
| 7 | 6 | 0 | 1.262789 | 4.562587 | -0.154552 |
| 8 | 7 | 0 | 8.960585 | -3.196114 | -0.337041 |
| 9 | 6 | 0 | -4.140235 | 0.563093 | -0.128011 |
| 10 | 6 | 0 | 1.362045 | 1.721286 | -0.021445 |

| | | | | | |
|---|---|---|---|---|---|
| 11 | 6 | 0 | -0.089126 | 6.671972 | 0.304687 |
| 12 | 6 | 0 | 4.999172 | -2.154907 | 0.437891 |
| 13 | 6 | 0 | -1.273960 | 4.583911 | 0.274748 |
| 14 | 6 | 0 | -4.827482 | -2.310322 | -0.624893 |
| 15 | 6 | 0 | 5.467562 | -3.517950 | 0.663089 |
| 16 | 6 | 0 | -5.797381 | -1.272353 | -0.251469 |
| 17 | 6 | 0 | 2.543220 | -2.746137 | 1.672312 |
| 18 | 6 | 0 | -3.040855 | -0.296896 | -0.303613 |
| 19 | 6 | 0 | -1.503207 | 1.524846 | -0.151134 |
| 20 | 6 | 0 | -5.358883 | -3.654726 | -0.793790 |
| 21 | 6 | 0 | -2.916058 | -4.427601 | -1.053552 |
| 22 | 6 | 0 | -2.573631 | 2.399309 | 0.209931 |
| 23 | 6 | 0 | 5.978239 | -1.208206 | -0.152891 |
| 24 | 6 | 0 | 7.514266 | -2.892364 | -0.097912 |
| 25 | 6 | 0 | 3.193012 | -4.134170 | 1.759487 |
| 26 | 6 | 0 | -7.501017 | -3.036103 | -0.441224 |
| 27 | 6 | 0 | -2.338436 | -3.151477 | -0.855178 |
| 28 | 6 | 0 | -1.341133 | 8.934475 | 0.055942 |
| 29 | 6 | 0 | -2.108308 | 9.450174 | 1.100318 |
| 30 | 6 | 0 | -1.742696 | 9.128274 | -1.266330 |
| 31 | 6 | 0 | -3.276504 | 10.160775 | 0.823128 |
| 32 | 1 | 0 | -1.791916 | 9.297927 | 2.142411 |
| 33 | 6 | 0 | -2.910876 | 9.838307 | -1.543361 |
| 34 | 1 | 0 | -1.137675 | 8.721520 | -2.089615 |
| 35 | 6 | 0 | -3.677649 | 10.354945 | -0.498586 |
| 36 | 1 | 0 | -3.880977 | 10.567659 | 1.646657 |
| 37 | 1 | 0 | -3.227626 | 9.991206 | -2.585362 |
| 38 | 1 | 0 | -4.598437 | 10.914833 | -0.717228 |
| 39 | 6 | 0 | 1.141555 | 8.864337 | 0.715935 |
| 40 | 6 | 0 | 1.908643 | 9.493411 | -0.264403 |
| 41 | 6 | 0 | 1.562226 | 8.878086 | 2.046372 |
| 42 | 6 | 0 | 3.095877 | 10.137432 | 0.085143 |
| 43 | 1 | 0 | 1.577204 | 9.483057 | -1.312870 |
| 44 | 6 | 0 | 2.749429 | 9.521564 | 2.395688 |
| 45 | 1 | 0 | 0.957268 | 8.381965 | 2.819151 |
| 46 | 6 | 0 | 3.516132 | 10.151630 | 1.415080 |
| 47 | 1 | 0 | 3.700287 | 10.633726 | -0.687854 |
| 48 | 1 | 0 | 3.081241 | 9.532569 | 3.444141 |
| 49 | 1 | 0 | 4.451923 | 10.659020 | 1.690724 |
| 50 | 6 | 0 | -6.608806 | 1.090145 | 0.084271 |
| 51 | 6 | 0 | -7.128404 | 1.771301 | -1.016305 |
| 52 | 6 | 0 | -7.135549 | 1.315758 | 1.356614 |
| 53 | 6 | 0 | -8.175510 | 2.677310 | -0.845458 |
| 54 | 1 | 0 | -6.713757 | 1.593443 | -2.019139 |
| 55 | 6 | 0 | -8.182087 | 2.221917 | 1.527412 |
| 56 | 1 | 0 | -6.725663 | 0.778617 | 2.224237 |
| 57 | 6 | 0 | -8.702388 | 2.902466 | 0.426255 |
| 58 | 1 | 0 | -8.585323 | 3.213909 | -1.713362 |
| 59 | 1 | 0 | -8.597432 | 2.399951 | 2.530042 |
| 60 | 1 | 0 | -9.527553 | 3.616641 | 0.561115 |
| 61 | 6 | 0 | -3.721962 | 4.574290 | 0.888528 |
| 62 | 6 | 0 | -3.966405 | 4.616547 | 2.261001 |
| 63 | 6 | 0 | -4.593805 | 5.211486 | 0.004737 |
| 64 | 6 | 0 | -5.081820 | 5.296803 | 2.750465 |
| 65 | 1 | 0 | -3.279333 | 4.114814 | 2.957735 |
| 66 | 6 | 0 | -5.709191 | 5.891158 | 0.494121 |
| 67 | 1 | 0 | -4.400897 | 5.177981 | -1.077373 |
| 68 | 6 | 0 | -5.953027 | 5.934168 | 1.867112 |
| 69 | 1 | 0 | -5.274118 | 5.330377 | 3.832609 |
| 70 | 1 | 0 | -6.396498 | 6.393436 | -0.202154 |

| | | | | | |
|---|---|---|---|---|---|
| 71 | 1 | 0 | -6.832293 | 6.470211 | 2.252702 |
| 72 | 6 | 0 | 3.465394 | 4.785487 | -0.646019 |
| 73 | 6 | 0 | 3.512309 | 5.672857 | -1.721005 |
| 74 | 6 | 0 | 4.536514 | 4.712272 | 0.245361 |
| 75 | 6 | 0 | 4.629490 | 6.488142 | -1.904546 |
| 76 | 1 | 0 | 2.668216 | 5.731037 | -2.423404 |
| 77 | 6 | 0 | 5.653596 | 5.527054 | 0.061529 |
| 78 | 1 | 0 | 4.499322 | 4.012495 | 1.092842 |
| 79 | 6 | 0 | 5.699948 | 6.415346 | -1.013272 |
| 80 | 1 | 0 | 4.666090 | 7.188008 | -2.751890 |
| 81 | 1 | 0 | 6.497929 | 5.469581 | 0.763864 |
| 82 | 1 | 0 | 6.580614 | 7.057719 | -1.157884 |
| 83 | 6 | 0 | 6.463451 | 1.160765 | -1.021088 |
| 84 | 6 | 0 | 7.326224 | 1.848760 | -0.168148 |
| 85 | 6 | 0 | 6.510406 | 1.392647 | -2.396312 |
| 86 | 6 | 0 | 8.237000 | 2.767888 | -0.689840 |
| 87 | 1 | 0 | 7.289704 | 1.665967 | 0.915593 |
| 88 | 6 | 0 | 7.420665 | 2.311918 | -2.917760 |
| 89 | 1 | 0 | 5.830001 | 0.850112 | -3.068622 |
| 90 | 6 | 0 | 8.284308 | 2.999316 | -2.064523 |
| 91 | 1 | 0 | 8.917441 | 3.309881 | -0.017244 |
| 92 | 1 | 0 | 7.457910 | 2.494896 | -4.001552 |
| 93 | 1 | 0 | 9.001958 | 3.723828 | -2.475848 |
| 94 | 6 | 0 | -9.991010 | -2.316466 | -0.562987 |
| 95 | 6 | 0 | -10.400293 | -1.913702 | -1.834007 |
| 96 | 6 | 0 | -10.511736 | -1.688521 | 0.569155 |
| 97 | 6 | 0 | -11.331137 | -0.883889 | -1.973601 |
| 98 | 1 | 0 | -9.990389 | -2.408609 | -2.726356 |
| 99 | 6 | 0 | -11.441997 | -0.658643 | 0.429503 |
| 100 | 1 | 0 | -10.188837 | -2.006218 | 1.571204 |
| 101 | 6 | 0 | -11.852041 | -0.256541 | -0.841948 |
| 102 | 1 | 0 | -11.654024 | -0.566796 | -2.975768 |
| 103 | 1 | 0 | -11.852569 | -0.163546 | 1.321570 |
| 104 | 1 | 0 | -12.585567 | 0.555250 | -0.951793 |
| 105 | 6 | 0 | -9.385162 | -4.705984 | 0.150467 |
| 106 | 6 | 0 | -9.261587 | -4.932745 | 1.521062 |
| 107 | 6 | 0 | -9.863829 | -5.717184 | -0.683541 |
| 108 | 6 | 0 | -9.617601 | -6.170037 | 2.058494 |
| 109 | 1 | 0 | -8.884753 | -4.135946 | 2.178595 |
| 110 | 6 | 0 | -10.219291 | -6.954311 | -0.146233 |
| 111 | 1 | 0 | -9.961122 | -5.538178 | -1.764183 |
| 112 | 6 | 0 | -10.096549 | -7.180652 | 1.224939 |
| 113 | 1 | 0 | -9.520517 | -6.348467 | 3.139179 |
| 114 | 1 | 0 | -10.596562 | -7.751460 | -0.803268 |
| 115 | 1 | 0 | -10.377021 | -8.155950 | 1.648328 |
| 116 | 6 | 0 | -4.828814 | -6.002874 | -1.440075 |
| 117 | 6 | 0 | -5.194327 | -6.338952 | -2.743406 |
| 118 | 6 | 0 | -4.960058 | -6.940521 | -0.414985 |
| 119 | 6 | 0 | -5.690031 | -7.612847 | -3.022585 |
| 120 | 1 | 0 | -5.090669 | -5.600376 | -3.551492 |
| 121 | 6 | 0 | -5.456096 | -8.213936 | -0.694086 |
| 122 | 1 | 0 | -4.671854 | -6.675287 | 0.612579 |
| 123 | 6 | 0 | -5.820750 | -8.550280 | -1.998046 |
| 124 | 1 | 0 | -5.977732 | -7.877761 | -4.050297 |
| 125 | 1 | 0 | -5.559658 | -8.953131 | 0.113589 |
| 126 | 1 | 0 | -6.211592 | -9.554295 | -2.217874 |
| 127 | 6 | 0 | 9.664177 | -4.121769 | 0.562416 |
| 128 | 6 | 0 | 9.943267 | -5.422996 | 0.145117 |
| 129 | 6 | 0 | 10.052568 | -3.698598 | 1.834145 |
| 130 | 6 | 0 | 10.611747 | -6.301205 | 0.998553 |

| | | | | | |
|---|---|---|---|---|---|
| 131 | 1 | 0 | 9.637647 | -5.756643 | -0.857140 |
| 132 | 6 | 0 | 10.720464 | -4.576799 | 2.687500 |
| 133 | 1 | 0 | 9.832355 | -2.672598 | 2.162973 |
| 134 | 6 | 0 | 11.000436 | -5.878085 | 2.269552 |
| 135 | 1 | 0 | 10.832084 | -7.326931 | 0.669190 |
| 136 | 1 | 0 | 11.026685 | -4.243593 | 3.689836 |
| 137 | 1 | 0 | 11.527154 | -6.570175 | 2.942388 |
| 138 | 6 | 0 | 9.830387 | -2.459720 | -1.265517 |
| 139 | 6 | 0 | 10.749943 | -1.533446 | -0.773932 |
| 140 | 6 | 0 | 9.735737 | -2.687201 | -2.639020 |
| 141 | 6 | 0 | 11.575973 | -0.835172 | -1.655158 |
| 142 | 1 | 0 | 10.825013 | -1.354237 | 0.308422 |
| 143 | 6 | 0 | 10.561272 | -1.988711 | -3.520009 |
| 144 | 1 | 0 | 9.010585 | -3.417540 | -3.026387 |
| 145 | 6 | 0 | 11.481741 | -1.062907 | -3.028040 |
| 146 | 1 | 0 | 12.301207 | -0.105252 | -1.267349 |
| 147 | 1 | 0 | 10.486912 | -2.167763 | -4.602545 |
| 148 | 1 | 0 | 12.132581 | -0.512515 | -3.722738 |
| 149 | 6 | 0 | 4.961891 | -5.768547 | 1.369961 |
| 150 | 6 | 0 | 5.007352 | -6.620548 | 0.266671 |
| 151 | 6 | 0 | 5.371717 | -6.226874 | 2.622636 |
| 152 | 6 | 0 | 5.463633 | -7.930607 | 0.415079 |
| 153 | 1 | 0 | 4.684802 | -6.259536 | -0.720686 |
| 154 | 6 | 0 | 5.827430 | -7.536776 | 2.771026 |
| 155 | 1 | 0 | 5.335728 | -5.554937 | 3.492408 |
| 156 | 6 | 0 | 5.873772 | -8.388609 | 1.667105 |
| 157 | 1 | 0 | 5.499816 | -8.602040 | -0.454985 |
| 158 | 1 | 0 | 6.150484 | -7.898293 | 3.758152 |
| 159 | 1 | 0 | 6.233253 | -9.421165 | 1.784287 |
| 160 | 6 | 0 | -0.042086 | 3.860760 | -0.000478 |
| 161 | 6 | 0 | 2.582306 | 2.547176 | -0.284424 |
| 162 | 6 | 0 | 4.106285 | 0.681223 | -0.263443 |
| 163 | 6 | 0 | 2.932831 | -0.058652 | 0.318648 |
| 164 | 7 | 0 | 2.342917 | 3.967086 | -0.461116 |
| 165 | 7 | 0 | 5.458686 | 0.145549 | -0.445427 |
| 166 | 5 | 0 | -0.020971 | 2.229641 | -0.043865 |
| 167 | 5 | 0 | -3.377900 | -1.806906 | -0.552690 |
| 168 | 5 | 0 | 3.518235 | -1.770554 | 0.705761 |
| 169 | 6 | 0 | -4.051547 | 1.923162 | 0.119214 |
| 170 | 1 | 0 | -4.938445 | 2.492004 | 0.305576 |
| 171 | 6 | 0 | -1.655839 | 0.194711 | -0.368564 |
| 172 | 1 | 0 | -0.865079 | -0.469235 | -0.649233 |
| 173 | 6 | 0 | -2.060032 | -5.726682 | -1.341861 |
| 174 | 1 | 0 | -2.548838 | -6.644167 | -1.595221 |
| 175 | 6 | 0 | -0.566086 | -5.557727 | -1.230468 |
| 176 | 1 | 0 | 0.085199 | -6.360332 | -1.507149 |
| 177 | 6 | 0 | -0.036141 | -4.264635 | -0.849925 |
| 178 | 1 | 0 | 0.853595 | -3.893428 | -1.314117 |
| 179 | 6 | 0 | -0.914145 | -2.955281 | -0.571568 |
| 180 | 1 | 0 | -0.560862 | -2.000659 | -0.241737 |
| 181 | 6 | 0 | -6.719654 | -4.129507 | -0.804749 |
| 182 | 1 | 0 | -6.927132 | -5.153600 | -1.035155 |
| 183 | 6 | 0 | -7.246314 | -1.632470 | -0.128886 |
| 184 | 1 | 0 | -7.962257 | -0.885021 | 0.142492 |
| 185 | 6 | 0 | -1.417183 | 6.031385 | 0.466302 |
| 186 | 1 | 0 | -2.347247 | 6.436602 | 0.806422 |
| 187 | 6 | 0 | 1.105193 | 6.011639 | -0.061150 |
| 188 | 1 | 0 | 1.959171 | 6.649711 | -0.153267 |
| 189 | 6 | 0 | 1.626917 | 0.306301 | 0.303533 |
| 190 | 1 | 0 | 0.824072 | -0.331936 | 0.608483 |

| | | | | | |
|---|---|---|---|---|---|
| 191 | 6 | 0 | 3.934380 | 2.082755 | -0.529325 |
| 192 | 1 | 0 | 4.680563 | 2.756886 | -0.894918 |
| 193 | 6 | 0 | 7.392633 | -1.407802 | -0.397065 |
| 194 | 1 | 0 | 8.003367 | -0.532720 | -0.475382 |
| 195 | 6 | 0 | 6.723137 | -3.856091 | 0.397582 |
| 196 | 1 | 0 | 7.054310 | -4.859319 | 0.567161 |
| 197 | 6 | 0 | 2.396558 | -5.118576 | 2.381077 |
| 198 | 1 | 0 | 3.161830 | -5.852894 | 2.239534 |
| 199 | 6 | 0 | 1.164591 | -4.717634 | 3.056675 |
| 200 | 1 | 0 | 0.943263 | -5.725819 | 3.338589 |
| 201 | 6 | 0 | 0.436270 | -3.441285 | 3.021563 |
| 202 | 1 | 0 | -0.630403 | -3.431504 | 3.105303 |
| 203 | 6 | 0 | 1.280783 | -2.454112 | 2.270505 |
| 204 | 1 | 0 | 0.812723 | -1.494277 | 2.203145 |

---------------------------------------------------------------------------------------------------------------------------------

**V-DABNA-core** ($T_1$, $C_1$ symmetry)

E(B3LYP/TZP) = –40.87197480 hartree

| Center Number | Atomic Type | Coordinates (Angstroms) X | Y | Z |
|---|---|---|---|---|
| 1 | N | -1.330523 | -3.955034 | -4.774672 |
| 2 | N | -0.002906 | 2.429358 | 3.624322 |
| 3 | N | 0.000000 | 0.000000 | 7.800724 |
| 4 | N | 0.192666 | 5.298965 | -0.245326 |
| 5 | N | -0.040675 | 8.507881 | -3.866907 |
| 6 | N | 1.330523 | 3.955034 | -4.774672 |
| 7 | C | -0.001959 | -1.206115 | 4.317453 |
| 8 | N | 0.040675 | -8.507881 | -3.866907 |
| 9 | C | 0.288658 | 4.008191 | 0.286504 |
| 10 | C | -0.175229 | -1.381731 | 1.404209 |
| 11 | C | 0.000000 | 0.000000 | 6.418591 |
| 12 | C | -0.741165 | -4.579131 | -2.501700 |
| 13 | C | 0.001959 | 1.206115 | 4.317453 |
| 14 | C | 0.741165 | 4.579131 | -2.501700 |
| 15 | C | -0.894017 | -4.927734 | -3.868857 |
| 16 | C | 0.326045 | 5.599339 | -1.612325 |
| 17 | C | -1.626952 | -2.217772 | -3.053342 |
| 18 | C | 0.578652 | 2.889372 | -0.554957 |
| 19 | C | 0.175229 | 1.381731 | 1.404209 |
| 20 | C | 0.894017 | 4.927734 | -3.868857 |
| 21 | C | 1.753405 | 2.662586 | -4.400167 |
| 22 | C | 0.097766 | 2.547726 | 2.233023 |
| 23 | C | -0.326045 | -5.599339 | -1.612325 |
| 24 | C | -0.187341 | -7.197678 | -3.422605 |
| 25 | C | -1.753405 | -2.662586 | -4.400167 |
| 26 | C | 0.187341 | 7.197678 | -3.422605 |
| 27 | C | 1.626952 | 2.217772 | -3.053342 |
| 28 | C | 0.000000 | 0.000000 | 3.577846 |
| 29 | C | -0.097766 | -2.547726 | 2.233023 |
| 30 | C | -0.288658 | -4.008191 | 0.286504 |
| 31 | C | -0.578652 | -2.889372 | -0.554957 |
| 32 | N | 0.002906 | -2.429358 | 3.624322 |
| 33 | N | -0.192666 | -5.298965 | -0.245326 |
| 34 | B | 0.000000 | 0.000000 | 2.065424 |
| 35 | B | 0.984762 | 3.167992 | -2.018247 |
| 36 | B | -0.984762 | -3.167992 | -2.018247 |

| | | | | |
|---|---|---|---|---|
| 37 | C | 0.105516 | 3.826709 | 1.663857 |
| 38 | H | -0.031610 | 4.686459 | 2.294525 |
| 39 | C | 0.450989 | 1.624689 | 0.046179 |
| 40 | H | 0.581258 | 0.764752 | -0.588502 |
| 41 | C | 2.313549 | 1.821336 | -5.379491 |
| 42 | H | 2.390318 | 2.151044 | -6.404292 |
| 43 | C | 2.787913 | 0.564821 | -5.041301 |
| 44 | H | 3.218012 | -0.064735 | -5.811410 |
| 45 | C | 2.723629 | 0.123554 | -3.719838 |
| 46 | H | 3.109630 | -0.851002 | -3.446288 |
| 47 | C | 2.152856 | 0.941716 | -2.761012 |
| 48 | H | 2.125149 | 0.591796 | -1.739458 |
| 49 | C | 0.603888 | 6.220488 | -4.324998 |
| 50 | H | 0.712277 | 6.481830 | -5.366979 |
| 51 | C | 0.057025 | 6.895216 | -2.065994 |
| 52 | H | -0.250647 | 7.674399 | -1.384614 |
| 53 | C | -0.002268 | 1.208770 | 5.717664 |
| 54 | H | -0.006176 | 2.134097 | 6.273939 |
| 55 | C | 0.002268 | -1.208770 | 5.717664 |
| 56 | H | 0.006176 | -2.134097 | 6.273939 |
| 57 | C | -0.450989 | -1.624689 | 0.046179 |
| 58 | H | -0.581258 | -0.764752 | -0.588502 |
| 59 | C | -0.105516 | -3.826709 | 1.663857 |
| 60 | H | 0.031610 | -4.686459 | 2.294525 |
| 61 | C | -0.057025 | -6.895216 | -2.065994 |
| 62 | H | 0.250647 | -7.674399 | -1.384614 |
| 63 | C | -0.603888 | -6.220488 | -4.324998 |
| 64 | H | -0.712277 | -6.481830 | -5.366979 |
| 65 | C | -2.313549 | -1.821336 | -5.379491 |
| 66 | H | -2.390318 | -2.151044 | -6.404292 |
| 67 | C | -2.787913 | -0.564821 | -5.041301 |
| 68 | H | -3.218012 | 0.064735 | -5.811410 |
| 69 | C | -2.723629 | -0.123554 | -3.719838 |
| 70 | H | -3.109630 | 0.851002 | -3.446288 |
| 71 | C | -2.152856 | -0.941716 | -2.761012 |
| 72 | H | -2.125149 | -0.591796 | -1.739458 |
| 73 | H | -0.675894 | 9.036263 | -3.286059 |
| 74 | C | 1.311980 | -5.332073 | 5.562522 |
| 75 | C | -1.096970 | -5.396382 | 5.594513 |
| 76 | C | 0.128353 | -5.940780 | 5.970708 |
| 77 | H | -1.431311 | -8.822599 | 2.429958 |
| 78 | H | 2.267520 | -5.753063 | 5.850695 |
| 79 | H | 2.759206 | -7.997366 | 2.026083 |
| 80 | H | -2.020770 | -5.867130 | 5.908286 |
| 81 | C | 0.046083 | -3.637416 | 4.411137 |
| 82 | C | 1.272721 | -4.180441 | 4.781780 |
| 83 | C | -1.139875 | -4.245272 | 4.814343 |
| 84 | H | -0.301148 | 8.574046 | -4.840538 |
| 85 | H | 0.160448 | -6.837094 | 6.578244 |
| 86 | H | 2.085722 | 3.811071 | 4.516242 |
| 87 | H | 2.185762 | -3.695546 | 4.460118 |
| 88 | H | 0.002491 | -0.860069 | 8.315474 |
| 89 | H | -0.160448 | 6.837094 | 6.578244 |
| 90 | H | 0.924506 | -9.368938 | 2.974339 |
| 91 | H | -2.185762 | 3.695546 | 4.460118 |
| 92 | H | 2.230171 | -6.086044 | 0.530135 |
| 93 | H | -2.085722 | -3.811071 | 4.516242 |
| 94 | H | 1.943775 | 6.909615 | 0.930152 |
| 95 | C | 1.415984 | 4.320247 | -6.165922 |
| 96 | C | 2.571556 | 4.917340 | -6.663787 |

| | | | | |
|---|---|---|---|---|
| 97 | C | 0.336725 | 4.075112 | -7.010253 |
| 98 | C | 2.645450 | 5.271048 | -8.007626 |
| 99 | H | 3.403490 | 5.100555 | -5.995521 |
| 100 | C | 0.413855 | 4.428963 | -8.354335 |
| 101 | H | -0.552839 | 3.606048 | -6.609258 |
| 102 | C | 1.567039 | 5.027732 | -8.854825 |
| 103 | H | 3.545508 | 5.735037 | -8.392410 |
| 104 | H | -0.426685 | 4.235456 | -9.009802 |
| 105 | H | 1.626077 | 5.302097 | -9.901150 |
| 106 | C | -1.415984 | -4.320247 | -6.165922 |
| 107 | C | -2.571556 | -4.917340 | -6.663787 |
| 108 | C | -0.336725 | -4.075112 | -7.010253 |
| 109 | C | -2.645450 | -5.271048 | -8.007626 |
| 110 | H | -3.403490 | -5.100555 | -5.995521 |
| 111 | C | -0.413855 | -4.428963 | -8.354335 |
| 112 | H | 0.552839 | -3.606048 | -6.609258 |
| 113 | C | -1.567039 | -5.027732 | -8.854825 |
| 114 | H | -3.545508 | -5.735037 | -8.392410 |
| 115 | H | 0.426685 | -4.235456 | -9.009802 |
| 116 | H | -1.626077 | -5.302097 | -9.901150 |
| 117 | H | 0.301148 | -8.574046 | -4.840538 |
| 118 | C | -0.113357 | 6.389779 | 0.645686 |
| 119 | H | -2.230171 | 6.086044 | 0.530135 |
| 120 | C | -1.437898 | 6.691425 | 0.952063 |
| 121 | H | -1.943775 | -6.909615 | 0.930152 |
| 122 | H | -0.924506 | 9.368938 | 2.974339 |
| 123 | H | -0.002491 | 0.860069 | 8.315474 |
| 124 | C | 0.919326 | 7.155312 | 1.179688 |
| 125 | C | -0.697245 | 8.533621 | 2.323084 |
| 126 | H | -2.759206 | 7.997366 | 2.026083 |
| 127 | H | 1.431311 | 8.822599 | 2.429958 |
| 128 | H | 0.675894 | -9.036263 | -3.286059 |
| 129 | C | -1.311980 | 5.332073 | 5.562522 |
| 130 | C | 1.096970 | 5.396382 | 5.594513 |
| 131 | C | -0.128353 | 5.940780 | 5.970708 |
| 132 | C | -1.728064 | 7.763451 | 1.790430 |
| 133 | H | -2.267520 | 5.753063 | 5.850695 |
| 134 | C | 0.625884 | 8.227101 | 2.017624 |
| 135 | H | 2.020770 | 5.867130 | 5.908286 |
| 136 | C | -0.046083 | 3.637416 | 4.411137 |
| 137 | C | -1.272721 | 4.180441 | 4.781780 |
| 138 | C | 1.139875 | 4.245272 | 4.814343 |
| 139 | C | 0.113357 | -6.389779 | 0.645686 |
| 140 | C | -0.919326 | -7.155312 | 1.179688 |
| 141 | C | 1.437898 | -6.691425 | 0.952063 |
| 142 | C | -0.625884 | -8.227101 | 2.017624 |
| 143 | C | 1.728064 | -7.763451 | 1.790430 |
| 144 | C | 0.697245 | -8.533621 | 2.323084 |

**5-F** ($S_0$, $C_1$ symmetry)

E(B3LYP/6-31G(d)) = –5683.122630 hartree

| Center | Atomic | Atomic | Coordinates (Angstroms) | | |
|---|---|---|---|---|---|
| Number | Number | Type | X | Y | Z |
| 1 | 6 | 0 | -9.459505 | -1.308193 | -0.208089 |
| 2 | 6 | 0 | -9.915738 | 1.329881 | 0.649464 |

| | | | | | |
|---|---|---|---|---|---|
| 3 | 1 | 0 | -9.307282 | -2.318724 | -0.559808 |
| 4 | 6 | 0 | -8.616332 | 0.770637 | 0.791195 |
| 5 | 6 | 0 | -8.408575 | -0.564419 | 0.345588 |
| 6 | 6 | 0 | -6.084329 | 0.935341 | 1.266271 |
| 7 | 6 | 0 | -4.927492 | 1.711972 | 1.102604 |
| 8 | 6 | 0 | -5.985373 | -0.423777 | 0.837544 |
| 9 | 1 | 0 | -4.992635 | 2.763011 | 1.353759 |
| 10 | 6 | 0 | -4.748728 | -0.953608 | 0.431183 |
| 11 | 1 | 0 | -4.682909 | -1.997701 | 0.164364 |
| 12 | 6 | 0 | -3.689061 | 1.247416 | 0.637193 |
| 13 | 6 | 0 | -3.600603 | -0.149697 | 0.361156 |
| 14 | 6 | 0 | -1.114855 | 1.292739 | -0.481226 |
| 15 | 6 | 0 | 0.113663 | 1.987638 | -0.391473 |
| 16 | 6 | 0 | -1.151504 | -0.047720 | -0.010960 |
| 17 | 6 | 0 | 0.014013 | -0.692095 | 0.432953 |
| 18 | 1 | 0 | -0.024204 | -1.722569 | 0.751306 |
| 19 | 6 | 0 | -11.015267 | 3.475373 | 0.291698 |
| 20 | 6 | 0 | -10.472347 | 4.261587 | -0.727181 |
| 21 | 6 | 0 | -12.391243 | 3.498454 | 0.537659 |
| 22 | 6 | 0 | -11.305912 | 5.072006 | -1.499380 |
| 23 | 1 | 0 | -9.401464 | 4.232353 | -0.904512 |
| 24 | 6 | 0 | -13.221584 | 4.310286 | -0.236275 |
| 25 | 6 | 0 | -12.680349 | 5.097759 | -1.254930 |
| 26 | 1 | 0 | -10.880970 | 5.682116 | -2.291584 |
| 27 | 1 | 0 | -14.290752 | 4.326634 | -0.043456 |
| 28 | 1 | 0 | -13.328371 | 5.728507 | -1.857192 |
| 29 | 6 | 0 | -9.634562 | 3.168352 | 2.289892 |
| 30 | 6 | 0 | -10.053463 | 4.450023 | 2.713043 |
| 31 | 6 | 0 | -8.714339 | 2.422492 | 3.080731 |
| 32 | 6 | 0 | -9.624404 | 4.965485 | 3.926727 |
| 33 | 6 | 0 | -8.345436 | 2.977412 | 4.327205 |
| 34 | 6 | 0 | -8.782107 | 4.220575 | 4.759473 |
| 35 | 1 | 0 | -9.967740 | 5.950635 | 4.233042 |
| 36 | 1 | 0 | -7.710518 | 2.393998 | 4.984490 |
| 37 | 1 | 0 | -8.474684 | 4.609087 | 5.726278 |
| 38 | 1 | 0 | -10.727159 | 5.036138 | 2.101225 |
| 39 | 1 | 0 | -12.798258 | 2.882143 | 1.333779 |
| 40 | 7 | 0 | -10.149024 | 2.642153 | 1.087429 |
| 41 | 6 | 0 | 1.229639 | 0.009395 | 0.461955 |
| 42 | 6 | 0 | 1.293874 | 1.381527 | 0.097982 |
| 43 | 6 | 0 | 3.680479 | -0.025842 | 0.091623 |
| 44 | 6 | 0 | 4.801359 | -0.775012 | -0.296843 |
| 45 | 6 | 0 | 3.820850 | 1.358711 | 0.406290 |
| 46 | 1 | 0 | 4.696052 | -1.834374 | -0.476852 |
| 47 | 6 | 0 | 5.084140 | 1.923436 | 0.178785 |
| 48 | 1 | 0 | 5.189609 | 2.982147 | 0.378774 |
| 49 | 6 | 0 | 6.062768 | -0.174113 | -0.447717 |
| 50 | 6 | 0 | 6.217907 | 1.236031 | -0.279959 |
| 51 | 6 | 0 | 8.516750 | -0.430212 | -0.240973 |
| 52 | 6 | 0 | 9.593671 | -1.250692 | 0.121633 |
| 53 | 6 | 0 | 8.731018 | 0.918076 | -0.642822 |
| 54 | 1 | 0 | 9.444373 | -2.285995 | 0.394049 |
| 55 | 6 | 0 | 10.062734 | 1.416072 | -0.634207 |
| 56 | 7 | 0 | 10.300726 | 2.744080 | -1.018963 |
| 57 | 6 | 0 | 9.991132 | 4.691884 | -2.442899 |
| 58 | 6 | 0 | 9.343294 | 5.340003 | -3.483630 |
| 59 | 6 | 0 | 9.569790 | 3.409755 | -2.023697 |
| 60 | 6 | 0 | 8.271149 | 4.730906 | -4.144987 |
| 61 | 1 | 0 | 9.693030 | 6.321773 | -3.793534 |
| 62 | 6 | 0 | 8.432239 | 2.800588 | -2.626979 |

| | | | | | |
|---|---|---|---|---|---|
| 63 | 6 | 0 | 7.838147 | 3.487425 | -3.710024 |
| 64 | 1 | 0 | 7.786387 | 5.222888 | -4.983501 |
| 65 | 1 | 0 | 7.022148 | 3.009038 | -4.239757 |
| 66 | 6 | 0 | 11.392591 | 3.443768 | -0.389808 |
| 67 | 6 | 0 | 11.157511 | 4.193753 | 0.765172 |
| 68 | 6 | 0 | 12.679919 | 3.372126 | -0.929503 |
| 69 | 6 | 0 | 12.210586 | 4.873460 | 1.379244 |
| 70 | 1 | 0 | 10.151315 | 4.239240 | 1.171057 |
| 71 | 6 | 0 | 13.730723 | 4.051701 | -0.311847 |
| 72 | 1 | 0 | 12.847139 | 2.784080 | -1.826876 |
| 73 | 6 | 0 | 13.497572 | 4.803139 | 0.842039 |
| 74 | 1 | 0 | 12.024851 | 5.456539 | 2.277052 |
| 75 | 1 | 0 | 14.731055 | 3.992384 | -0.731538 |
| 76 | 1 | 0 | 14.317000 | 5.331338 | 1.321709 |
| 77 | 1 | 0 | 10.836419 | 5.173346 | -1.967981 |
| 78 | 6 | 0 | 10.890078 | -0.721657 | 0.138029 |
| 79 | 6 | 0 | 11.836707 | -2.433511 | 1.632898 |
| 80 | 6 | 0 | 12.313568 | -3.752017 | 1.561619 |
| 81 | 6 | 0 | 11.224120 | -1.994503 | 2.817803 |
| 82 | 6 | 0 | 12.186790 | -4.605748 | 2.655898 |
| 83 | 1 | 0 | 12.782769 | -4.099394 | 0.646609 |
| 84 | 6 | 0 | 11.085780 | -2.860649 | 3.900723 |
| 85 | 1 | 0 | 10.864451 | -0.972404 | 2.881620 |
| 86 | 6 | 0 | 11.568910 | -4.169233 | 3.829679 |
| 87 | 1 | 0 | 12.562124 | -5.623323 | 2.583196 |
| 88 | 1 | 0 | 10.611109 | -2.503423 | 4.811056 |
| 89 | 1 | 0 | 11.466727 | -4.839337 | 4.678656 |
| 90 | 6 | 0 | 13.205885 | -1.503643 | -0.186061 |
| 91 | 6 | 0 | 14.430795 | -1.560205 | 0.498730 |
| 92 | 6 | 0 | 13.215669 | -1.405030 | -1.587374 |
| 93 | 6 | 0 | 15.633183 | -1.528121 | -0.205233 |
| 94 | 1 | 0 | 14.432803 | -1.629205 | 1.581782 |
| 95 | 6 | 0 | 14.423178 | -1.355453 | -2.280652 |
| 96 | 1 | 0 | 12.273390 | -1.368064 | -2.124351 |
| 97 | 6 | 0 | 15.639583 | -1.421191 | -1.597328 |
| 98 | 1 | 0 | 16.570994 | -1.572842 | 0.342625 |
| 99 | 1 | 0 | 14.410670 | -1.280730 | -3.365121 |
| 100 | 1 | 0 | 16.578867 | -1.390250 | -2.142275 |
| 101 | 7 | 0 | 11.977212 | -1.552216 | 0.525902 |
| 102 | 7 | 0 | 7.212355 | -0.954081 | -0.235303 |
| 103 | 7 | 0 | 2.428577 | -0.666097 | 0.186103 |
| 104 | 7 | 0 | -2.373478 | -0.738688 | -0.003857 |
| 105 | 7 | 0 | -7.130696 | -1.141553 | 0.452313 |
| 106 | 6 | 0 | 7.052981 | -2.364902 | 0.041702 |
| 107 | 6 | 0 | 6.898814 | -2.801259 | 1.370041 |
| 108 | 6 | 0 | 7.068233 | -3.271668 | -1.029606 |
| 109 | 6 | 0 | 6.758891 | -4.171476 | 1.603326 |
| 110 | 6 | 0 | 6.924936 | -4.634732 | -0.745789 |
| 111 | 6 | 0 | 6.772438 | -5.104930 | 0.560572 |
| 112 | 1 | 0 | 6.640440 | -4.517775 | 2.628131 |
| 113 | 1 | 0 | 6.935219 | -5.345003 | -1.569997 |
| 114 | 6 | 0 | 2.368405 | -2.093403 | -0.040644 |
| 115 | 6 | 0 | 2.165216 | -2.581674 | -1.343518 |
| 116 | 6 | 0 | 2.516114 | -2.960878 | 1.053305 |
| 117 | 6 | 0 | 2.112290 | -3.965666 | -1.528503 |
| 118 | 6 | 0 | 2.454558 | -4.338725 | 0.817863 |
| 119 | 6 | 0 | 2.251717 | -4.860271 | -0.462024 |
| 120 | 1 | 0 | 1.957839 | -4.354067 | -2.533294 |
| 121 | 1 | 0 | 2.568797 | -5.019596 | 1.658932 |
| 122 | 6 | 0 | -2.361586 | -2.144709 | -0.343124 |

| | | | | | |
|---|---|---|---|---|---|
| 123 | 6 | 0 | -2.542650 | -2.523523 | -1.683676 |
| 124 | 6 | 0 | -2.161606 | -3.098856 | 0.669006 |
| 125 | 6 | 0 | -2.520916 | -3.886876 | -1.993979 |
| 126 | 6 | 0 | -2.146417 | -4.449750 | 0.309354 |
| 127 | 6 | 0 | -2.327673 | -4.864356 | -1.013560 |
| 128 | 1 | 0 | -2.656437 | -4.190433 | -3.030166 |
| 129 | 1 | 0 | -1.987425 | -5.196005 | 1.085129 |
| 130 | 6 | 0 | -6.991354 | -2.536596 | 0.096155 |
| 131 | 6 | 0 | -6.701108 | -2.889746 | -1.232306 |
| 132 | 6 | 0 | -7.165275 | -3.512347 | 1.092505 |
| 133 | 6 | 0 | -6.590565 | -4.248673 | -1.545788 |
| 134 | 6 | 0 | -7.044543 | -4.857136 | 0.731135 |
| 135 | 6 | 0 | -6.764103 | -5.246507 | -0.582465 |
| 136 | 1 | 0 | -6.364984 | -4.531963 | -2.571870 |
| 137 | 1 | 0 | -7.177603 | -5.618936 | 1.496712 |
| 138 | 6 | 0 | -10.726693 | -0.725294 | -0.335191 |
| 139 | 6 | 0 | -11.950908 | -2.850207 | -0.561606 |
| 140 | 6 | 0 | -11.826620 | -3.268620 | 0.773182 |
| 141 | 6 | 0 | -12.245846 | -3.804339 | -1.548561 |
| 142 | 6 | 0 | -11.987243 | -4.611877 | 1.106905 |
| 143 | 1 | 0 | -11.606304 | -2.534440 | 1.541556 |
| 144 | 6 | 0 | -12.421696 | -5.142889 | -1.202823 |
| 145 | 1 | 0 | -12.336619 | -3.489222 | -2.583238 |
| 146 | 6 | 0 | -12.290166 | -5.557010 | 0.124288 |
| 147 | 1 | 0 | -11.889372 | -4.917558 | 2.145466 |
| 148 | 1 | 0 | -12.650155 | -5.867538 | -1.980222 |
| 149 | 1 | 0 | -12.422604 | -6.602176 | 0.389502 |
| 150 | 6 | 0 | -12.687755 | -0.871265 | -1.821590 |
| 151 | 6 | 0 | -12.213877 | 0.005635 | -2.811040 |
| 152 | 6 | 0 | -14.064274 | -1.141402 | -1.756118 |
| 153 | 6 | 0 | -13.100412 | 0.603204 | -3.704427 |
| 154 | 1 | 0 | -11.150482 | 0.213323 | -2.871848 |
| 155 | 6 | 0 | -14.940897 | -0.553027 | -2.665892 |
| 156 | 1 | 0 | -14.439472 | -1.812778 | -0.990382 |
| 157 | 6 | 0 | -14.467840 | 0.325698 | -3.642458 |
| 158 | 1 | 0 | -12.715215 | 1.278882 | -4.463879 |
| 159 | 1 | 0 | -16.003155 | -0.774059 | -2.599584 |
| 160 | 1 | 0 | -15.155008 | 0.787575 | -4.345759 |
| 161 | 7 | 0 | -11.787251 | -1.480503 | -0.905984 |
| 162 | 6 | 0 | -10.963922 | 0.585960 | 0.086779 |
| 163 | 1 | 0 | -11.958429 | 0.997147 | -0.009573 |
| 164 | 6 | 0 | 11.132219 | 0.607183 | -0.221320 |
| 165 | 1 | 0 | 12.143420 | 0.985869 | -0.182955 |
| 166 | 6 | 0 | 1.688171 | 4.340227 | -1.063680 |
| 167 | 6 | 0 | 2.614793 | 5.406329 | -1.074168 |
| 168 | 6 | 0 | 0.708596 | 4.337781 | -0.036499 |
| 169 | 6 | 0 | 2.590149 | 6.426621 | -0.134185 |
| 170 | 1 | 0 | 3.399363 | 5.402621 | -1.823828 |
| 171 | 6 | 0 | 1.633933 | 6.384777 | 0.886761 |
| 172 | 1 | 0 | 3.321800 | 7.229070 | -0.171560 |
| 173 | 1 | 0 | 1.623524 | 7.147802 | 1.661031 |
| 174 | 6 | 0 | -2.685488 | 3.452791 | -0.157734 |
| 175 | 6 | 0 | -1.516282 | 3.494521 | 0.645737 |
| 176 | 6 | 0 | -3.917193 | 3.734978 | 0.473638 |
| 177 | 6 | 0 | -4.011490 | 4.039766 | 1.823941 |
| 178 | 1 | 0 | -4.817327 | 3.753018 | -0.132166 |
| 179 | 6 | 0 | -2.840585 | 4.105556 | 2.587587 |
| 180 | 1 | 0 | -4.976315 | 4.255142 | 2.274911 |
| 181 | 1 | 0 | -2.884317 | 4.387046 | 3.636712 |
| 182 | 7 | 0 | -0.230484 | 3.269698 | 0.070820 |

| | | | | | |
|---|---|---|---|---|---|
| 183 | 6 | 0 | 0.711675 | 5.349286 | 0.944233 |
| 184 | 1 | 0 | 0.006405 | 5.308832 | 1.765819 |
| 185 | 6 | 0 | -1.606789 | 3.848185 | 2.006441 |
| 186 | 1 | 0 | -0.706571 | 3.945374 | 2.601599 |
| 187 | 1 | 0 | -8.320486 | 1.485376 | 2.746675 |
| 188 | 1 | 0 | -6.983690 | 1.335600 | 1.685609 |
| 189 | 1 | 0 | -7.817206 | 1.337806 | 1.220866 |
| 190 | 1 | 0 | -2.635933 | 3.216963 | -1.200245 |
| 191 | 1 | 0 | -1.986624 | 1.760199 | -0.889147 |
| 192 | 1 | 0 | -2.856257 | 1.904395 | 0.496779 |
| 193 | 1 | 0 | 1.723309 | 3.565355 | -1.800727 |
| 194 | 1 | 0 | 3.006879 | 1.934559 | 0.794556 |
| 195 | 1 | 0 | 2.203729 | 1.936870 | 0.191037 |
| 196 | 1 | 0 | 8.045498 | 1.866499 | -2.276539 |
| 197 | 1 | 0 | 7.913185 | 1.539364 | -0.942907 |
| 198 | 1 | 0 | 7.140052 | 1.736151 | -0.490742 |
| 199 | 1 | 0 | -6.705395 | -6.281968 | -0.845665 |
| 200 | 1 | 0 | -2.337141 | -5.904157 | -1.265800 |
| 201 | 1 | 0 | 2.188996 | -5.916059 | -0.624124 |
| 202 | 1 | 0 | 6.677082 | -6.150895 | 0.764936 |
| 203 | 9 | 0 | -6.537832 | -1.946601 | -2.184313 |
| 204 | 9 | 0 | -7.445171 | -3.160054 | 2.365316 |
| 205 | 9 | 0 | -1.983624 | -2.723032 | 1.953366 |
| 206 | 9 | 0 | -2.727639 | -1.597127 | -2.648077 |
| 207 | 9 | 0 | 2.025826 | -1.738021 | -2.388181 |
| 208 | 9 | 0 | 2.713544 | -2.480561 | 2.299426 |
| 209 | 9 | 0 | 7.222728 | -2.843193 | -2.300449 |
| 210 | 9 | 0 | 6.895390 | -1.921785 | 2.394255 |

**V-DABNA-F** ($S_0$, $C_1$ symmetry)

E(B3LYP/6-31G(d)) = –5752.473683 hartree

| Center Number | Atomic Number | Atomic Type | Coordinates (Angstroms) X | Y | Z |
|---|---|---|---|---|---|
| 1 | 7 | 0 | -4.641448 | -4.028593 | -1.896916 |
| 2 | 7 | 0 | 2.630305 | 3.521026 | -0.787192 |
| 3 | 7 | 0 | 0.645603 | 7.904567 | -0.058001 |
| 4 | 7 | 0 | 5.254488 | -0.517904 | -0.515288 |
| 5 | 7 | 0 | 8.450662 | -3.975700 | 0.697624 |
| 6 | 7 | 0 | 3.685915 | -4.822165 | 1.155806 |
| 7 | 6 | 0 | -0.843169 | 4.503207 | 0.124630 |
| 8 | 7 | 0 | -8.748638 | -3.131027 | 0.548634 |
| 9 | 6 | 0 | 3.977730 | 0.074287 | -0.493975 |
| 10 | 6 | 0 | -1.327036 | 1.658726 | -0.274694 |
| 11 | 6 | 0 | 0.518236 | 6.487864 | -0.106531 |
| 12 | 6 | 0 | -4.939951 | -1.860240 | -0.823438 |
| 13 | 6 | 0 | 1.511840 | 4.313699 | -0.474497 |
| 14 | 6 | 0 | 4.430836 | -2.664014 | 0.301652 |
| 15 | 6 | 0 | -5.432695 | -3.157285 | -1.132824 |
| 16 | 6 | 0 | 5.518566 | -1.815010 | -0.044071 |
| 17 | 6 | 0 | -2.864857 | -2.363252 | -2.284389 |
| 18 | 6 | 0 | 2.815866 | -0.698475 | -0.188736 |
| 19 | 6 | 0 | 1.453377 | 1.392842 | -0.381929 |
| 20 | 6 | 0 | 4.733353 | -3.963304 | 0.795949 |
| 21 | 6 | 0 | 2.329133 | -4.554644 | 0.882320 |
| 22 | 6 | 0 | 2.652013 | 2.119541 | -0.648409 |

| | | | | | |
|---|---|---|---|---|---|
| 23 | 6 | 0 | -5.769852 | -0.993152 | -0.060308 |
| 24 | 6 | 0 | -7.473407 | -2.704356 | 0.083858 |
| 25 | 6 | 0 | -3.435363 | -3.647395 | -2.519432 |
| 26 | 6 | 0 | 7.103003 | -3.537290 | 0.566703 |
| 27 | 6 | 0 | 1.925971 | -3.307475 | 0.324205 |
| 28 | 6 | 0 | 1.797190 | 8.494047 | 0.529457 |
| 29 | 6 | 0 | 2.413943 | 9.606953 | -0.065238 |
| 30 | 6 | 0 | 2.335518 | 7.974596 | 1.718488 |
| 31 | 6 | 0 | 3.534510 | 10.189412 | 0.524056 |
| 32 | 1 | 0 | 2.010050 | 10.009753 | -0.988578 |
| 33 | 6 | 0 | 3.466709 | 8.553113 | 2.290446 |
| 34 | 1 | 0 | 1.860308 | 7.119147 | 2.187759 |
| 35 | 6 | 0 | 4.071461 | 9.666126 | 1.702055 |
| 36 | 1 | 0 | 3.998005 | 11.050232 | 0.048800 |
| 37 | 1 | 0 | 3.867298 | 8.138825 | 3.212294 |
| 38 | 1 | 0 | 4.948400 | 10.119582 | 2.155517 |
| 39 | 6 | 0 | -0.380216 | 8.730528 | -0.590921 |
| 40 | 6 | 0 | -0.784547 | 9.893906 | 0.084137 |
| 41 | 6 | 0 | -1.003351 | 8.398906 | -1.805378 |
| 42 | 6 | 0 | -1.780299 | 10.708491 | -0.451582 |
| 43 | 1 | 0 | -0.313647 | 10.153430 | 1.026903 |
| 44 | 6 | 0 | -2.010295 | 9.210448 | -2.323896 |
| 45 | 1 | 0 | -0.690970 | 7.505120 | -2.335729 |
| 46 | 6 | 0 | -2.402972 | 10.372185 | -1.655289 |
| 47 | 1 | 0 | -2.079699 | 11.605061 | 0.085297 |
| 48 | 1 | 0 | -2.479379 | 8.938904 | -3.266262 |
| 49 | 1 | 0 | -3.182846 | 11.006634 | -2.067030 |
| 50 | 6 | 0 | 6.367825 | 0.263964 | -0.991406 |
| 51 | 6 | 0 | 7.155212 | 0.996192 | -0.098121 |
| 52 | 6 | 0 | 6.663185 | 0.275572 | -2.356821 |
| 53 | 6 | 0 | 8.238872 | 1.735892 | -0.572962 |
| 54 | 6 | 0 | 7.745654 | 1.019344 | -2.828355 |
| 55 | 6 | 0 | 8.535065 | 1.749079 | -1.937484 |
| 56 | 1 | 0 | 8.852431 | 2.299832 | 0.124060 |
| 57 | 1 | 0 | 7.973203 | 1.025556 | -3.890828 |
| 58 | 1 | 0 | 9.380078 | 2.324930 | -2.304921 |
| 59 | 6 | 0 | 3.845119 | 4.191968 | -1.175656 |
| 60 | 6 | 0 | 4.129655 | 4.370645 | -2.531593 |
| 61 | 6 | 0 | 4.732349 | 4.666892 | -0.205234 |
| 62 | 6 | 0 | 5.302929 | 5.021322 | -2.916216 |
| 63 | 6 | 0 | 5.902407 | 5.320248 | -0.593608 |
| 64 | 6 | 0 | 6.189934 | 5.496836 | -1.948657 |
| 65 | 1 | 0 | 5.521849 | 5.157933 | -3.971695 |
| 66 | 1 | 0 | 6.587627 | 5.692495 | 0.162890 |
| 67 | 1 | 0 | 7.101532 | 6.005928 | -2.249392 |
| 68 | 6 | 0 | -3.148282 | 4.744363 | 0.885653 |
| 69 | 6 | 0 | -3.309448 | 4.937958 | 2.259761 |
| 70 | 6 | 0 | -4.012005 | 5.371761 | -0.016765 |
| 71 | 6 | 0 | -4.335249 | 5.758771 | 2.730710 |
| 72 | 6 | 0 | -5.035795 | 6.192289 | 0.457887 |
| 73 | 6 | 0 | -5.198947 | 6.386458 | 1.831129 |
| 74 | 1 | 0 | -4.458054 | 5.907913 | 3.799964 |
| 75 | 1 | 0 | -5.703378 | 6.681935 | -0.245679 |
| 76 | 1 | 0 | -5.995996 | 7.026947 | 2.198758 |
| 77 | 6 | 0 | -6.234483 | 1.194916 | 0.906101 |
| 78 | 6 | 0 | -7.114348 | 1.987251 | 0.164385 |
| 79 | 6 | 0 | -6.243754 | 1.254253 | 2.302317 |
| 80 | 6 | 0 | -8.003064 | 2.839536 | 0.821206 |
| 81 | 6 | 0 | -7.133990 | 2.107341 | 2.955186 |
| 82 | 6 | 0 | -8.014130 | 2.900526 | 2.216065 |

| | | | | | |
|---|---|---|---|---|---|
| 83 | 1 | 0 | -8.687177 | 3.454306 | 0.242672 |
| 84 | 1 | 0 | -7.141505 | 2.149577 | 4.040777 |
| 85 | 1 | 0 | -8.708118 | 3.563149 | 2.725914 |
| 86 | 6 | 0 | 9.438487 | -3.091989 | 1.208768 |
| 87 | 6 | 0 | 9.148451 | -2.252677 | 2.297310 |
| 88 | 6 | 0 | 10.721005 | -3.044350 | 0.638219 |
| 89 | 6 | 0 | 10.117573 | -1.383154 | 2.793388 |
| 90 | 1 | 0 | 8.162097 | -2.289751 | 2.748286 |
| 91 | 6 | 0 | 11.688910 | -2.183661 | 1.152623 |
| 92 | 1 | 0 | 10.951624 | -3.684391 | -0.207379 |
| 93 | 6 | 0 | 11.394746 | -1.344528 | 2.229299 |
| 94 | 1 | 0 | 9.875258 | -0.743138 | 3.638144 |
| 95 | 1 | 0 | 12.675742 | -2.160737 | 0.697254 |
| 96 | 1 | 0 | 12.150285 | -0.670949 | 2.623942 |
| 97 | 6 | 0 | 8.806878 | -5.299666 | 0.325007 |
| 98 | 6 | 0 | 8.286176 | -5.873468 | -0.846689 |
| 99 | 6 | 0 | 9.687493 | -6.054328 | 1.117078 |
| 100 | 6 | 0 | 8.635725 | -7.172490 | -1.209295 |
| 101 | 1 | 0 | 7.610214 | -5.293823 | -1.467018 |
| 102 | 6 | 0 | 10.044656 | -7.346658 | 0.736792 |
| 103 | 1 | 0 | 10.087813 | -5.621907 | 2.028527 |
| 104 | 6 | 0 | 9.519902 | -7.916697 | -0.424903 |
| 105 | 1 | 0 | 8.224616 | -7.598850 | -2.120922 |
| 106 | 1 | 0 | 10.727546 | -7.915395 | 1.362830 |
| 107 | 1 | 0 | 9.796158 | -8.926521 | -0.715082 |
| 108 | 6 | 0 | 4.025535 | -6.083948 | 1.763304 |
| 109 | 6 | 0 | 4.093005 | -6.186442 | 3.154965 |
| 110 | 6 | 0 | 4.288672 | -7.200669 | 0.964857 |
| 111 | 6 | 0 | 4.421912 | -7.406836 | 3.747232 |
| 112 | 1 | 0 | 3.885751 | -5.309490 | 3.760956 |
| 113 | 6 | 0 | 4.619074 | -8.418571 | 1.560286 |
| 114 | 1 | 0 | 4.234145 | -7.104395 | -0.115298 |
| 115 | 6 | 0 | 4.685547 | -8.523499 | 2.951391 |
| 116 | 1 | 0 | 4.473051 | -7.483797 | 4.829879 |
| 117 | 1 | 0 | 4.826309 | -9.284134 | 0.937182 |
| 118 | 1 | 0 | 4.943503 | -9.472433 | 3.413584 |
| 119 | 6 | 0 | -9.602693 | -3.877958 | -0.307082 |
| 120 | 6 | 0 | -10.315488 | -4.985158 | 0.180849 |
| 121 | 6 | 0 | -9.749286 | -3.516700 | -1.656476 |
| 122 | 6 | 0 | -11.160211 | -5.705301 | -0.661918 |
| 123 | 1 | 0 | -10.202523 | -5.275078 | 1.220638 |
| 124 | 6 | 0 | -10.582622 | -4.253460 | -2.495505 |
| 125 | 1 | 0 | -9.206594 | -2.657655 | -2.037797 |
| 126 | 6 | 0 | -11.296943 | -5.348952 | -2.005104 |
| 127 | 1 | 0 | -11.703596 | -6.559690 | -0.266415 |
| 128 | 1 | 0 | -10.684935 | -3.957931 | -3.536673 |
| 129 | 1 | 0 | -11.951656 | -5.916420 | -2.660524 |
| 130 | 6 | 0 | -9.163705 | -2.822491 | 1.871931 |
| 131 | 6 | 0 | -10.486242 | -2.428246 | 2.133147 |
| 132 | 6 | 0 | -8.257872 | -2.911148 | 2.941902 |
| 133 | 6 | 0 | -10.890749 | -2.140554 | 3.435310 |
| 134 | 1 | 0 | -11.190917 | -2.349796 | 1.311423 |
| 135 | 6 | 0 | -8.667376 | -2.604022 | 4.237770 |
| 136 | 1 | 0 | -7.236123 | -3.221597 | 2.748941 |
| 137 | 6 | 0 | -9.985692 | -2.221664 | 4.495592 |
| 138 | 1 | 0 | -11.918522 | -1.837016 | 3.617723 |
| 139 | 1 | 0 | -7.952292 | -2.679828 | 5.053045 |
| 140 | 1 | 0 | -10.303261 | -1.990524 | 5.508490 |
| 141 | 6 | 0 | -5.133427 | -5.364766 | -2.121184 |
| 142 | 6 | 0 | -4.763560 | -6.394773 | -1.252892 |

| | | | | | |
|---|---|---|---|---|---|
| 143 | 6 | 0 | -5.978468 | -5.629824 | -3.202615 |
| 144 | 6 | 0 | -5.237797 | -7.689782 | -1.468009 |
| 145 | 1 | 0 | -4.107710 | -6.173127 | -0.416248 |
| 146 | 6 | 0 | -6.451688 | -6.925389 | -3.413899 |
| 147 | 1 | 0 | -6.258490 | -4.818724 | -3.868008 |
| 148 | 6 | 0 | -6.081853 | -7.956695 | -2.547847 |
| 149 | 1 | 0 | -4.948292 | -8.488746 | -0.790942 |
| 150 | 1 | 0 | -7.110144 | -7.127849 | -4.254008 |
| 151 | 1 | 0 | -6.451664 | -8.964873 | -2.713252 |
| 152 | 6 | 0 | 0.268278 | 3.683060 | -0.205911 |
| 153 | 6 | 0 | -2.361397 | 2.545745 | 0.150950 |
| 154 | 6 | 0 | -4.018769 | 0.763588 | -0.038037 |
| 155 | 6 | 0 | -3.068761 | -0.082511 | -0.686352 |
| 156 | 7 | 0 | -2.083179 | 3.900625 | 0.405542 |
| 157 | 7 | 0 | -5.322775 | 0.308188 | 0.227991 |
| 158 | 5 | 0 | 0.126008 | 2.174499 | -0.292777 |
| 159 | 5 | 0 | 2.994171 | -2.202966 | 0.130974 |
| 160 | 5 | 0 | -3.562243 | -1.419627 | -1.275418 |
| 161 | 6 | 0 | 3.880342 | 1.452224 | -0.734702 |
| 162 | 1 | 0 | 4.776770 | 2.017857 | -0.932691 |
| 163 | 6 | 0 | 1.606226 | 0.017085 | -0.142251 |
| 164 | 1 | 0 | 0.717291 | -0.525183 | 0.154150 |
| 165 | 6 | 0 | 1.368460 | -5.554801 | 1.152011 |
| 166 | 1 | 0 | 1.665665 | -6.491385 | 1.606186 |
| 167 | 6 | 0 | 0.037610 | -5.360326 | 0.814680 |
| 168 | 1 | 0 | -0.680057 | -6.150590 | 1.022616 |
| 169 | 6 | 0 | -0.373128 | -4.178456 | 0.189803 |
| 170 | 1 | 0 | -1.404052 | -4.038595 | -0.119979 |
| 171 | 6 | 0 | 0.566060 | -3.184451 | -0.038047 |
| 172 | 1 | 0 | 0.244898 | -2.283333 | -0.545011 |
| 173 | 6 | 0 | 6.062841 | -4.385003 | 0.953787 |
| 174 | 1 | 0 | 6.302189 | -5.357423 | 1.359330 |
| 175 | 6 | 0 | 6.843117 | -2.261426 | 0.054926 |
| 176 | 1 | 0 | 7.673960 | -1.639158 | -0.244063 |
| 177 | 6 | 0 | 1.626826 | 5.711116 | -0.458365 |
| 178 | 1 | 0 | 2.556435 | 6.204946 | -0.702084 |
| 179 | 6 | 0 | -0.711604 | 5.896557 | 0.201794 |
| 180 | 1 | 0 | -1.541922 | 6.528288 | 0.483229 |
| 181 | 6 | 0 | -1.747970 | 0.394876 | -0.712789 |
| 182 | 1 | 0 | -0.986294 | -0.263114 | -1.112672 |
| 183 | 6 | 0 | -3.669371 | 2.072547 | 0.326637 |
| 184 | 1 | 0 | -4.420812 | 2.729856 | 0.734868 |
| 185 | 6 | 0 | -7.034665 | -1.407790 | 0.376703 |
| 186 | 1 | 0 | -7.682564 | -0.750417 | 0.938377 |
| 187 | 6 | 0 | -6.682789 | -3.584618 | -0.659806 |
| 188 | 1 | 0 | -7.051168 | -4.580688 | -0.858692 |
| 189 | 6 | 0 | -2.799569 | -4.553216 | -3.398088 |
| 190 | 1 | 0 | -3.212997 | -5.540331 | -3.560472 |
| 191 | 6 | 0 | -1.649817 | -4.182796 | -4.079080 |
| 192 | 1 | 0 | -1.183121 | -4.895994 | -4.754046 |
| 193 | 6 | 0 | -1.107860 | -2.902847 | -3.920133 |
| 194 | 1 | 0 | -0.224754 | -2.602928 | -4.477029 |
| 195 | 6 | 0 | -1.716319 | -2.024950 | -3.035716 |
| 196 | 1 | 0 | -1.308456 | -1.025650 | -2.931555 |
| 197 | 9 | 0 | -2.463826 | 4.323909 | 3.114376 |
| 198 | 9 | 0 | -3.838262 | 5.174857 | -1.340979 |
| 199 | 9 | 0 | 3.259642 | 3.905532 | -3.453139 |
| 200 | 9 | 0 | 4.438091 | 4.490076 | 1.100387 |
| 201 | 9 | 0 | -5.386648 | 0.478924 | 2.999979 |
| 202 | 9 | 0 | -7.090372 | 1.913656 | -1.183394 |

| | | | | | |
|---|---|---|---|---|---|
| 203 | 9 | 0 | 5.890706 | -0.439442 | -3.202121 |
| 204 | 9 | 0 | 6.857860 | 0.969802 | 1.218460 |

---------------------------------------------------------------------------------------------------------------------------

**V-DABNA-F-core** ($T_1$, $C_1$ symmetry)

E(B3LYP/TZP) = –41.17874542 hartree

| Center Number | Atomic Type | Coordinates (Angstroms) X | Y | Z |
|---|---|---|---|---|
| 1 | N | -1.385320 | -3.941672 | -4.768096 |
| 2 | N | -0.042269 | 2.427235 | 3.618393 |
| 3 | N | 0.000000 | 0.000000 | 7.798283 |
| 4 | N | 0.197559 | 5.290352 | -0.257669 |
| 5 | N | 0.001392 | 8.496041 | -3.886524 |
| 6 | N | 1.385320 | 3.941672 | -4.768096 |
| 7 | C | 0.018317 | -1.203975 | 4.318914 |
| 8 | N | -0.001392 | -8.496041 | -3.886524 |
| 9 | C | 0.287936 | 3.998169 | 0.281105 |
| 10 | C | -0.171935 | -1.380965 | 1.403412 |
| 11 | C | 0.000000 | 0.000000 | 6.417151 |
| 12 | C | -0.771276 | -4.568989 | -2.503865 |
| 13 | C | -0.018317 | 1.203975 | 4.318914 |
| 14 | C | 0.771276 | 4.568989 | -2.503865 |
| 15 | C | -0.938897 | -4.915333 | -3.869237 |
| 16 | C | 0.346803 | 5.590788 | -1.625111 |
| 17 | C | -1.663704 | -2.207526 | -3.040518 |
| 18 | C | 0.590988 | 2.882119 | -0.555006 |
| 19 | C | 0.171935 | 1.380965 | 1.403412 |
| 20 | C | 0.938897 | 4.915333 | -3.869237 |
| 21 | C | 1.801911 | 2.649381 | -4.388013 |
| 22 | C | 0.076237 | 2.545793 | 2.225761 |
| 23 | C | -0.346803 | -5.590788 | -1.625111 |
| 24 | C | -0.226757 | -7.187697 | -3.437250 |
| 25 | C | -1.801911 | -2.649381 | -4.388013 |
| 26 | C | 0.226757 | 7.187697 | -3.437250 |
| 27 | C | 1.663704 | 2.207526 | -3.040518 |
| 28 | C | 0.000000 | 0.000000 | 3.578993 |
| 29 | C | -0.076237 | -2.545793 | 2.225761 |
| 30 | C | -0.287936 | -3.998169 | 0.281105 |
| 31 | C | -0.590988 | -2.882119 | -0.555006 |
| 32 | N | 0.042269 | -2.427235 | 3.618393 |
| 33 | N | -0.197559 | -5.290352 | -0.257669 |
| 34 | B | 0.000000 | 0.000000 | 2.066890 |
| 35 | B | 1.011551 | 3.158890 | -2.013506 |
| 36 | B | -1.011551 | -3.158890 | -2.013506 |
| 37 | C | 0.084083 | 3.821325 | 1.654105 |
| 38 | H | -0.067352 | 4.683094 | 2.280923 |
| 39 | C | 0.462464 | 1.619167 | 0.048783 |
| 40 | H | 0.604728 | 0.756980 | -0.580249 |
| 41 | C | 2.368883 | 1.805993 | -5.361821 |
| 42 | H | 2.454033 | 2.134161 | -6.386373 |
| 43 | C | 2.840116 | 0.550228 | -5.017283 |
| 44 | H | 3.277106 | -0.081069 | -5.781886 |
| 45 | C | 2.766204 | 0.112964 | -3.694656 |
| 46 | H | 3.152594 | -0.859643 | -3.414944 |
| 47 | C | 2.188458 | 0.932972 | -2.741283 |
| 48 | H | 2.156933 | 0.586049 | -1.718970 |
| 49 | C | 0.653779 | 6.207623 | -4.331439 |

| | | | | |
|---|---|---|---|---|
| 50 | H | 0.772516 | 6.465458 | -5.373090 |
| 51 | C | 0.082838 | 6.885456 | -2.082055 |
| 52 | H | -0.233288 | 7.666720 | -1.405865 |
| 53 | C | -0.023368 | 1.208545 | 5.715597 |
| 54 | H | -0.042052 | 2.132883 | 6.274543 |
| 55 | C | 0.023368 | -1.208545 | 5.715597 |
| 56 | H | 0.042052 | -2.132883 | 6.274543 |
| 57 | C | -0.462464 | -1.619167 | 0.048783 |
| 58 | H | -0.604728 | -0.756980 | -0.580249 |
| 59 | C | -0.084083 | -3.821325 | 1.654105 |
| 60 | H | 0.067352 | -4.683094 | 2.280923 |
| 61 | C | -0.082838 | -6.885456 | -2.082055 |
| 62 | H | 0.233288 | -7.666720 | -1.405865 |
| 63 | C | -0.653779 | -6.207623 | -4.331439 |
| 64 | H | -0.772516 | -6.465458 | -5.373090 |
| 65 | C | -2.368883 | -1.805993 | -5.361821 |
| 66 | H | -2.454033 | -2.134161 | -6.386373 |
| 67 | C | -2.840116 | -0.550228 | -5.017283 |
| 68 | H | -3.277106 | 0.081069 | -5.781886 |
| 69 | C | -2.766204 | -0.112964 | -3.694656 |
| 70 | H | -3.152594 | 0.859643 | -3.414944 |
| 71 | C | -2.188458 | -0.932972 | -2.741283 |
| 72 | H | -2.156933 | -0.586049 | -1.718970 |
| 73 | H | -0.649271 | 9.021794 | -3.320611 |
| 74 | C | 1.464319 | -5.309839 | 5.537820 |
| 75 | C | -0.950059 | -5.437583 | 5.613498 |
| 76 | C | 0.299356 | -5.940787 | 5.961052 |
| 77 | H | -1.447265 | -8.784545 | 2.451289 |
| 78 | H | 2.445876 | -5.683342 | 5.796214 |
| 79 | H | 2.776874 | -8.011154 | 1.957734 |
| 80 | H | -1.868815 | -5.911656 | 5.931112 |
| 81 | C | 0.127605 | -3.621186 | 4.391416 |
| 82 | C | 1.356210 | -4.167305 | 4.762754 |
| 83 | C | -1.011343 | -4.291891 | 4.838589 |
| 84 | H | -0.238472 | 8.560564 | -4.865529 |
| 85 | H | 0.365692 | -6.834294 | 6.568560 |
| 86 | F | 2.218114 | 3.794228 | 4.504630 |
| 87 | F | 2.481229 | -3.548405 | 4.353022 |
| 88 | H | 0.016961 | -0.859500 | 8.313602 |
| 89 | H | -0.365692 | 6.834294 | 6.568560 |
| 90 | H | 0.929969 | -9.365948 | 2.959474 |
| 91 | F | -2.481229 | 3.548405 | 4.353022 |
| 92 | F | 2.422518 | -6.003744 | 0.380403 |
| 93 | F | -2.218114 | -3.794228 | 4.504630 |
| 94 | F | 2.178735 | 6.840724 | 0.925373 |
| 95 | C | 1.488117 | 4.306162 | -6.159161 |
| 96 | C | 2.652438 | 4.897317 | -6.642969 |
| 97 | C | 0.416453 | 4.066535 | -7.014368 |
| 98 | C | 2.742562 | 5.251017 | -7.985810 |
| 99 | H | 3.477819 | 5.075945 | -5.965421 |
| 100 | C | 0.510198 | 4.420784 | -8.357254 |
| 101 | H | -0.479978 | 3.602334 | -6.623147 |
| 102 | C | 1.672000 | 5.013710 | -8.844580 |
| 103 | H | 3.649141 | 5.710415 | -8.360542 |
| 104 | H | -0.324223 | 4.232211 | -9.021839 |
| 105 | H | 1.743783 | 5.288405 | -9.889995 |
| 106 | C | -1.488117 | -4.306162 | -6.159161 |
| 107 | C | -2.652438 | -4.897317 | -6.642969 |
| 108 | C | -0.416453 | -4.066535 | -7.014368 |
| 109 | C | -2.742562 | -5.251017 | -7.985810 |

| | | | | |
|---|---|---|---|---|
| 110 | H | -3.477819 | -5.075945 | -5.965421 |
| 111 | C | -0.510198 | -4.420784 | -8.357254 |
| 112 | H | 0.479978 | -3.602334 | -6.623147 |
| 113 | C | -1.672000 | -5.013710 | -8.844580 |
| 114 | H | -3.649141 | -5.710415 | -8.360542 |
| 115 | H | 0.324223 | -4.232211 | -9.021839 |
| 116 | H | -1.743783 | -5.288405 | -9.889995 |
| 117 | H | 0.238472 | -8.560564 | -4.865529 |
| 118 | C | -0.108312 | 6.370017 | 0.620251 |
| 119 | F | -2.422518 | 6.003744 | 0.380403 |
| 120 | C | -1.422641 | 6.723911 | 0.927226 |
| 121 | F | -2.178735 | -6.840724 | 0.925373 |
| 122 | H | -0.929969 | 9.365948 | 2.959474 |
| 123 | H | -0.016961 | 0.859500 | 8.313602 |
| 124 | C | 0.895263 | 7.146871 | 1.199701 |
| 125 | C | -0.701197 | 8.531034 | 2.309612 |
| 126 | H | -2.776874 | 8.011154 | 1.957734 |
| 127 | H | 1.447265 | 8.784545 | 2.451289 |
| 128 | H | 0.649271 | -9.021794 | -3.320611 |
| 129 | C | -1.464319 | 5.309839 | 5.537820 |
| 130 | C | 0.950059 | 5.437583 | 5.613498 |
| 131 | C | -0.299356 | 5.940787 | 5.961052 |
| 132 | C | -1.738171 | 7.785199 | 1.758382 |
| 133 | H | -2.445876 | 5.683342 | 5.796214 |
| 134 | C | 0.625621 | 8.217703 | 2.035082 |
| 135 | H | 1.868815 | 5.911656 | 5.931112 |
| 136 | C | -0.127605 | 3.621186 | 4.391416 |
| 137 | C | -1.356210 | 4.167305 | 4.762754 |
| 138 | C | 1.011343 | 4.291891 | 4.838589 |
| 139 | C | 0.108312 | -6.370017 | 0.620251 |
| 140 | C | -0.895263 | -7.146871 | 1.199701 |
| 141 | C | 1.422641 | -6.723911 | 0.927226 |
| 142 | C | -0.625621 | -8.217703 | 2.035082 |
| 143 | C | 1.738171 | -7.785199 | 1.758382 |
| 144 | C | 0.701197 | -8.531034 | 2.309612 |

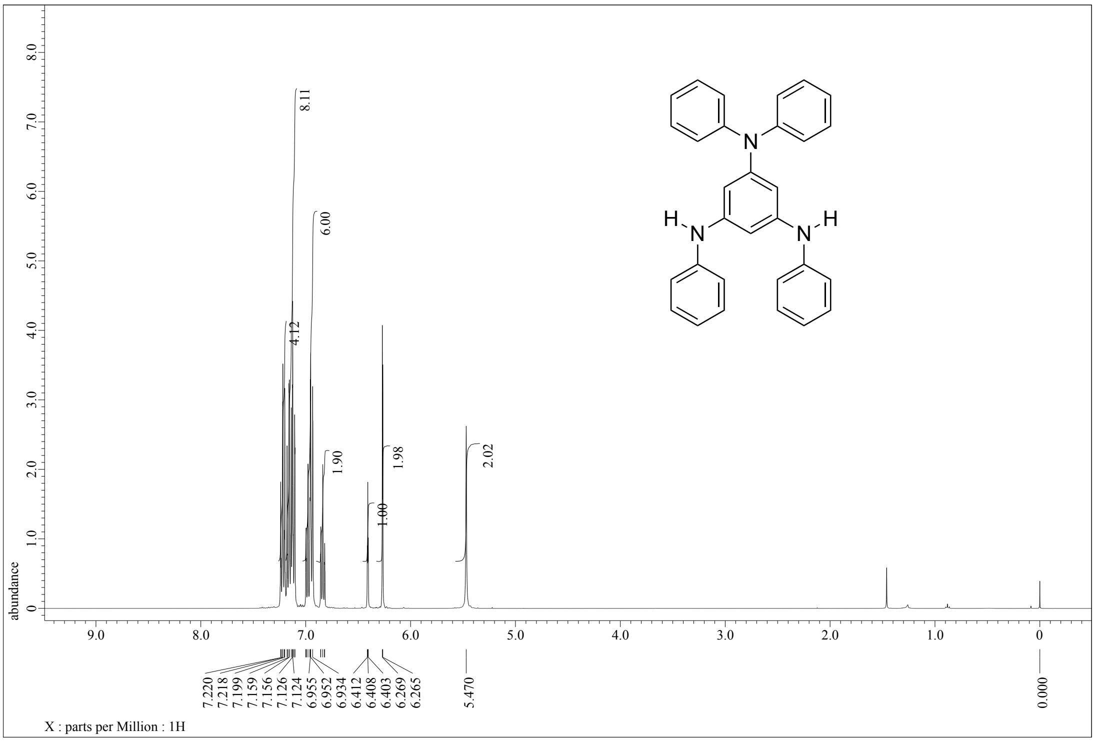


**Figure S12.** $^{1}$H NMR spectrum of **2** in $CDCl_3$ at 25 °C.

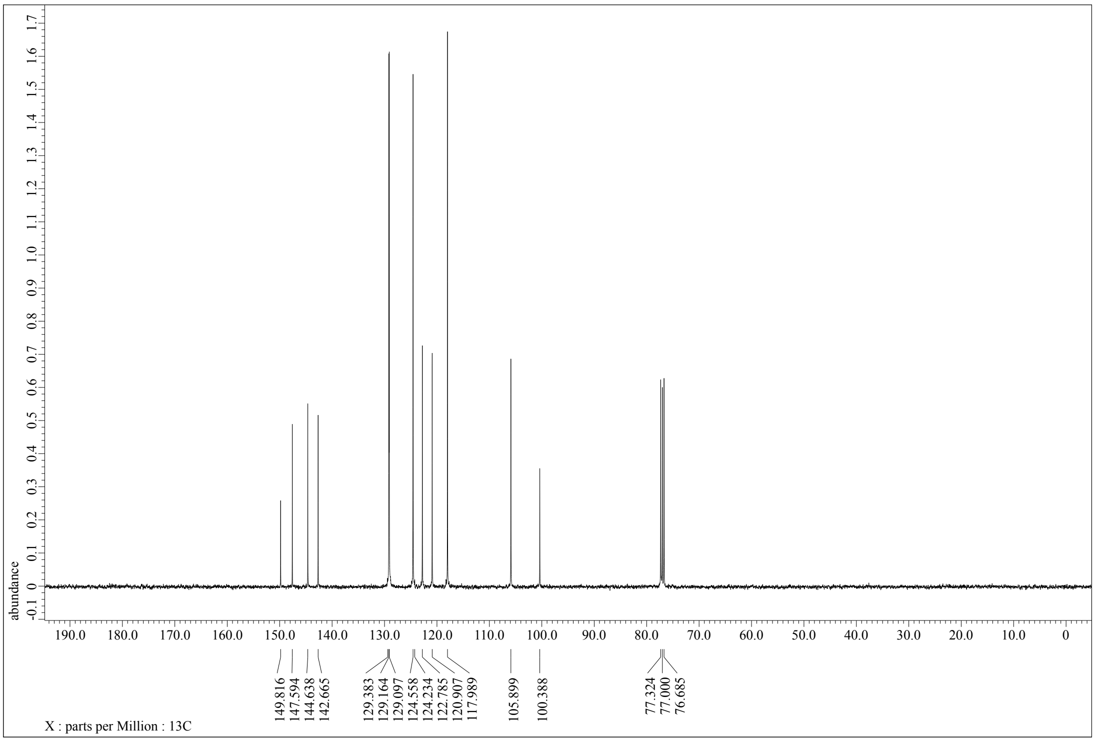


**Figure S13.** $^{13}$C NMR spectrum of **2** in $CDCl_3$ at 25 °C.

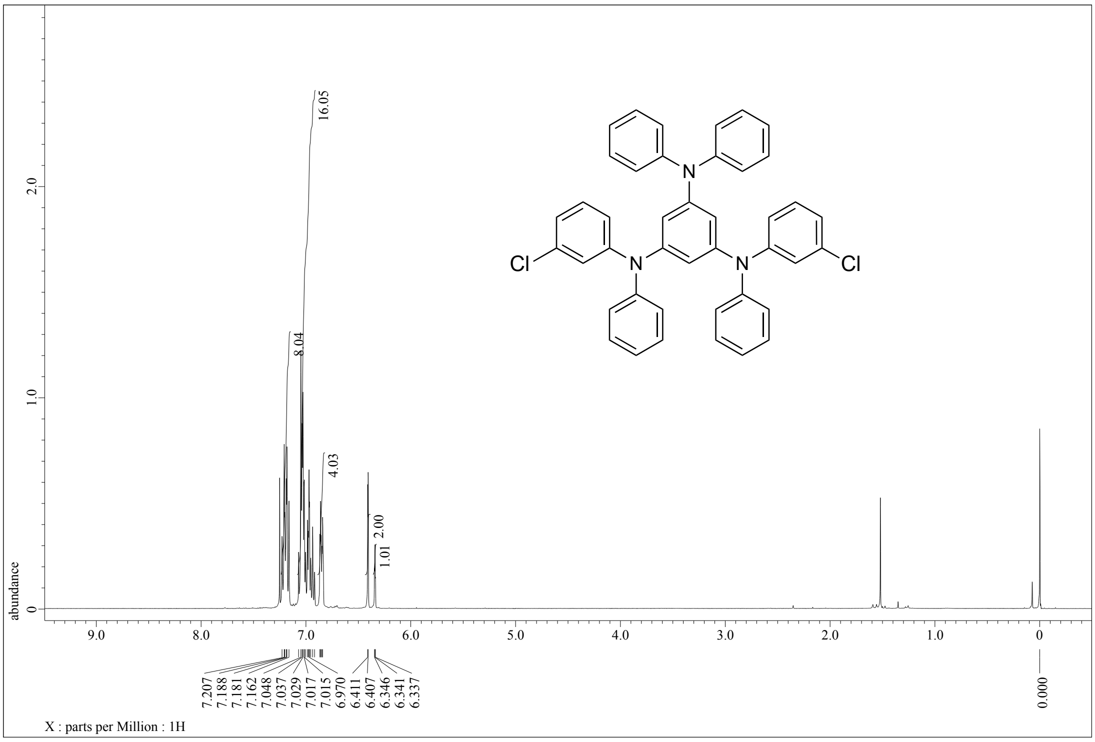


**Figure S14.** $^{1}$H NMR spectrum of **3** in $CDCl_3$ at 25 °C.

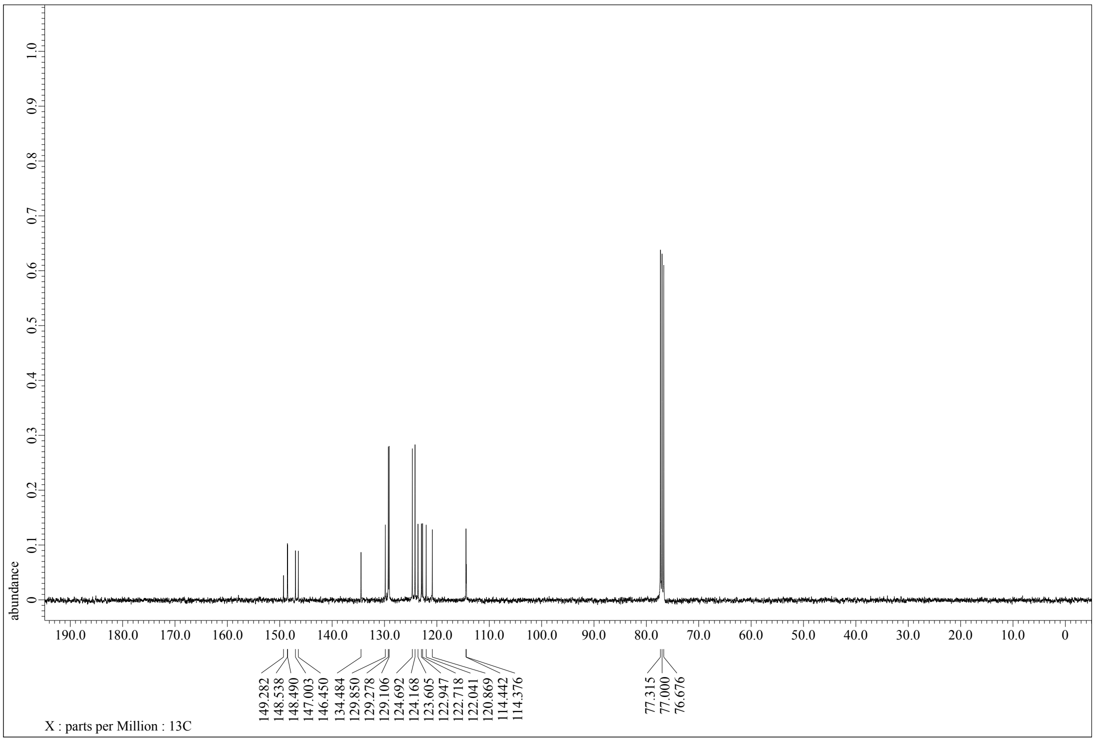


**Figure S15.** $^{13}$C NMR spectrum of **3** in $CDCl_3$ at 25 °C.

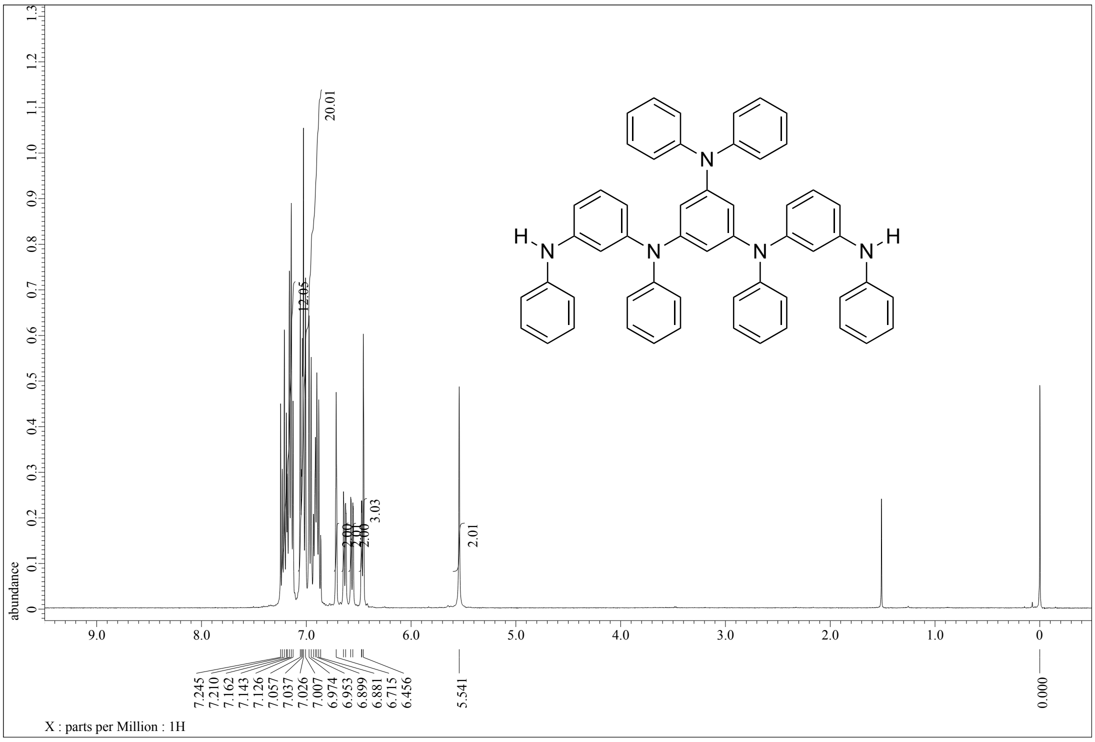

**Figure S16.** $^1$H NMR spectrum of **4** in $CDCl_3$ at 25 °C.

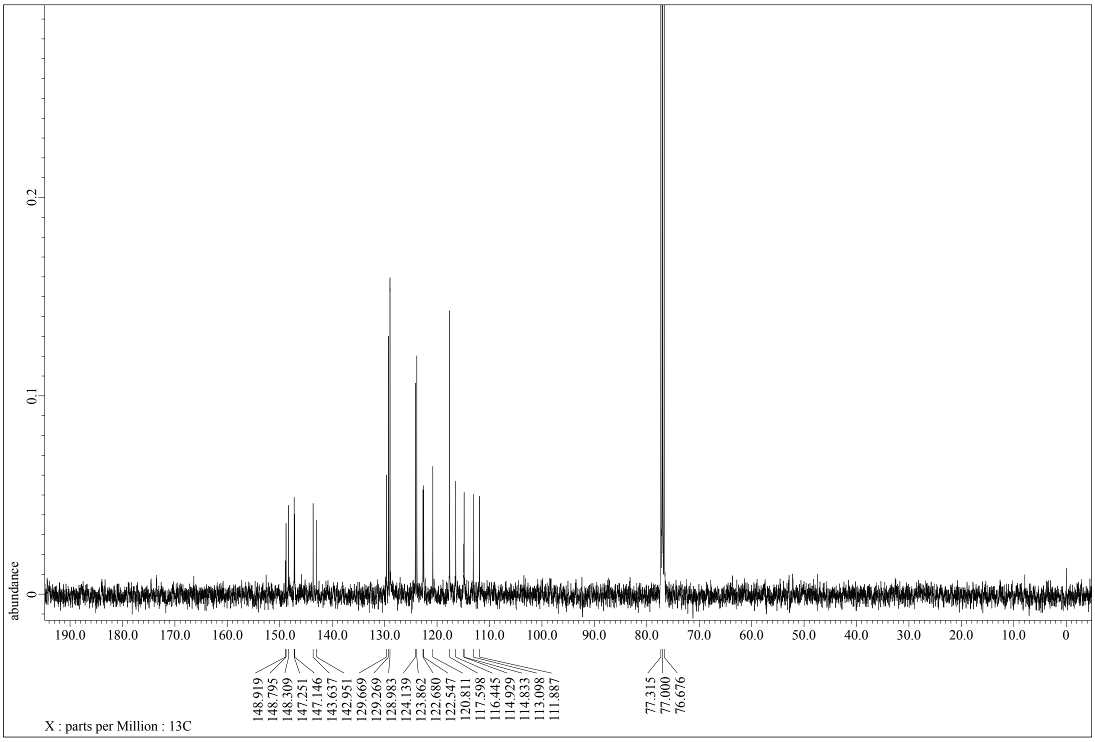

**Figure S17.** $^{13}$C NMR spectrum of **4** in $CDCl_3$ at 25 °C.

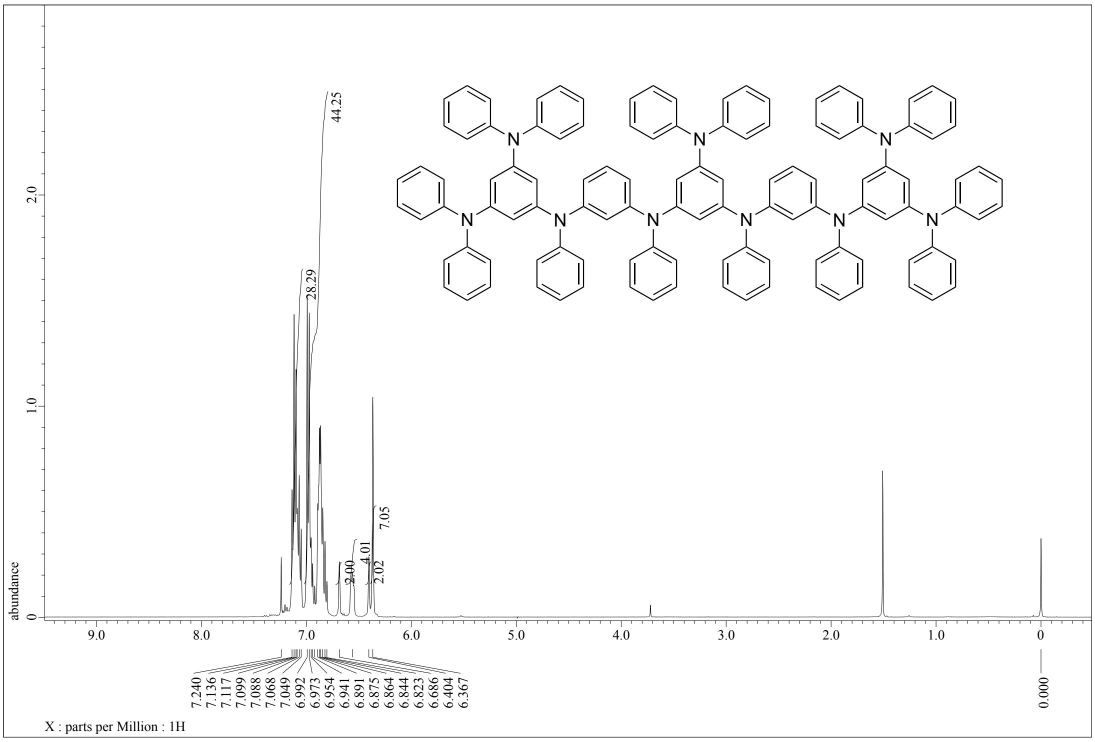


**Figure S18.** $^{1}$H NMR spectrum of **5** in $CDCl_3$ at 25 °C.

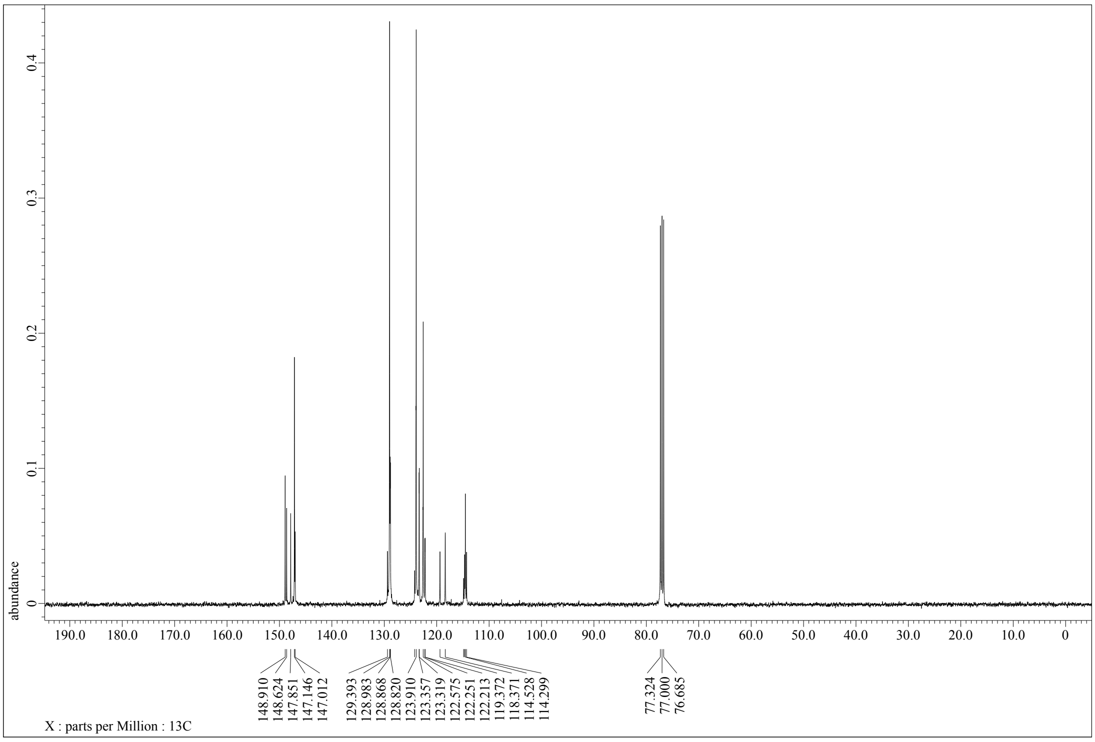


**Figure S19.** $^{13}$C NMR spectrum of **5** in $CDCl_3$ at 25 °C.

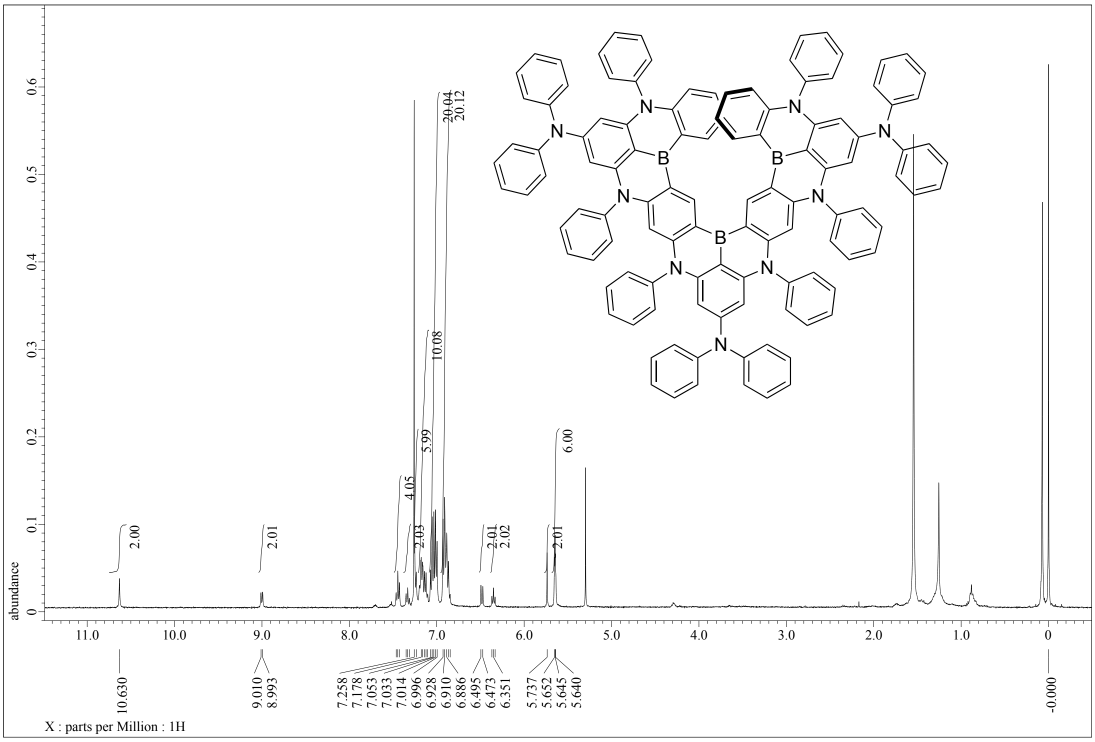

**Figure S20.** $^{1}$H NMR spectrum of **V-DABNA** in $CDCl_3$ at 25 °C.

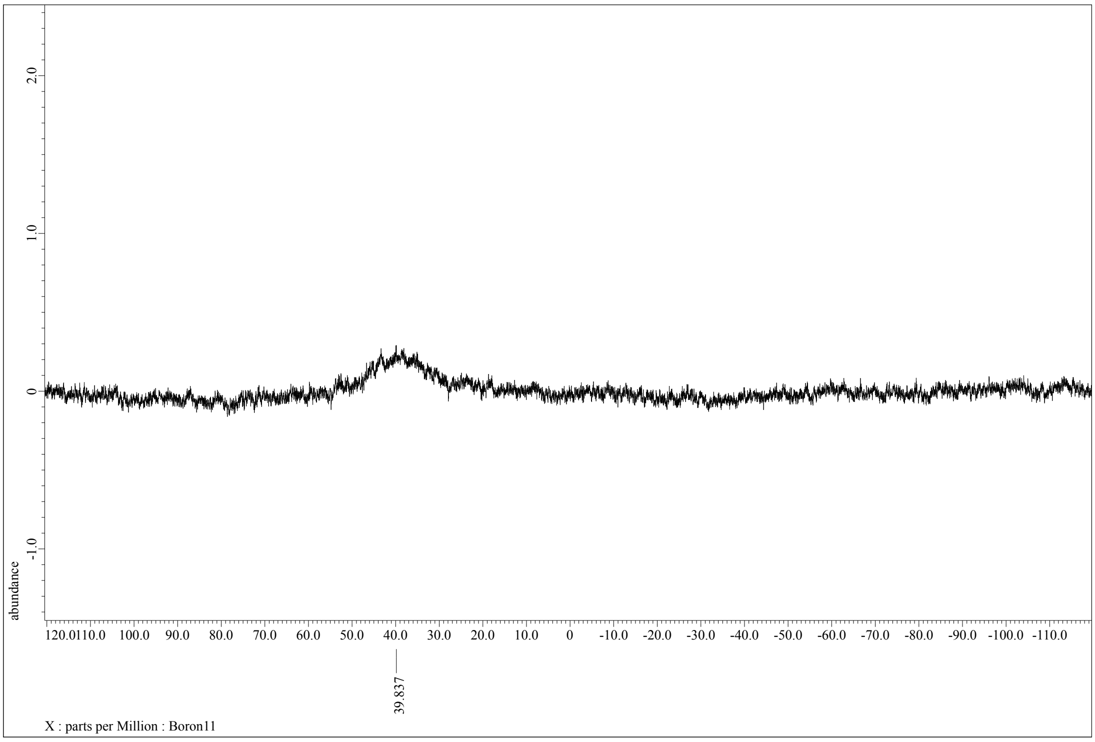

**Figure S21.** $^{11}$B NMR spectrum of **V-DABNA** in $C_6D_4Cl_2$ at 100 °C.

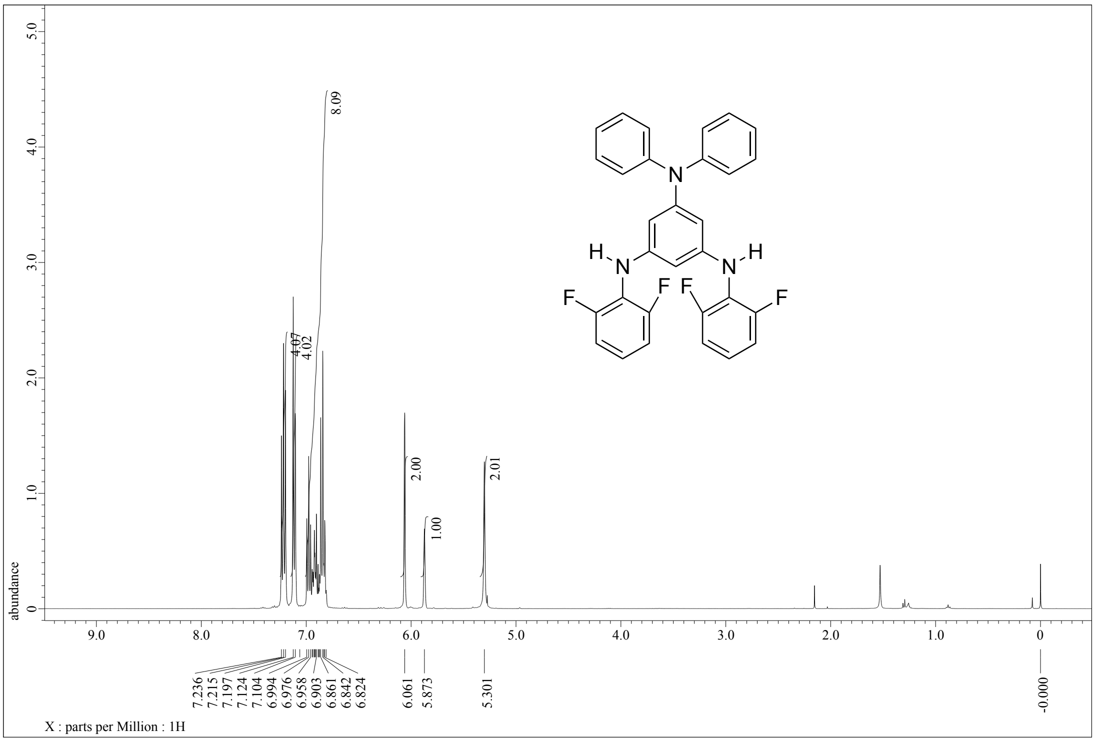


**Figure S22.** $^{1}H$ NMR spectrum of **2-F** in $CDCl_3$ at 25 °C.

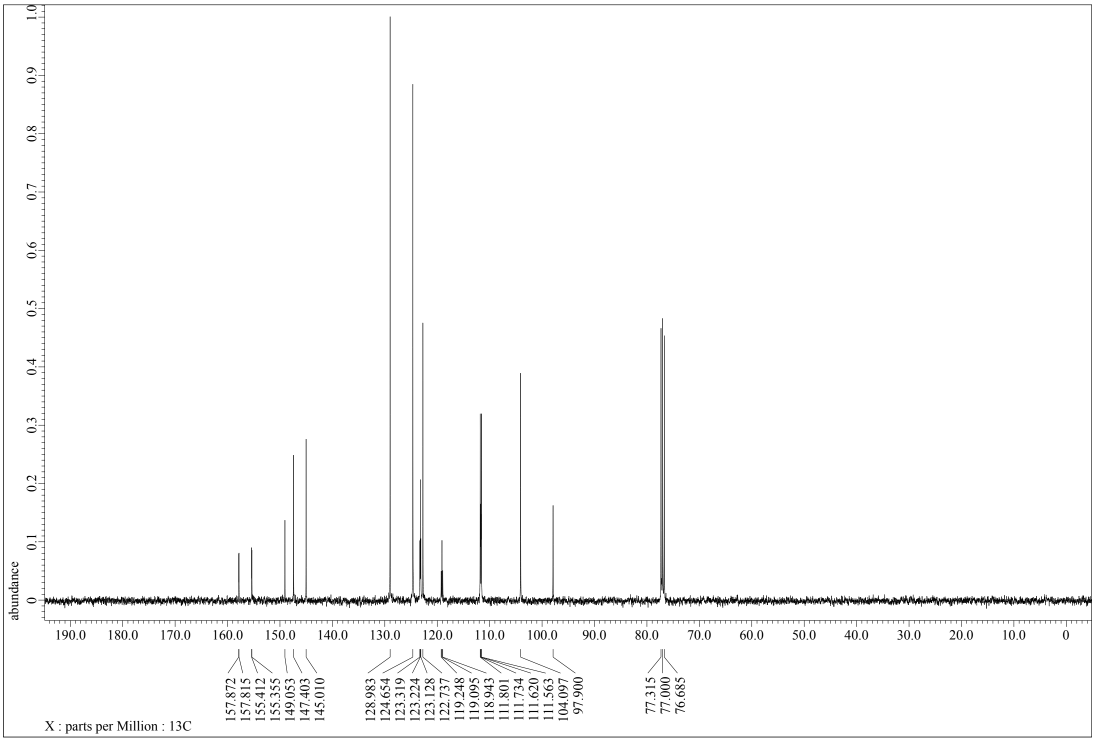


**Figure S23.** $^{13}C$ NMR spectrum of **2-F** in $CDCl_3$ at 25 °C.

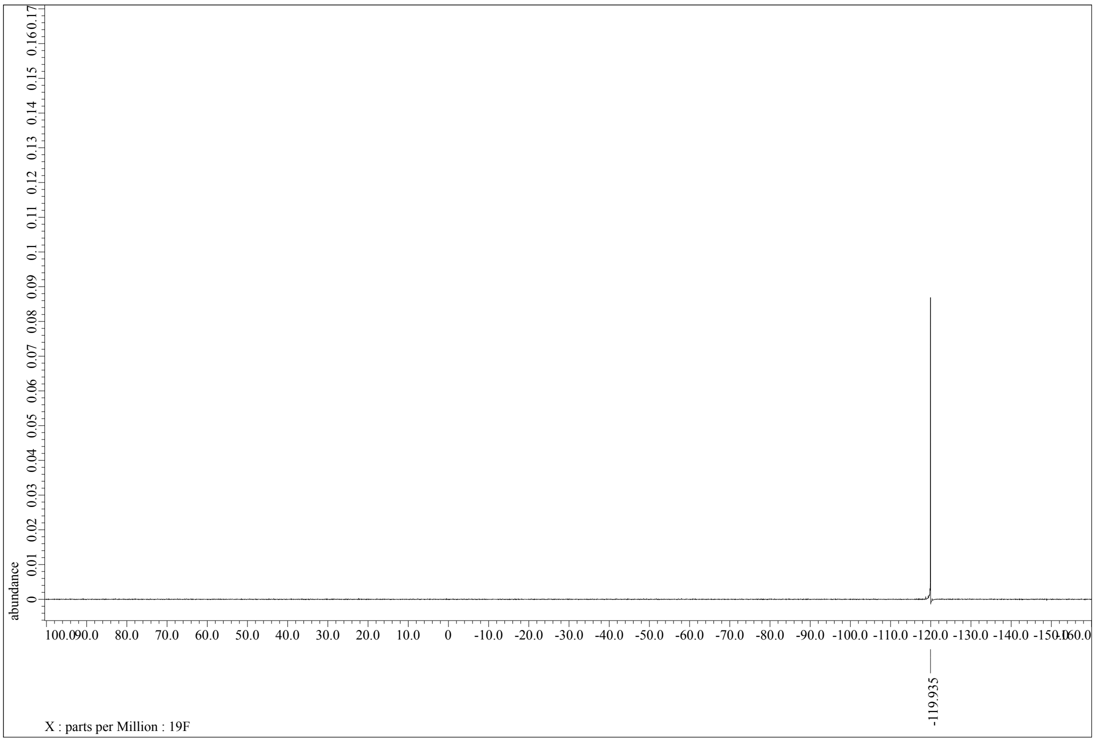


**Figure S24.** $^{19}$F NMR spectrum of **2-F** in $CDCl_3$ at 25 °C.

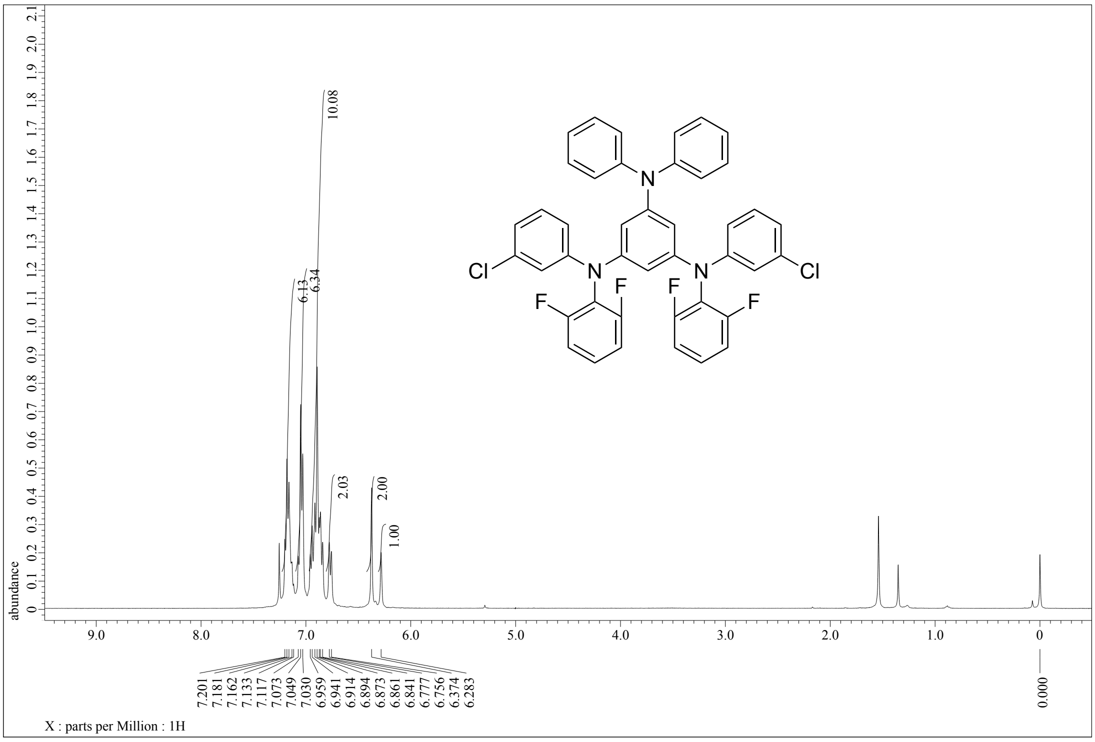


**Figure S25.** $^{1}$H NMR spectrum of **3-F** in $CDCl_3$ at 25 °C.

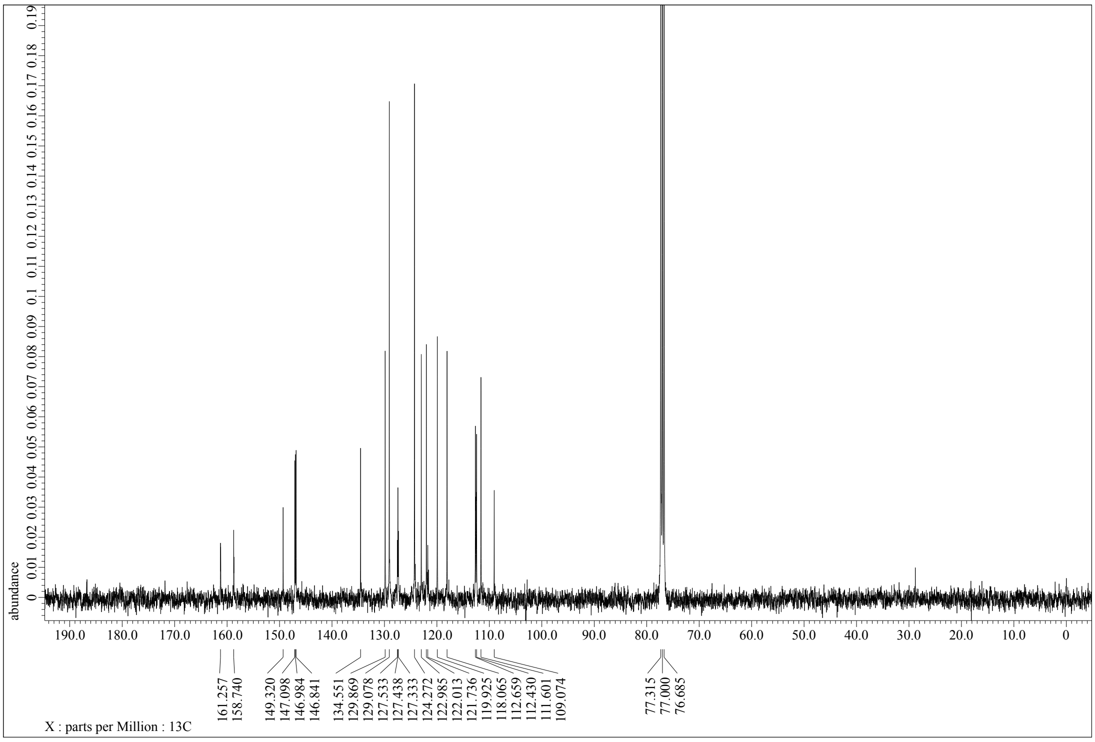


**Figure S26.** $^{13}$C NMR spectrum of **3-F** in $CDCl_3$ at 25 °C.

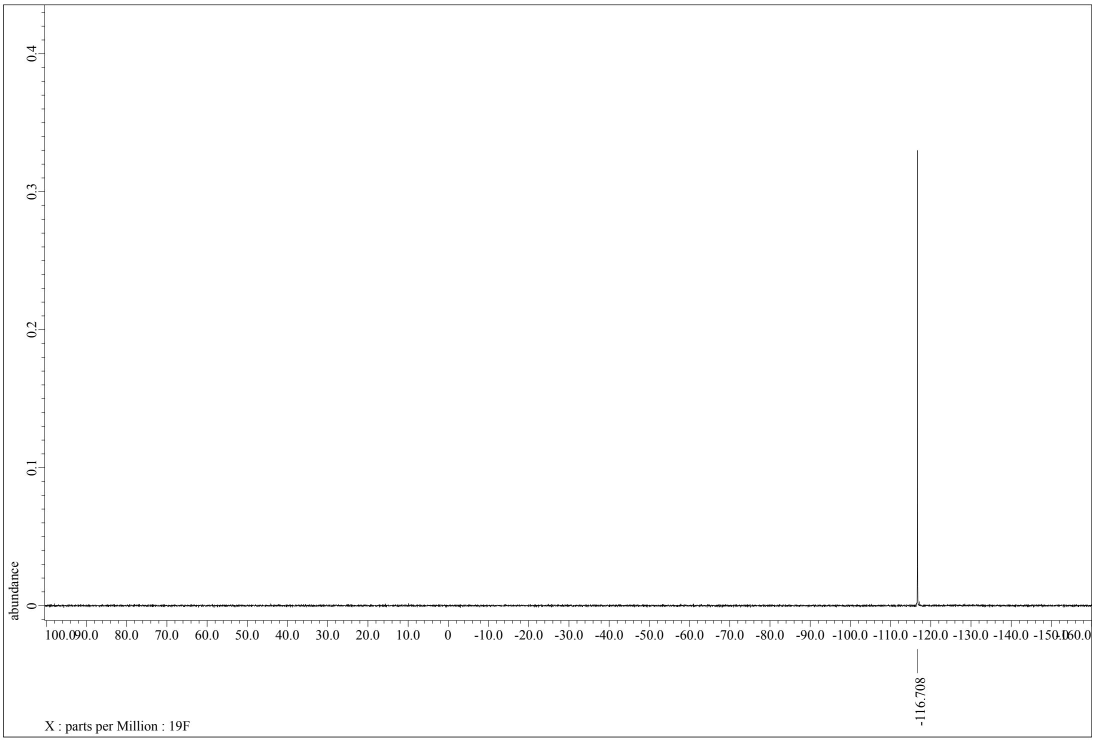


**Figure S27.** $^{19}$F NMR spectrum of **3-F** in $CDCl_3$ at 25 °C.

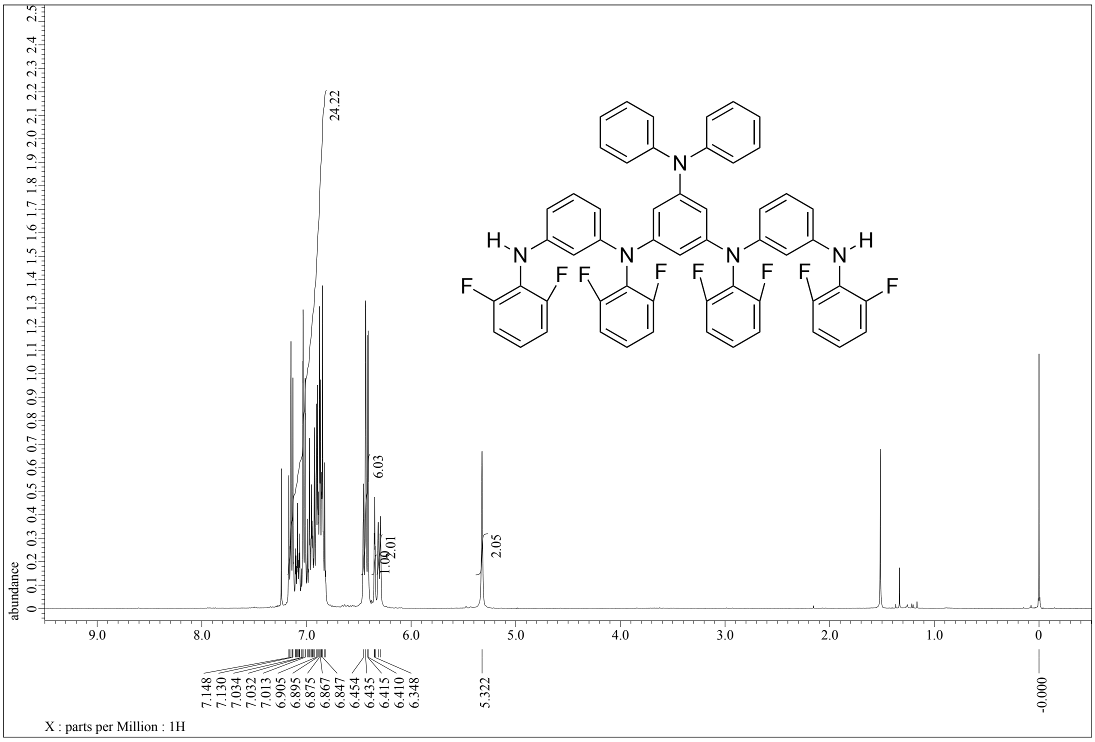


**Figure S28.** $^{1}$H NMR spectrum of **4-F** in $CDCl_3$ at 25 °C.

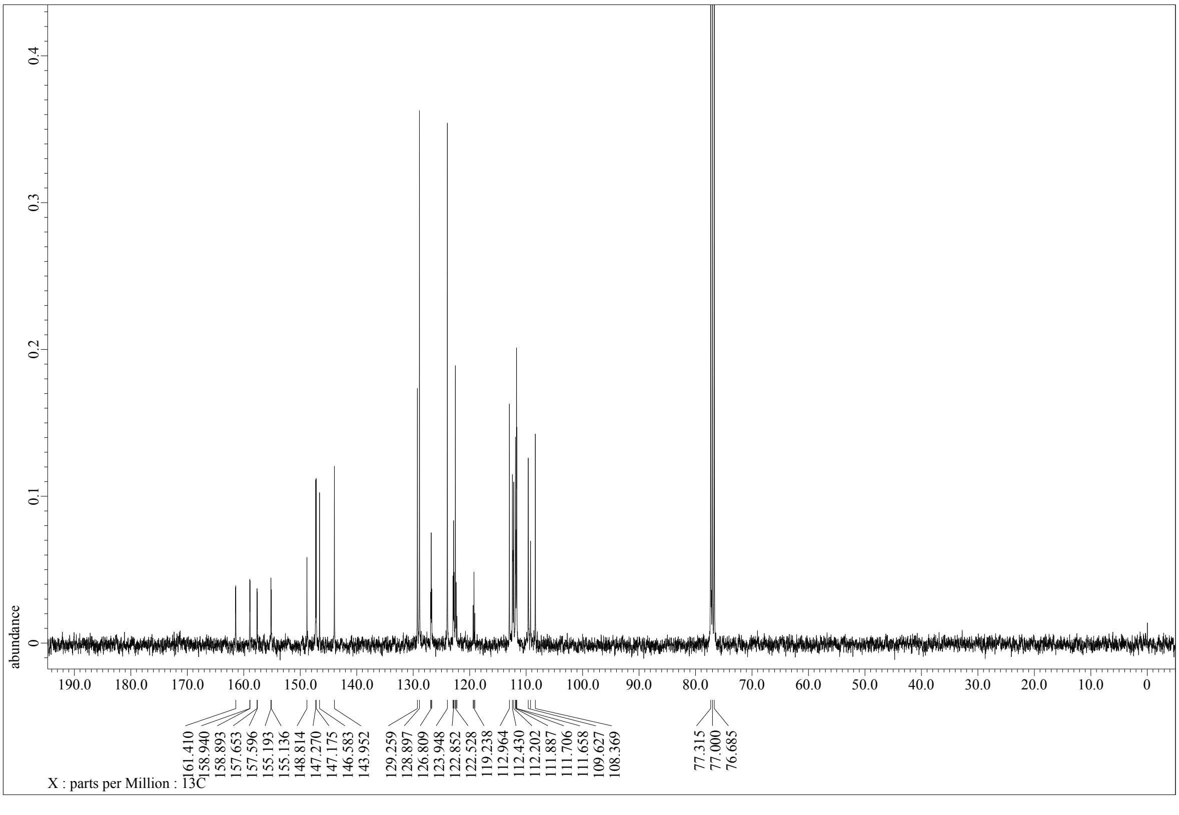


**Figure S29.** $^{13}$C NMR spectrum of **4-F** in $CDCl_3$ at 25 °C.

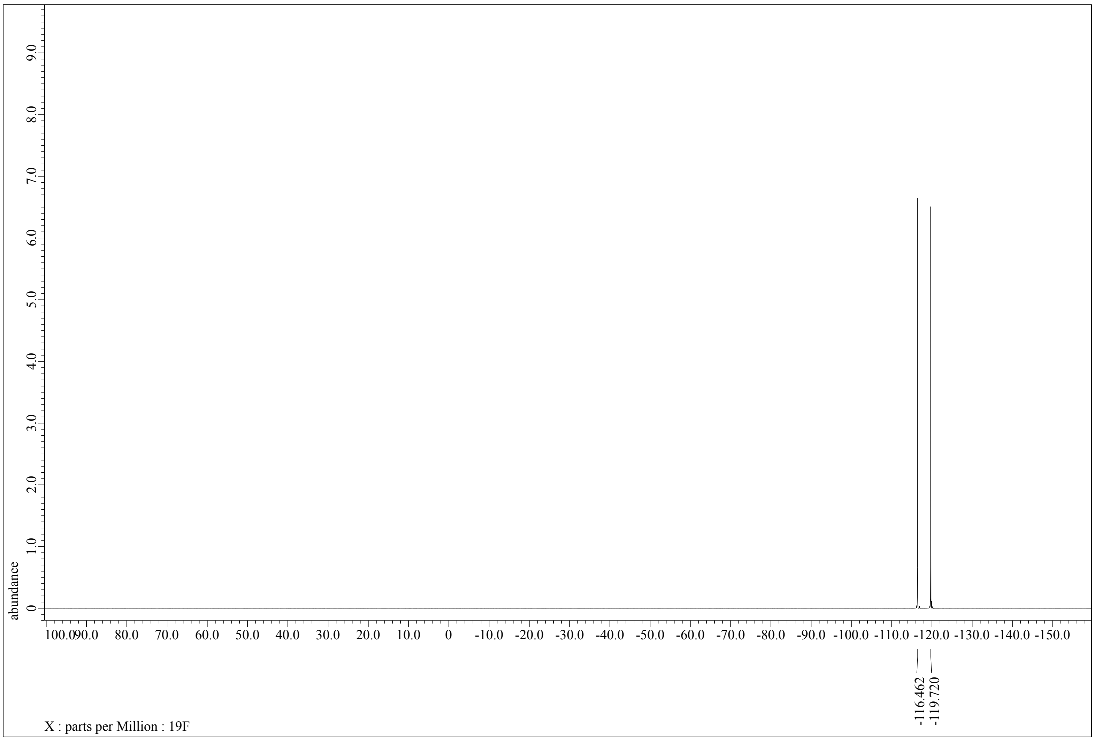


**Figure S30.** $^{19}$F NMR spectrum of **4-F** in $CDCl_3$ at 25 °C.

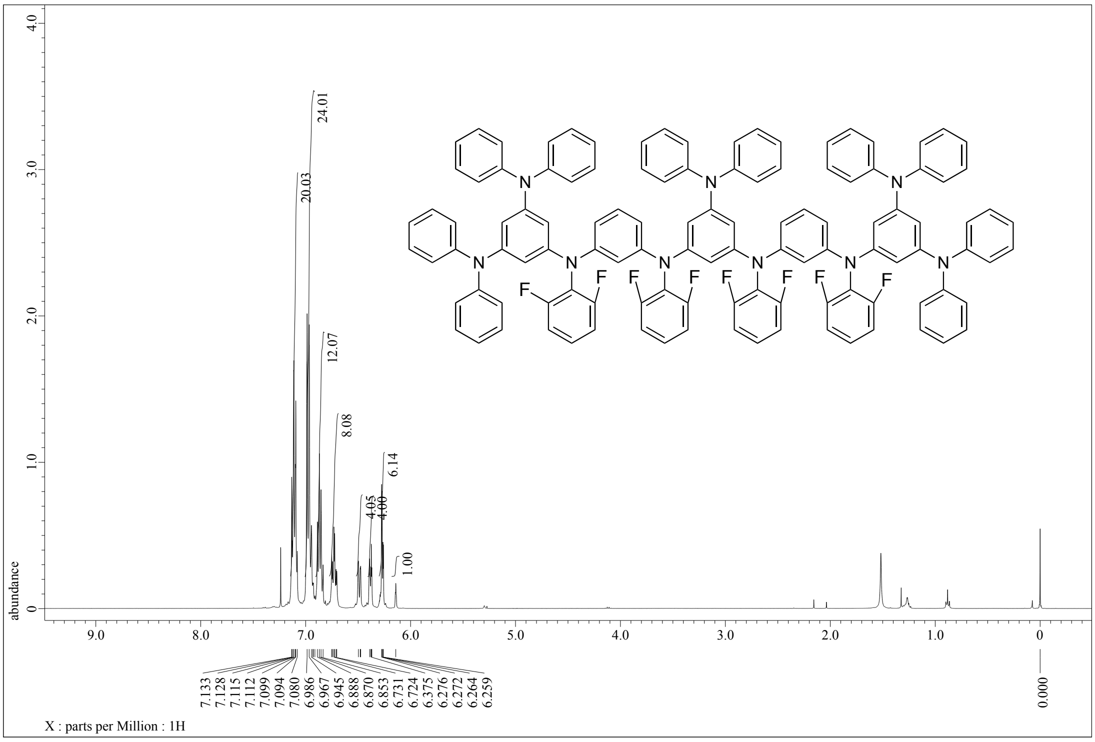


**Figure S31.** $^1$H NMR spectrum of **5-F** in $CDCl_3$ at 25 °C.

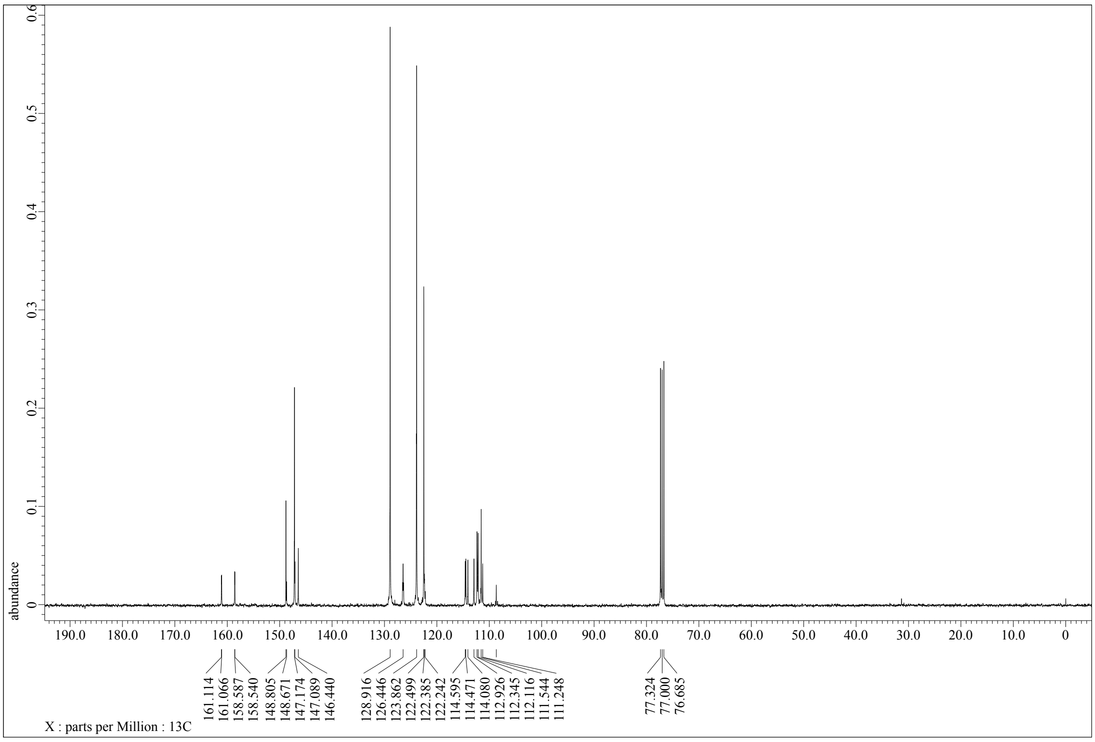


**Figure S32.** $^{13}C$ NMR spectrum of **5-F** in $CDCl_3$ at 25 °C.

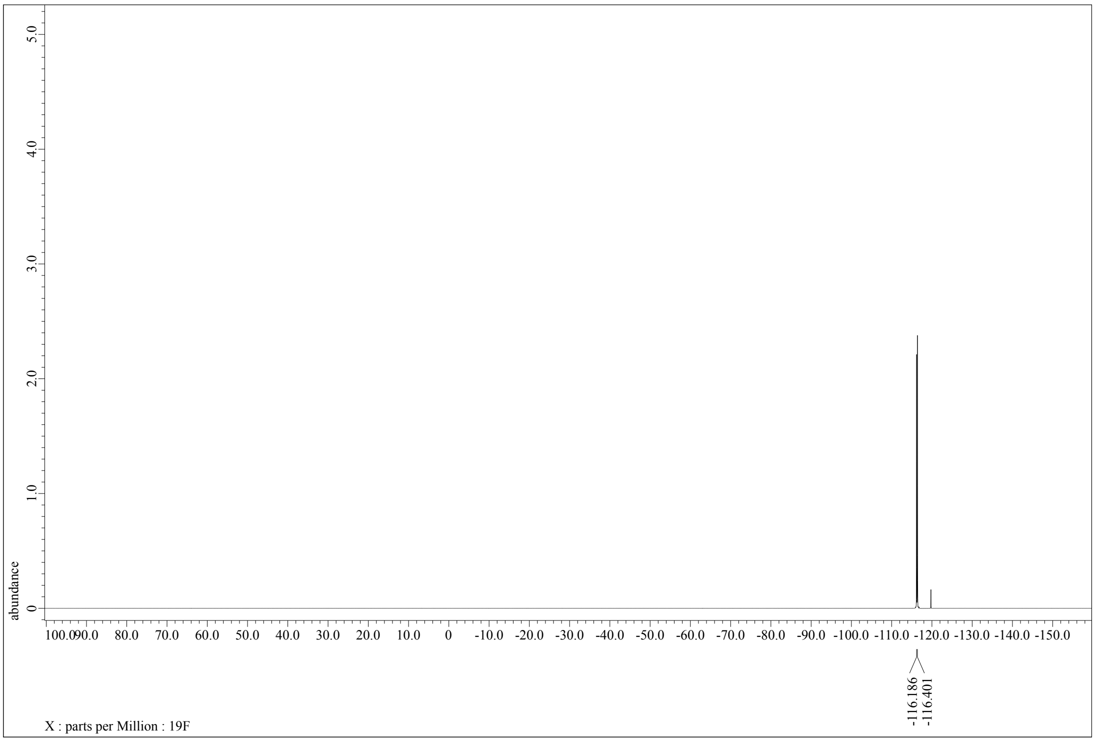


**Figure S33.** $^{19}F$ NMR spectrum of **5-F** in $CDCl_3$ at 25 °C.

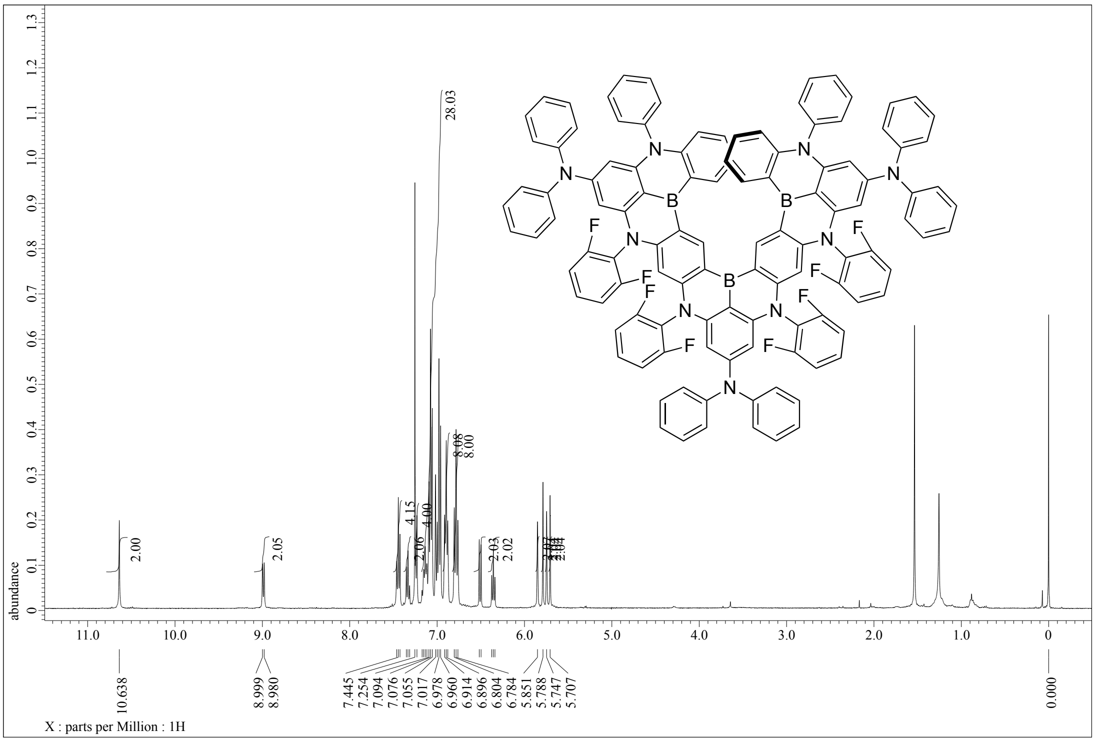

**Figure S34.** $^{1}$H NMR spectrum of **V-DABNA-F** in $CDCl_3$ at 25 °C.

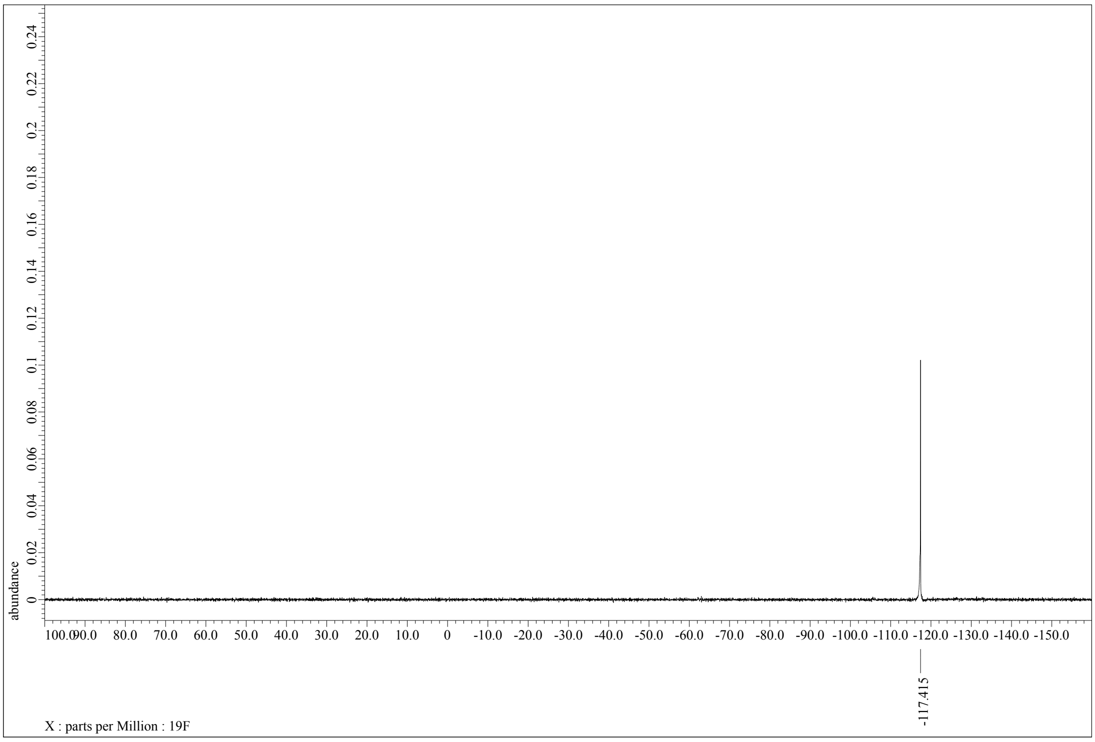

**Figure S35.** $^{19}$F NMR spectrum of **V-DABNA-F** in $(CDCl_2)_2$ at 25 °C.

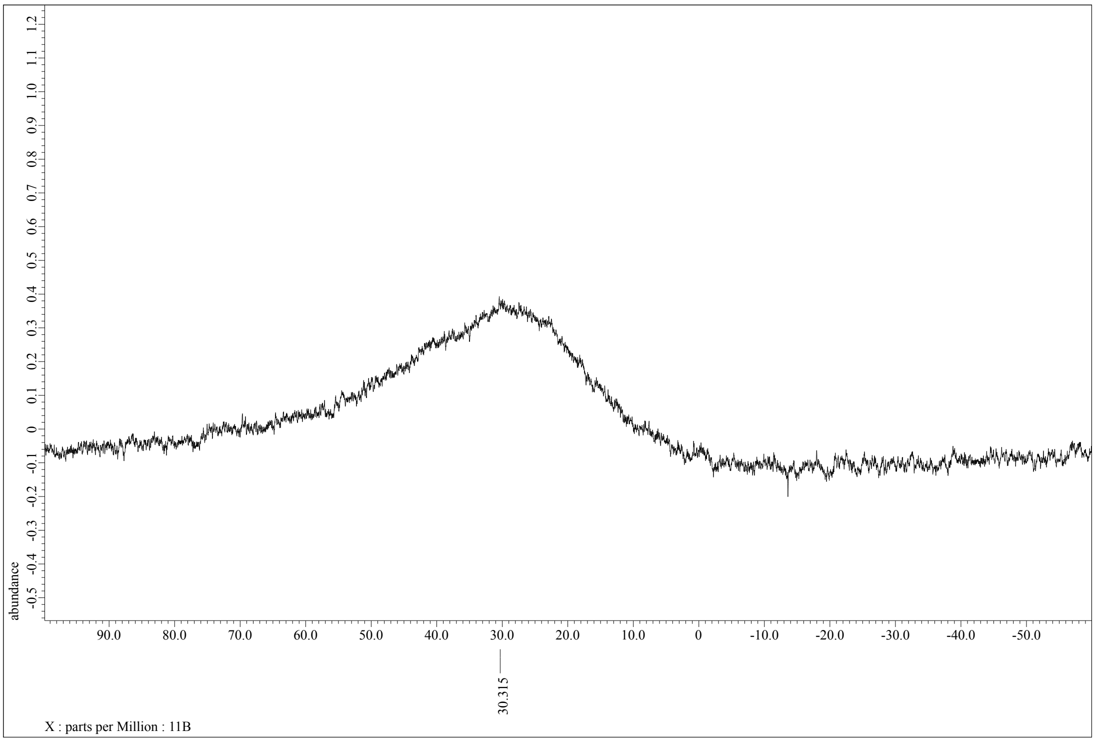


**Figure S36.** $^{11}$B NMR spectrum of **V-DABNA-F** in $(CDCl_2)_2$ at 100 °C.
